# A role for Ca_V_1 and calcineurin signaling in depolarization-induced changes in neuronal DNA methylation

**DOI:** 10.1016/j.nepig.2015.06.001

**Published:** 2015-06-25

**Authors:** Eilis Hannon, Annisa N. Chand, Mark D. Evans, Chloe C.Y. Wong, Matthew S. Grubb, Jonathan Mill

**Affiliations:** a University of Exeter Medical School, University of Exeter, Exeter, UK; b MRC Centre for Developmental Neurobiology, King's College London, London, UK; c MRC Social, Genetic & Developmental Psychiatry Centre, Institute of Psychiatry, Psychology & Neuroscience, King's College London, London UK

**Keywords:** DNA methylation, Neuronal activity, Calcium channels, Reduced representation bisulfite sequencing, Hippocampal, Pharmacological manipulation

## Abstract

Direct manipulations of neuronal activity have been shown to induce changes in DNA methylation (DNAm), although little is known about the cellular signaling pathways involved. Using reduced representation bisulfite sequencing, we identify DNAm changes associated with moderate chronic depolarization in dissociated rat hippocampal cultures. Consistent with previous findings, these changes occurred primarily in the vicinity of loci implicated in neuronal function, being enriched in intergenic regions and underrepresented in CpG-rich promoter regulatory regions. We subsequently used 2 pharmacological interventions (nifedipine and FK-506) to test whether the identified changes depended on 2 interrelated signaling pathways known to mediate multiple forms of neuronal plasticity. Both pharmacological manipulations had notable effects on the extent and magnitude of depolarization-induced DNAm changes indicating that a high proportion of activity-induced changes are likely to be mediated by calcium entry through L-type Ca_V_1 channels and/or downstream signaling via the calcium-dependent phosphatase calcineurin.

## 1 Introduction

Epigenetic processes act to dynamically control gene expression independently of DNA sequence variation and are known to regulate key neurobiological and cognitive processes including brain development (Numata et al., 2012, Lister et al., 2013, Spiers et al., in press), circadian processes (Azzi et al., 2014), synaptic function (Nelson et al., 2008, Feng et al., 2010), and memory formation (Day and Sweatt, 2010, Zovkic et al., 2013). Interindividual variation in epigenetic modifications in the brain is associated with a number of neuropsychiatric and neurodegenerative disorders (De Jager et al., 2014, Lunnon et al., 2014, Numata et al., 2014, Pidsley et al., 2014). DNA methylation (DNAm), typically in the context of palindromic 5′-CpG-3′ dinucleotides and more rarely in a non-CpG context, is the most extensively studied epigenetic modification, playing a role in many important genomic regulatory processes. The covalently attached methyl groups project into the major groove of DNA where they can inhibit transcription by blocking the binding of transcription factors and by recruiting methyl-CpG binding proteins such as MECP2, which remodel chromatin into a condensed heterochromatic state.

Although dynamic changes in DNAm were believed to only occur in dividing cells, recent studies support a role for active methylation (and demethylation) in postmitotic neurons. Direct manipulations of neuronal activity in vitro and in vivo have been shown to induce significant de novo DNAm and/or active demethylation across the genome (Nelson et al., 2008, Martinowich et al., 2003, Ma et al., 2009, Guo et al., 2011a, Guo et al., 2011b), whereas significant neuronal DNAm changes in the brain have been associated with a wide range of learning outcomes and behavioral modifications (eg, Weaver et al., 2004, Lubin et al., 2008, McGowan et al., 2009, Murgatroyd et al., 2009). Little is currently known, however, about the cellular signaling pathways linking changes in electrical activity to alterations in DNAm. Here we test the contribution to activity-dependent DNAm of 2 highly interrelated pathways known to mediate multiple forms of neuronal plasticity: calcium entry via voltage-gated L-type Ca_V_1 channels and downstream signaling via the calcium-dependent phosphatase calcineurin (Zeng et al., 2001, Deisseroth et al., 2003, Misonou et al., 2004, Greer and Greenberg, 2008, Schwartz et al., 2009, Schonewille et al., 2010, Wheeler et al., 2012, Evans et al., 2013). We use dissociated hippocampal cultures to identify DNAm changes associated with moderate chronic depolarization, using pharmacological interventions to demonstrate that a notable subset of the identified changes depend on signaling through these 2 pathways.

## 2 Materials and methods

### 2.1 Dissociated culture, depolarization and DNA extraction

We dissected hippocampi from embryonic day (E) 18 Wistar rat embryos of either sex (Charles River, UK) into Hank's Balanced Salt Solution. Tissue was digested with trypsin (Worthington, 0.5 mg/mL; 15 minutes at 37°C) before trituration and subsequent plating at 90,000 cells/well directly into plastic 12-well plates precoated with poly-l-lysine (50 μg/mL; Sigma, Gillingham, UK) and laminin (40 μg/mL). Neurons were cultured at 37°C with 5% CO_2_ in neurobasal medium supplemented with 1% B27, 1% fetal calf serum, and 500 μmol/L Glutamax. At 4 and 7 days in vitro (DIV), half of the media was changed with media supplemented with 2% B27 and 500 μmol/L Glutamax. Unless otherwise stated, all culture reagents were from Invitrogen (Paisley, UK). For chronic depolarization, we treated cultures at 10 DIV for 24 hours with + 10 mmol/L KCl or + 10 mmol/L NaCl as an osmolarity control. Pharmacological agents were stored in stock solutions in Dimethyl Sulfoxide (DMSO) and then added at previously described effective final working concentrations (1 μmol/L nifedipine in 0.001% DMSO; 1 μmol/L FK506 in 0.1% DMSO) at least 30 minutes before control or depolarizing treatment. Cells were washed in Hank's Balanced Salt Solution and then exposed to 0.15% trypsin for 1 minute at 37°C. Cells were subsequently scraped and aspirated into collection tubes containing neurobasal medium with 2% fetal calf serum for trypsin deactivation. After centrifugation at 1700 rpm for 5 minutes, DNA was extracted using the Qiagen AllPrep kit and eluted in RNAse-free water. DNA samples were checked for purity and degradation, before storage at − 80°C.

### 2.2 Reduced represented bisulfite sequencing

Reduced represented bisulfite sequencing (RRBS) was undertaken using a standard published protocol (Gu et al., 2011), and the 8 individual libraries were sequenced on an Illumina HiSeq2500 sequencer. An average of 40 million high-quality 49-bp paired end reads was obtained for each individual sample. The quality of all RRBS reads were visualized and assessed using FastQC (http://www.bioinformatics.babraham.ac.uk/projects/fastqc/) with all samples being deemed of satisfactory quality (Phred score > 30) to be included in this study (see Supplementary Fig. 1). *Trim galore* (http://www.bioinformatics.babraham.ac.uk/projects/trim_galore/) was used to remove any low-quality called bases at the end of sequences. Bismark (Krueger and Andrews, 2011) was used to in silico bisulfite convert the rat reference genome (rn4). On average 59% (SD, 3.77%) could be uniquely aligned to the bisulfite-converted rat genome using custom scripts developed for RRBS data. DNA methylation at all cytosine bases were quantified using the --*comprehensive*, --*CX*, and --*no-overlap* flags.

### 2.3 Statistical analysis

Cytosine sites were filtered to those with a minimum coverage of 10 reads in each of the 8 RRBS data sets. Cytosine sites showing consistent changes after depolarization were identified from 2 experimental replicates, which both had a change in DNAm of greater than 20% in the same direction. These changes were then defined as “blocked” if the addition of either FK506 or nifedipine meant that a change of at least 20% (in the same direction) was not observed between the unstimulated drug-naive sample and stimulated drug-treated sample. Gene annotation files for rn4 were downloaded from University of California, Santa Cruz (UCSC) and were used to annotate whether sites were found in exons, introns, transcription start sites (defined as 5-kb upstream), or transcription end sites (defined as 5-kb downstream). Any remaining sites not annotated to one of these categories were classed as intergenic and the nearest gene within 10 kb identified. In addition, CpG island annotation was downloaded from UCSC and used to identify which sites were found in CpG islands or shores. Ontology pathways were downloaded from the Gene Ontology website (http://geneontology.org/). All sites located within 10 kb of a gene annotated to at least 1 ontology pathway were mapped to pathways, including all parent terms. Overrepresentation for any pathway, with at least 10 genes, in the sites with consistent large epigenetic changes was tested with a Fisher exact test compared to the number of sites passing quality control filtering annotated to each pathway in the same manner. All statistical analyses were performed in the R statistical language.

### 2.4 Calcium imaging

Calcium imaging was carried out on hippocampal cultures plated onto 18-mm glass coverslips precoated with poly-d-lysine (50 μg/mL; Sigma) and laminin (40 μg/mL) at 90,000 cells/well. We loaded cells with the ratiometric calcium indicator fura-2-AM (5 μmol/L; Invitrogen) in a HEPES-buffered saline solution containing, in millimoles per liter: 136 NaCl, 2.5 KCl, 10 HEPES, 10 d-glucose, 2 CaCl_2_, and 1.3 MgCl_2_ (30 minutes at room temperature). After washing, cells were incubated in phenol red-free neurobasal medium (Invitrogen) with or without 1 μmol/L nifedipine (30 minutes at 37°C). Cells were then maintained in a steady gravity-fed flow of phenol red-free neurobasal medium (34°C-36°C; maintained with an in-line heater SH-27B, Harvard Apparatus) and were allowed to equilibrate for 10 minutes before an imaging protocol began. Single *z*-axis images were captured for both 340- and 380-nm excitation wavelengths at 0.5 Hz using an inverted Olympus IX71 microscope, an Olympus oil immersion objective × 40 and a Charge-coupled device (CCD) camera coupled to Slidebook software (2 × 2 pixel binning). Baseline fluorescence was established during 5 minutes of perfusion before a + 10 mmol/L KCl stimulus was washed in. Fura-2 340/380 ratios were then calculated before and during + 10 mmol/L KCl wash-in, using fluorescence intensities averaged across neuronal cell body regions of interest and normalized to background fluorescence.

## 3 Results and discussion

To elevate neuronal activity in dissociated hippocampal cultures, we used a well-characterized manipulation involving chronic depolarization with moderately increased extracellular potassium. A + 10 mmol/L KCl stimulus is known to depolarize hippocampal neurons by ~ 15 mV, producing a significant and sustained elevation in intracellular calcium levels that has been previously associated with a range of structural and functional plastic neuronal changes (Evans et al., 2013, Grubb and Burrone, 2010a). Although this is certainly not a strictly physiological manipulation, it is an appropriate and useful one—plastic changes induced by this stimulus have also been produced in response to more naturalistic temporal patterns of spike activity (Evans et al., 2013, Grubb and Burrone, 2010a, Mistry et al., 2011). It is also considerably less extreme than the chronic depolarizing stimuli commonly used to initiate activity-dependent signaling in cultured neurons, which often reach 50 mmol/L KCl or higher (Redmond et al., 2002, Flavell et al., 2008). Here, we used a moderate (+ 10 mmol/L) KCl stimulus for 24 hours from 10 DIV, alongside a + 10 mmol/L NaCl treatment as a control for osmotic changes, to investigate the DNAm changes brought about by sustained depolarization. We first used RRBS (Gu et al., 2011) to quantify DNAm in DNA extracted from replicate control (NaCl) and depolarized (KCl) hippocampal samples (Supplementary Table 1). Raw RRBS reads underwent stringent quality control, were aligned to the rat reference genome (rn4), and filtered so that all potentially methylated cytosines (at both CpG and non-CpG sites) included in subsequent analyses had a minimum read depth of 10 reads in all samples (see Materials and methods). In total 1,552,276 CpG sites with a median minimum read depth of 33 (SD, 59.06) and 5,889,893 non-CpG sites with a median minimum read depth of 27 (SD, 51.61) were included in our analysis. DNA methylation across all CpG sites showed the expected bimodal distribution with most sites showing low levels of DNAm (Supplementary Fig. 2). As expected, DNAm was elevated on the X-chromosome, and sites on the mitochondrial genome were predominantly unmethylated (Supplementary Fig. 3). Also as expected, non-CpG methylation was found to be much lower across the genome (Supplementary Fig. 4). CpG sites located intragenically were characterized by higher average levels of DNAm compared to those located in exons, transcription start sites (TSSs), or transcription end sites (TESs) (Supplementary Fig. 5), consistent with previous reports (Guo et al., 2011a), with the highest levels of DNAm observed in intergenic regions.

**Fig. 1:**
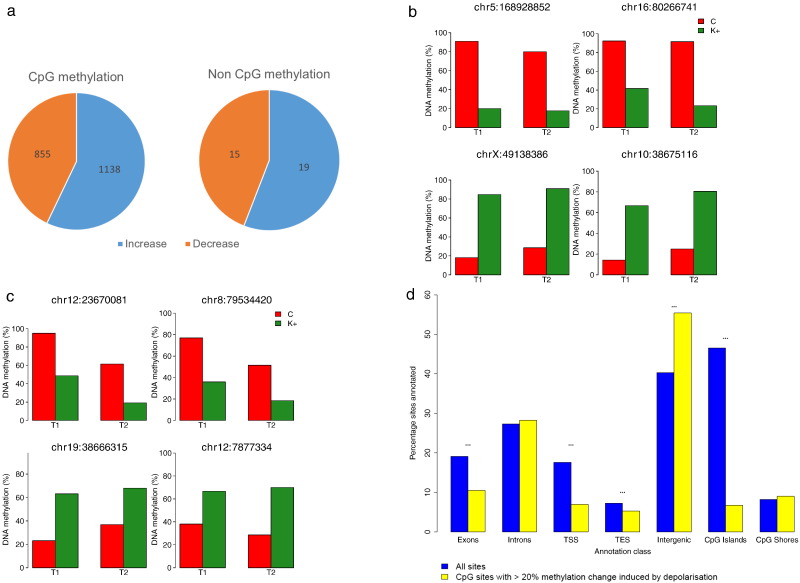
Depolarization induces DNAm changes in dissociated rat hippocampal neurons. (a) Consistent large (≥ 20%) changes in DNAm were observed at 1993 CpG sites and 34 non-CpG sites. In both CpG and non-CpG sites, the majority of these changes were characterized by increases in DNAm. Shown are the top-ranked CpG (b) and non-CpG (c) sites showing increased and decreased DNAm after depolarization. (d) Depolarization-induced DNAm changes are significantly enriched (indicated by **) at intergenic CpG sites but depleted at CpG sites located in exons, TSSs, and TESs.

A total of 1993 CpG sites (0.12% of those included in the analysis) were found to exhibit consistent changes in DNAm after depolarization (defined as an average change of ≥ 20% in the same direction across both replicates), with a bias (57.1%) toward sites showing an increase in DNAm after stimulation (Fig. 1a and Supplementary Table 2). Although non-CpG sites were generally unmethylated and invariable in response to depolarization, 34 sites (5.775E-4% of those included in the analysis) showed consistent changes in DNAm (Supplementary Table 4). Again, there was an excess of sites (55.9%) characterized by an increase in DNAm. The top-ranked CpG and non-CpG sites becoming hypermethylated and hypomethylated after depolarization are shown in Fig. 1b and c, respectively. Depolarization-induced changes were found to be significantly overrepresented in intergenic regions (*P* = 1.08E-42) and depleted in exons (*P* = 1.76E-22), TSSs (*P* = 1.73E-34), TESs (*P* = 7.31E-4), and CpG islands (*P* = 9.73E-278), an observation consistent with previous reports (Guo et al., 2011a) (Fig. 1d). Gene ontology (GO) analysis of annotated loci within 10 kb of these depolarization-induced dynamic CpG sites highlights a significant enrichment of loci implicated in neuronal function (Table 1 and Supplementary Table 3), including the “regulation of the glutamate receptor signaling pathway” (odds ratio, 4.74; *P* = 3.42E-5), “excitory synapse activity” (odds ratio, 3.87; *P* = 1.98E-4), and the “regulation of nerve impulse transmission” (odds ratio, 3.78; *P* = 2.40E-4). Although there were too few dynamic non-CpG sites to formally test their genomic distribution or perform GO analyses, the majority (58.8%) were also found to be intergenic in location.

**Fig. 2:**
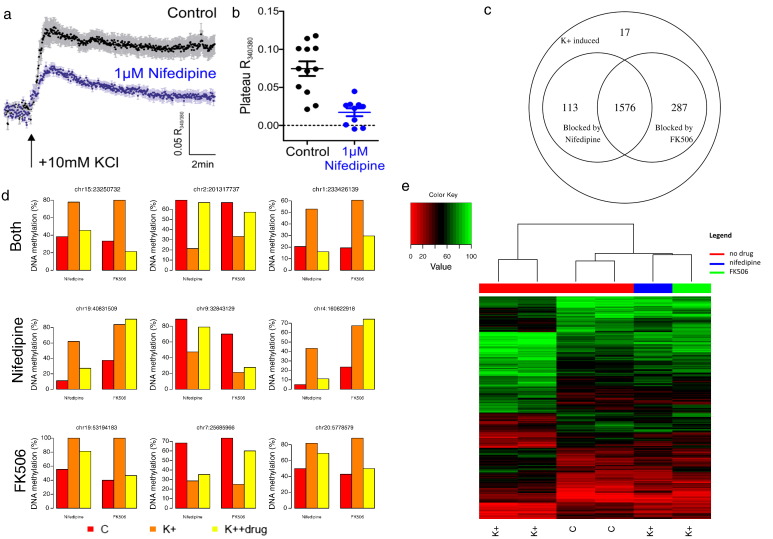
Activity induced changes in DNAm are blocked by L-type calcium-channel and calcineurin blocking drugs. L-type channels carry the bulk of chronic calcium entry produced by + 10 mmol/L KCl depolarisation. (a) Fura-2 ratiometric calcium imaging of dissociated hippocampal neuron responses to elevated external potassium. Dark dots show mean, light bars show SEM of the 340-nm/380-nm fluorescence intensity ratio at each time point. (b) Plateau depolarization-induced 340-nm/380-nm fluorescence intensity ratios for control (n = 12) and nifedipine-treated (n = 10) neurons. Bars show mean ± SEM (control, 0.075 ± 0.01; nifedipine, 0.017 ± 0.005). (c) Many of the activity-induced DNAm changes are blocked by either nifedipine or FK506, with a large proportion of the changes blocked by both drugs. (d) Examples of CpG sites where large DNAm changes induced by neuronal activation are blocked by both nifedipine and FK506 (top row), nifedipine only (second row), and FK506 only (bottom row). (e) Stimulated cells treated with nifedipine or FK506 are epigenetically more similar to unstimulated cells across these blocked sites.

Previous work has shown that several forms of activity-dependent neuronal plasticity depend on calcium signaling via L-type Ca_V_1 calcium channels and the downstream activation of multiple calcium-dependent kinase and phosphatase signaling pathways (Deisseroth et al., 2003, Greer and Greenberg, 2008, Wheeler et al., 2012). This includes activity-dependent relocation of the axon initial segment, which can be induced by chronic depolarization of hippocampal neurons with + 10 mmol/L KCl and which depends on signaling via L-type calcium channels and the calcium-activated phosphatase calcineurin (Evans et al., 2013, Grubb and Burrone, 2010a). To investigate whether these pathways are associated with the activity-induced DNAm changes described above, we used a pharmacological approach. First, hippocampal cultures were treated with + 10 mmol/L NaCl or KCl in the presence of 1 μmol/L nifedipine, which selectively blocks L-type calcium channels (Lee et al., 2006). We used calcium imaging with the ratiometric indicator fura-2 to show that nifedipine blocks > 75% of the sustained calcium entry produced by our + 10 mmol/L KCl stimulus (Fig. 2a and b), indicating that the calcium signaling evoked by membrane depolarization largely occurs through L-type channels. To assess the contribution of calcineurin signaling to activity-induced changes, we also treated our cells with + 10 mmol/L NaCl or KCl in the presence of 1 μmol/L FK-506, a potent calcineurin antagonist. Previous data have shown that, at this concentration, FK-506 does not alter either the membrane depolarization or the sustained calcium entry evoked by a chronic + 10 mmol/L KCl stimulus but does entirely block downstream calcineurin-dependent signaling (Evans et al., 2013).

Both pharmacological manipulations had notable effects on the extent and magnitude of depolarization-induced DNAm changes. The addition of nifedipine blocked 47.0% of the consistent CpG methylation changes, whereas FK506 blocked 41.4%, with 26.9% of CpG sites blocked by both drugs (Fig. 2c). In addition, many of the depolarization-induced DNAm changes at non-CpG sites were prevented by each drug, with ~ 44% being blocked by both. Specific examples of depolarization-induced changes in DNAm that were blocked by nifedipine and/or FK506 are shown in Fig. 2d. Hierarchical clustering analysis based on DNAm at these CpG sites highlighted that the drug-treated stimulated samples are more similar to unstimulated/untreated samples than stimulated/untreated samples (Fig. 2e).

To our knowledge this is the first evidence linking these particular signaling pathways to active alterations in DNAm. Given the crucial roles of both L-type Ca_V_1 channel and calcineurin signaling in multiple forms of neuronal plasticity (eg, Zeng et al., 2001, Deisseroth et al., 2003, Misonou et al., 2004, Greer and Greenberg, 2008, Schwartz et al., 2009, Schonewille et al., 2010, Wheeler et al., 2012, Evans et al., 2013), it is maybe not surprising that these pathways play a crucial role in activity-dependent DNAm. However, a previous study describing epigenetic responses after the direct manipulation of neuronal activity found a crucial dependence of these changes on the glutamatergic NMDA receptor (NMDAR) instead (Guo et al., 2011a). This difference could be explained if a proportion of the crucial NMDAR signaling were subsequently carried via voltage-gated Ca_V_1 activation and/or activation of calcium-dependent calcineurin. Alternatively, it is possible that the brief electroconvulsive stimulus used to induce those NMDAR-dependent DNAm changes acted via (at least partially) separate signaling pathways to the chronic 24-hour depolarization stimulus that we used here. Regardless, it is evident that a substantial proportion of depolarization-induced DNAm changes occur via L-type Ca_V_1 and/or calcineurin activation, which opens the question of the identity of their downstream effectors. One prime candidate is Gadd45b, crucially involved in active DNA demethylation in hippocampal neurons, whose expression—at least in dissociated culture—is regulated by depolarization in a Ca_V_1 channel-dependent manner (Ma et al., 2009). De novo DNAm, on the other hand, is largely mediated by DNMT enzymes (Guo et al., 2011a, Feng and Fan, 2009), but here, there are no explicit reported links to either Ca_V_1 channels or calcineurin. In fact, 1 cell culture study investigating the activity dependence of DNMT1 and DNMT3a found that depolarization-induced decreases in their expression in cortical cultures were not blocked by high concentrations of the Ca_V_1 antagonist nimodipine (Sharma et al., 2008). The increases in DNAm that we report here in response to chronic depolarization may, therefore, result from the action of different, as-yet unidentified, Ca_V_1 and/or calcineurin-dependent mechanisms.

There are several caveats to our study that should be addressed in future work. Pharmacological manipulations, for example, are often subject to issues of specificity, and although 1 μmol/L nifedipine is a reasonably selective antagonist of Ca_V_1 channels (Lee et al., 2006), FK506 inhibits calcineurin only indirectly and is known to have a range of intracellular targets (Kang et al., 2008). More caution is, therefore, required in interpreting our FK506 data, and future work may use genetic means of specifically manipulating calcineurin activity (Schwartz et al., 2009, Schonewille et al., 2010, Wu et al., 2010) for greater specificity. Future studies should also focus on manipulating electrical activity in a more physiological manner in defined cell types. Elevated extracellular potassium is an extremely useful and effective stimulus for inducing activity-dependent phenomena, some of which have been subsequently shown to be triggered by more subtle activity manipulations (Evans et al., 2013, Grubb and Burrone, 2010a). Moreover, the depolarizing stimulus that we used here is considerably milder than routinely used KCl concentrations in the field (Redmond et al., 2002, Flavell et al., 2008). However, it is still certainly not a strictly physiological manipulation. Furthermore, our dissociated cultures prepared from embryonic hippocampi are known to contain a wide variety of cell types, not least multiple different types of neuron (Evans et al., 2013, Williams et al., 2011). Fortunately, addressing both of these issues should be eminently possible in the near future using the conditional expression of optogenetic tools, coupled with the application of physiologically relevant photostimulation protocols (Evans et al., 2013, Grubb and Burrone, 2010b, Fenno et al., 2011). Finally, RRBS only interrogates a selected proportion of potentially methylated cytosines in the genome, focusing on CG-rich regions, and future analyses should use whole-genome sequencing approaches. We were also not able to distinguish between DNAm and its oxidized derivative, DNA hydroxymethylation, as standard bisulfite sequencing approaches do not distinguish between these (Huang et al., 2010). This is an important confounder given that the latter is known to be abundant and to have functional relevance in neural cells (Lister et al., 2013, Globisch et al., 2010, Khare et al., 2012).

## 4 Conclusion

To conclude, we used RRBS to test whether depolarization induced changes in DNAm in rat hippocampal cells. Our results confirm previous reports of widespread changes in DNAm associated with neural activity (Guo et al., 2011a). Consistent with previous findings, these changes were significantly enriched in intergenic regions and underrepresented in CpG-rich promoter regulatory regions. We also found a significant enrichment of changes in pathways associated with neuronal function and activity. Pharmacological blocking demonstrated that a surprisingly high proportion of these activity-induced changes are likely to be mediated by calcium entry through L-type Ca_V_1 channels and/or downstream signaling via the calcium-dependent phosphatase calcineurin.

## Author contributions

MSG and JM devised the study. ANC, MDE, MSG, and CCYW undertook laboratory experiments. EH performed data analysis. EH, MSG, and JM drafted the manuscript. All coauthors read and approved the final submission.

## Competing financial interests

None of the authors has any competing interests to declare.

## Figures and Tables

**Table 1:** Gene ontology terms associated with CpG sites showing consistent changes after depolarization.

Pathway	Ontology	GO:ID	Genes in pathway	Probes in pathway	Overlap pathway and test list	*P* value	FDR q value
Regulation of ion transmembrane transporter activity	BP	GO:0032412	37	4334	15	2.90E-5	0.0563
Regulation of glutamate receptor signaling pathway	BP	GO:1900449	17	2446	11	3.42E-5	0.0563
Regulation of α-amino-3-hydroxy-5-methyl-4-isoxazole propionate selective glutamate receptor activity	BP	GO:2000311	14	2050	10	3.96E-5	0.0563
Positive T-cell selection	BP	GO:0043368	11	1772	9	7.05E-5	0.0752
Excitatory synapse	CC	GO:0060076	20	2996	11	1.98E-4	0.142
Regulation of transmission of nerve impulse	BP	GO:0051969	26	3065	11	2.40E-4	0.142
Tissue homeostasis	BP	GO:0001894	56	4702	14	2.46E-4	0.142
Positive regulation of membrane potential	BP	GO:0045838	18	2167	9	3.08E-4	0.142
Wnt signaling pathway, planar cell polarity pathway	BP	GO:0060071	14	1724	8	3.20E-4	0.142
Thymic T-cell selection	BP	GO:0045061	18	2190	9	3.33E-4	0.142

Top 10 gene ontology terms that were overrepresented in the list of CpG sites that showed a consistent depolarization change.

Key: BP, biological process; CC, cellular component; MF, molecular function.
