# Supplementary material for: A role for CaV1 and calcineurin signaling in depolarization-induced changes in neuronal DNA methylation
Source: Neuroepigenetics. 2015 Jun 25;3:1–6. doi: 10.1016/j.nepig.2015.06.001 (PMC4659419; doi:10.1016/j.nepig.2015.06.001)
Supplement: Supplementary file 1 — Supplementary material [file mmc1.pdf]

Supplementary Figure 1:

Reduced representative bisulfite sequencing (RRBS) protocol produced high quality reads for all samples. The median quality score per base, across all samples are presented as boxplots, taken from FastQC output. Quality is measured by phred scores, where scores higher than 28 are indicated by the green box and those between 20 and 28 are indicated by the orange box. Across all bases, and all samples, the calls were of a high quality with a small probability of being incorrect.

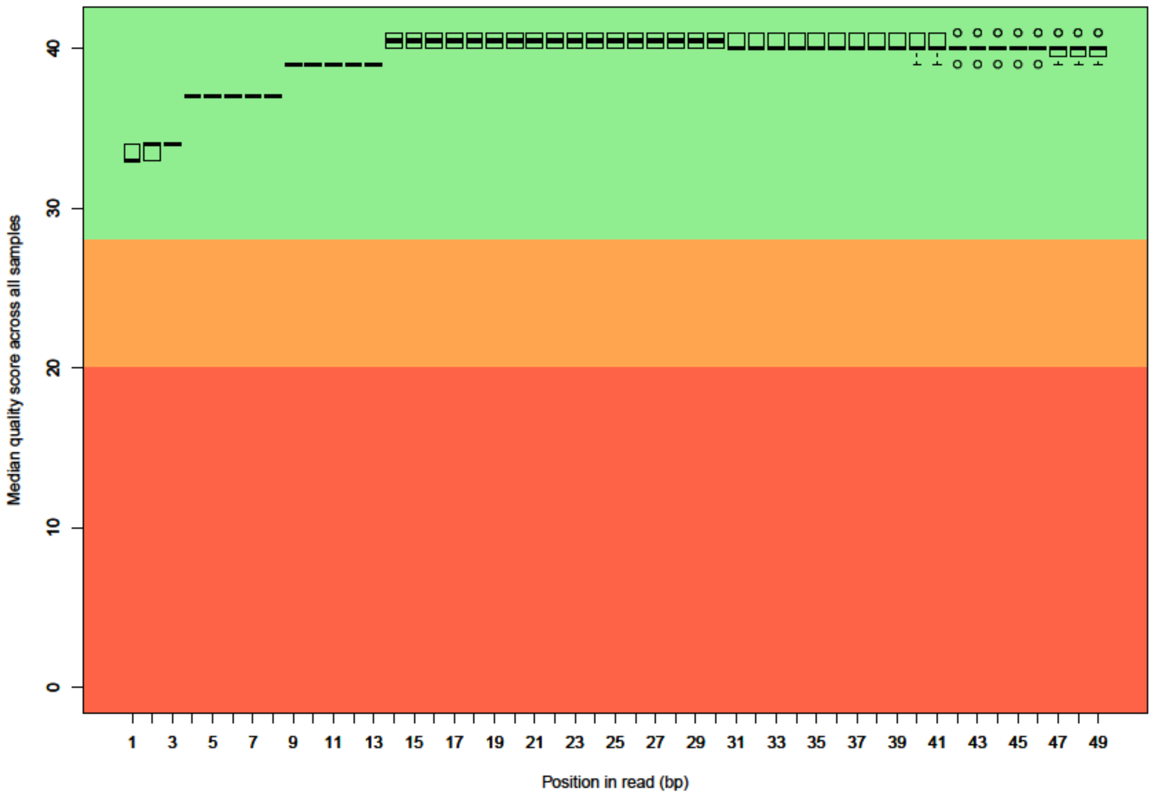

Supplementary Figure 2:

Distribution of median DNA methylation values at CpG sites across all samples in the set of high quality sites (≥ 10 reads in all samples) taken forward for analysis. A bimodal distribution, with peaks of hyper- and hypomethylation as previously described for the Rat genome.

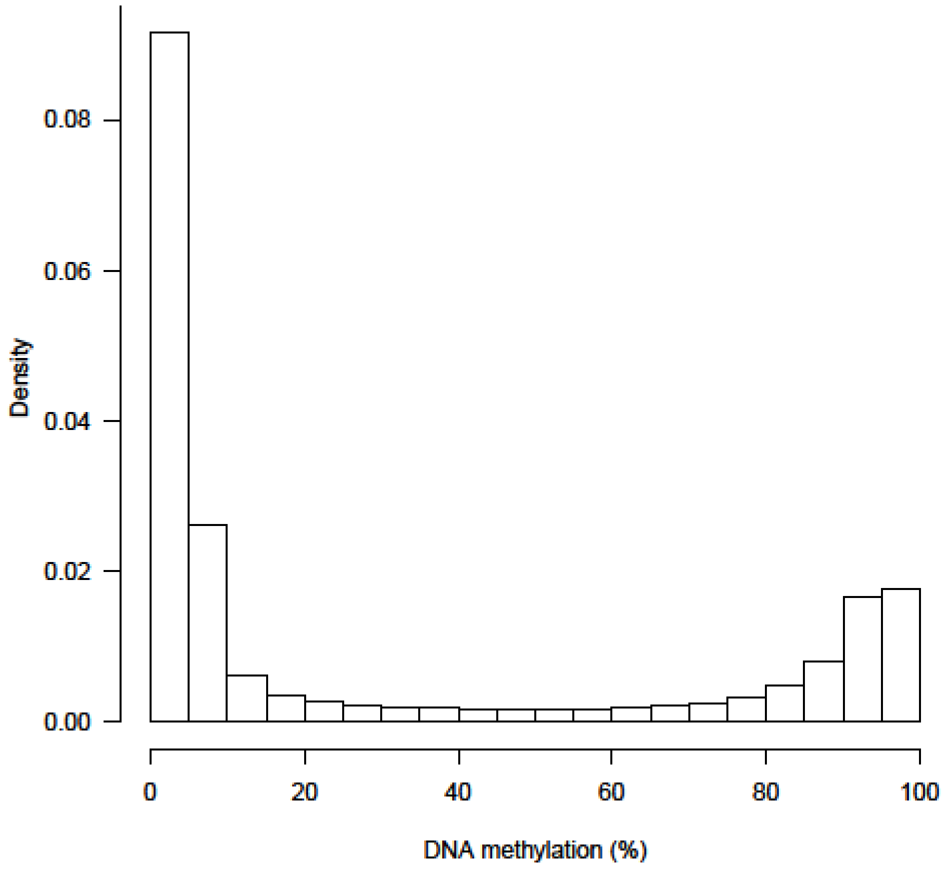

Supplementary Figure 3:

Boxplots illustrating the distribution of CpG methylation values across chromosomes. The distribution of CpG methylation is consistent across the autosomes while, as expected, the X-chromosome is relatively hypermethylated, and the mitochondrial genome is largely unmethylated.

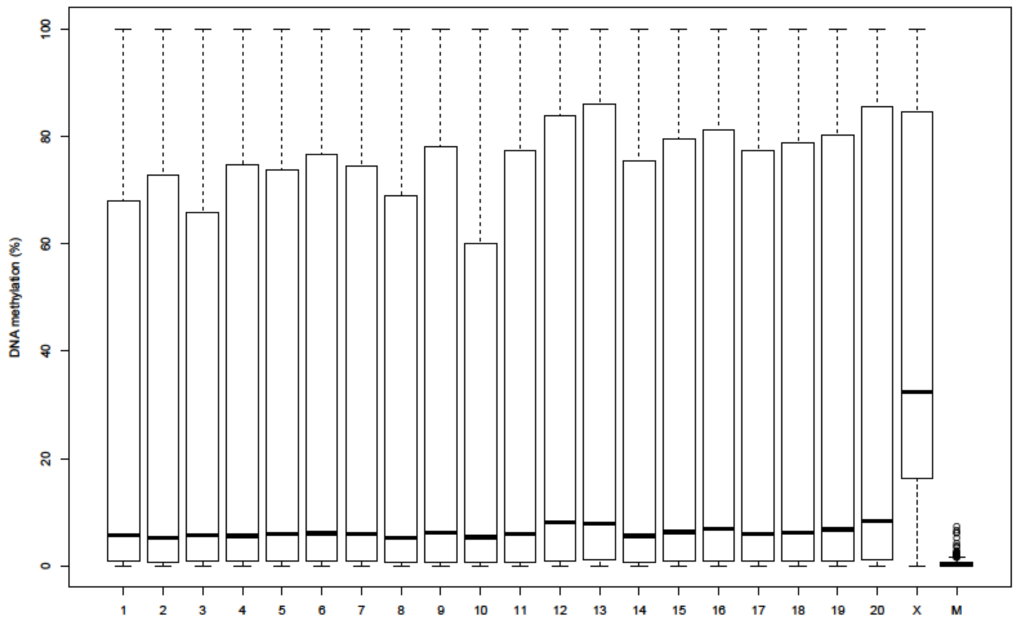

Supplementary Figure 4:

Distribution of DNA methylation values at non-CpG sites per sample in set of high quality sites (≥10 reads in all samples) taken forward for analysis. Y –axis is on a log scale, to show non-negligible amount of hypermethlation at these sites. DNA methylation is generally low, but there are a number of non-CpG sites with high levels of DNA methylation.

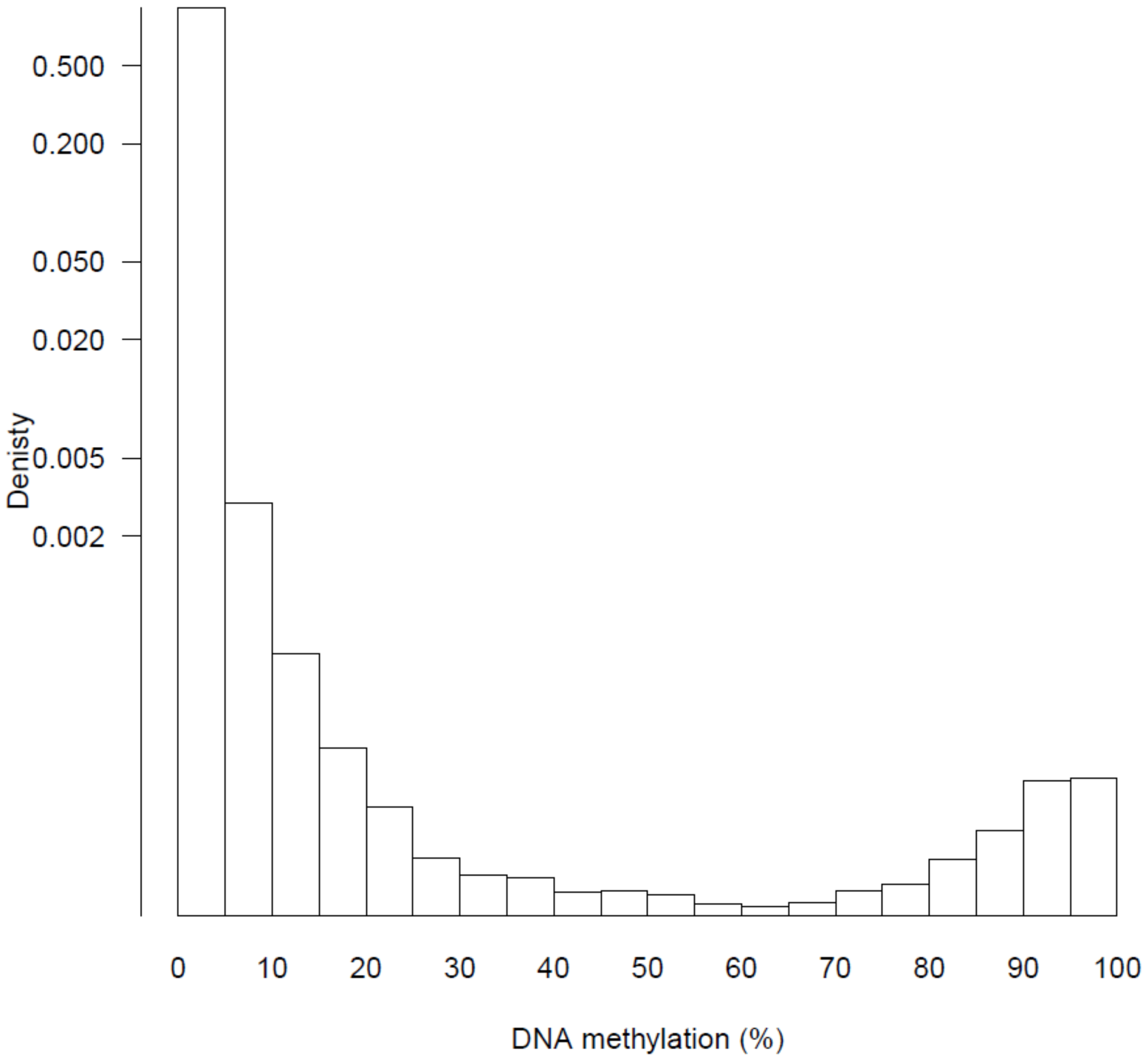

Supplementary Figure 5:

The distribution of CpG DNA methylation levels across genic features. DNA methylation is highest at intergenic CpG sites, with the largest proportion of hypermethylation, while the lowest levels are found in the Transcription Start Sites (TSS); intronic DNA methylation is on average higher than exonic CpG methylation. DNA methylation is higher in CpG shores compared to CpG islands. Abbreviations: TSS – Transcription Start Sites; TES – Transcription End Sites.

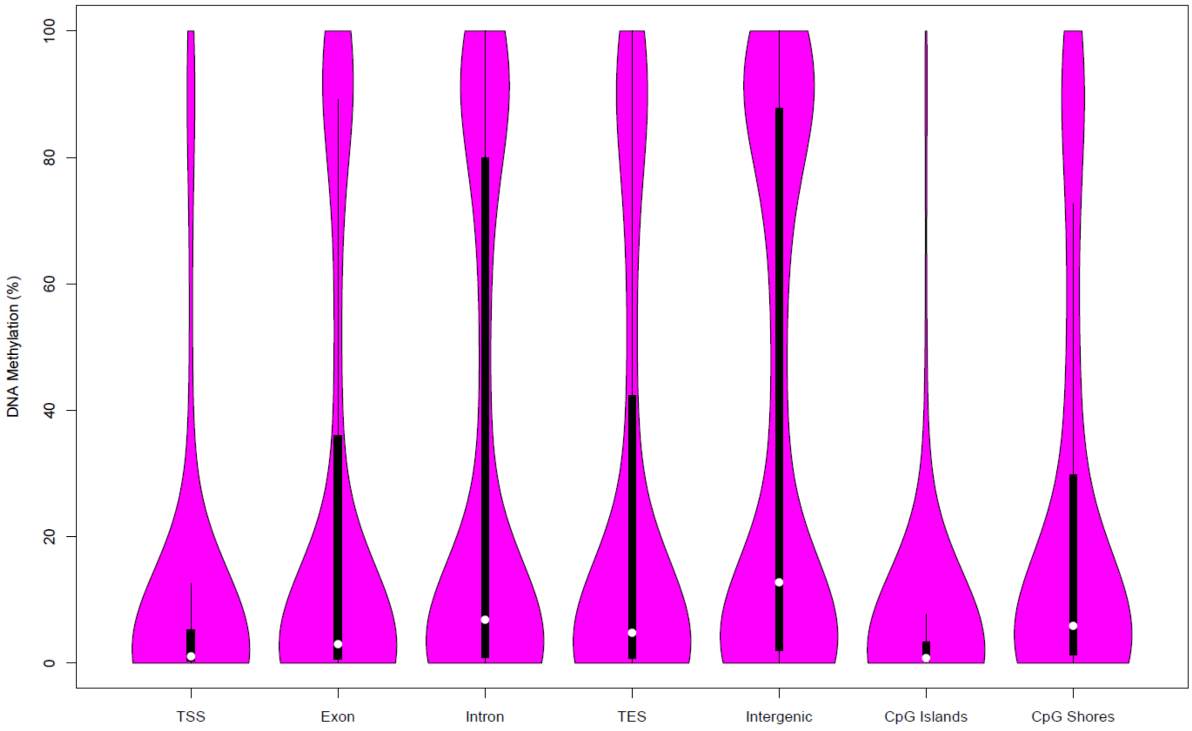

Supplementary Table 1: Sequencing data summary before and after alignment.

A) All reads

Sample ID	Total sequences read 1	Total sequences read 2	Total sequence pairs inputted to Bismark	Total paired end alignments with unique best hit	% Mapping efficiency	Total number of sites	Number of CpG sites	Number of non-CpG sites
**A3**	35756895	35756895	35756162	22315842	62.41	64794742	6601946	58192796
**A4**	41787572	41787572	41785381	25532731	61.1	66961309	6923178	60038131
**A5**	38039622	38039622	38036413	19964280	52.49	78090227	7188281	70901946
**A6**	43408861	43408861	43406542	24236767	55.84	130761273	10820038	119941235
**A7**	38661554	38661554	38660172	21496955	55.6	77684366	7662222	70022144
**A8**	43320387	43320387	43319154	26904552	62.11	63585580	6665084	56920496
**A9**	33881738	33881738	33880524	20961444	61.87	53552996	5757675	47795321
**A10**	44419399	44419399	44417722	25476620	57.36	57110353	6185808	50924545

B) Reads mapped to CpG sites

Sample ID	Number of sites	Minimum read depth	1st quartile read depth	Mean read depth	Median read depth	3rd quartile read depth	Maximum read depth	% Read depth ≥ 5	% Read depth ≥ 10	Sites with read depth ≥ 5	Sites with read depth ≥ 10
**A3**	6601946	1	1	16.77	1	16	12364	34.72	29.35	2292480	1937682
**A4**	6923178	1	1	18.54	1	13	16882	33.6	27.76	2325957	1921844
**A5**	7188281	1	1	12.73	1	13	11029	32.36	27.37	2325929	1967336
**A6**	10820038	1	1	9.64	1	3	12595	20.66	16.86	2235438	1824433
**A7**	7662222	1	1	13.65	1	9	18020	29.64	24.24	2270815	1857002
**A8**	6665084	1	1	20.15	1	15	30156	33.55	28.23	2236104	1881849
**A9**	5757675	1	1	17.82	2	20	19153	38.64	32.25	2224681	1856956
**A10**	6185808	1	1	20.45	2	19	26064	36.56	30.68	2261628	1897659

C) Reads mapped to non-CpG sites

Sample ID	Number of sites	Minimum read depth	1st quartile read depth	Mean read depth	Median read depth	3rd quartile read depth	Maximum read depth	% Read depth ≥ 5	% Read depth ≥ 10	Sites with read depth ≥ 5	Sites with read depth ≥ 10
**A3**	58192796	1	1	7.24	1	2	12374	17.40	13.79	10124866	8024412
**A4**	60038131	1	1	7.77	1	2	16870	16.82	13.03	10100269	7823633
**A5**	70901946	1	1	5.39	1	2	11460	14.64	11.71	10379639	8302256
**A6**	119941235	1	1	3.72	1	1	12604	8.03	6.09	9627068	7299567
**A7**	70022144	1	1	5.70	1	2	18064	13.98	10.66	9792368	7467503
**A8**	56920496	1	1	8.39	1	2	30126	16.95	13.38	9646345	7618137
**A9**	47795321	1	1	7.91	1	2	19163	20.22	15.75	9664771	7528251
**A10**	50924545	1	1	8.86	1	2	26047	19.27	15.14	9811542	7709799

Supplementary Table 2: Depolarization changes induced by K+ at CpG sites. A consistent change > 20% in DNA methylation replicates was identified at 1,993 CpG sites. A proportion of these changes were subsequently blocked in the presence of either Nifedipine or FK506. Site location is from rn4. Gene annotation gives the rat genome database id; if a site was intergenic the nearest gene within 10kb was identified.

Chromosome	BP	Median read depth	Depolarization change pair 1	Depolarization change pair 2	Blocked by Nifedipine	Blocked by FK506	Gene annotation	CpG island annotation
chr5	168928852	24.5	-70.91	-62.35	No	No	Intron:1562703	
chrX	49138386	29.5	66.43	62.34	No	No		
chr16	80266741	18.5	-50.64	-68.33	No	No	Intron:620224 | TSS:2318753	
chr7	32357772	21	-57.35	-55.65	No	No		
chr10	38675116	19	52.38	55.56	No	No		
chr7	134413288	54	-51.67	-54.76	No	No		
chr7	114205631	19.5	-47.62	-62.34	No	No	Exon:621649	
chr20	10284955	26.5	49.44	55.74	No	No	Intron:1307063 | 69407	
chr9	11461858	37.5	-48.41	-57.02	No	No		
chr1	53333052	20.5	47.73	56.83	No	No		
chr4	69874645	26.5	55.83	48.86	No	No		
chr19	53194183	15.5	44.44	60.00	No	Yes	Intron:1303000	
chr8	116787665	18	-52.27	-48.18	No	No	Intron:1593239	
chr17	14991383	47.5	48.89	49.57	No	No	Intron:708480	
chr1	82297061	23.5	-42.86	-72.64	No	No	Intron:631336	
chr5	162347031	30	43.60	59.62	No	No		
chr1	53333051	24.5	-60.91	-46.70	No	No		
chr5	127789481	22	-48.05	-49.19	No	No	Intron:1563440	
chr12	39991248	32	-65.28	-46.17	No	No	Closest gene:1562674	
chr9	99124729	28.5	-53.85	-46.87	No	No		
chr15	103756183	35	43.41	54.07	No	No		

chr18	30597711	35	-56.66	-45.80	No	No	Exon:1592968 | Intron:1590761 | 1587259 | 1598664 | TSS:1311949	CpG Shore
chr19	40831509	24.5	50.96	46.50	Yes	No	Closest gene:1307000	
chr20	18375008	27.5	-42.11	-59.36	No	No	Intron:1307063	
chr7	118032152	22	43.64	51.92	No	No	Intron:628898	
chr13	46867378	52	-52.38	-45.60	No	No	Exon:620226	
chr17	13132021	37.5	55.56	45.21	No	No		
chr14	10210268	30.5	42.40	50.26	No	No		
chr15	39224067	21	-56.02	-43.85	No	No		
chr14	80672504	17.5	50.00	44.84	No	No		
chr12	26853519	25.5	40.48	57.23	No	No		
chr19	53913751	35	48.33	45.18	No	No		
chr5	58488457	33.5	51.57	43.43	No	No		
chr9	87804656	21.5	52.53	42.97	No	No		
chr13	76806403	31	-57.89	-42.47	No	No	Intron:1565820	
chr9	32843129	19.5	-41.52	-48.57	Yes	No		
chr12	284386	16	46.67	43.68	No	Yes		
chr12	7925402	16	50.00	42.33	No	No	Intron:2621	
chr8	88550765	13	40.56	48.00	Yes	No	Closest gene:2323003	
chr6	11631084	19.5	62.50	40.85	No	No		
chr5	148656776	38	-40.64	-46.75	No	No		CpG:_42
chr1	181470668	14	45.45	42.86	Yes	No		
chr8	115776856	18.5	43.33	43.64	No	No	Exon:1563130	
chr7	25685966	19.5	-39.61	-48.33	No	Yes	Exon:1310252	
chr5	155699181	23	46.49	41.98	No	No	Exon:708543	
chr3	164098656	25	38.24	51.67	No	No	Intron:1308255	
chr4	145733452	29.5	57.01	40.03	No	No		
chr3	54710712	30	52.84	40.32	No	No	Intron:621886 | 1564504	
chr1	235061384	28.5	37.86	50.95	No	No	Exon:1310026	CpG Shore

chr17	69706879	28	-37.04	-58.06	No	No		
chr15	23250732	12.5	39.32	46.67	Yes	Yes	TSS:1311455	CpG:_58
chr7	137128178	34.5	-39.94	-45.73	No	No	Exon:1590570	
chr6	136002058	30.5	-46.32	-41.15	Yes	No	Intron:1304633	CpG Shore
chr9	9800700	36	38.99	46.24	No	No	Exon:1562008 | TES:1559787	
chr17	42920196	48	39.68	45.28	No	No		
chr3	156190265	24	-36.81	-53.61	No	No	Intron:2321	
chr10	19557768	13.5	-55.83	-39.16	No	No	Intron:1564189	
chr5	167772524	30	38.06	46.83	No	No	Exon:629475	
chr12	40057400	59.5	-38.79	-45.41	No	No	Intron:1562674	
chr20	21918874	34.5	39.28	44.19	No	No	Intron:1307063	
chr17	16778306	25	49.38	39.37	No	No		
chr20	9718564	32.5	37.18	48.06	No	No	Intron:1307063 | 1308587	
chr1	253286059	22	-35.71	-67.74	No	No	Exon:1562316	
chr15	57225539	28	-35.71	-67.56	No	No		
chr2	38258255	34.5	-39.76	-43.52	No	No		
chr16	30969175	14	40.11	42.21	Yes	No	Intron:1560014	
chr14	85387898	31.5	64.12	38.15	No	No	Intron:1359269 | TES:1559774	CpG:_48
chr13	24915565	27	44.00	39.94	Yes	No		
chr15	105952033	30.5	45.75	39.19	No	No		
chr14	10123769	23.5	-35.29	-58.04	No	No		
chr2	45384892	19	37.50	45.40	Yes	No		
chr4	160622918	35.5	38.33	43.71	Yes	No	Closest gene:621153	
chr14	43680060	10.5	37.30	45.45	Yes	Yes	TES:1560582	CpG Shore
chr20	10817935	31.5	36.34	47.76	No	No	Intron:1307063 | 1311263	
chrX	131483370	37.5	34.83	60.26	No	No		
chr8	66884114	18	-36.67	-45.09	No	No	Intron:1311711	
chr5	168567686	39.5	-34.34	-62.04	No	No	Intron:1562703	
chr18	78958405	24.5	-45.71	-38.17	No	No		

chr1	137938142	26	35.75	47.00	No	No	Closest gene:620705	
chr12	42297702	48.5	40.74	39.74	No	No	Intron:1593434	
chr4	168527270	30.5	-38.96	-40.79	No	No		
chr5	107074202	34.5	41.15	39.27	No	No	Intron:620940	
chr6	137948590	24	-38.24	-41.59	No	No	Closest gene:1310583	
chr12	34428346	30.5	37.79	41.98	No	No	Intron:1564948	
chr3	169419954	48.5	-36.88	-43.49	No	No		
chr18	77165300	32	40.59	39.51	No	No		
chr4	62677818	36	-49.64	-37.10	No	No	TES:1359715	
chr3	160071375	25	44.44	37.95	No	No		
chr17	32972020	15.5	-42.86	-38.21	No	No		
chr16	74643905	29.5	42.11	38.35	No	No	Exon:2180	
chr8	63188547	32	-37.47	-41.38	No	No	Intron:1309134	CpG Shore
chr14	104918512	20.5	33.33	65.86	No	No		
chr5	168567764	22	34.13	48.93	No	No	Intron:1562703	
chr19	51824771	28	44.22	37.07	No	No		
chr1	181296565	45	35.32	44.44	No	No	Intron:3396	
chrX	92554872	31.5	-61.60	-35.56	No	No		
chr4	80520211	53.5	-43.85	-36.64	No	No	TSS:1587253 | TES:1310001	CpG Shore
chr12	40057399	63.5	36.38	41.33	No	No	Intron:1562674	
chr7	66772488	23	40.17	37.89	No	No		
chr9	8510763	39.5	-46.67	-36.18	No	No	Intron:1311386	CpG:_188
chr10	26370371	17.5	43.54	36.52	No	No		
chr6	42903566	36.5	-36.84	-40.09	No	No		
chr2	244225902	17.5	-33.41	-48.33	No	No		
chr1	87118728	27	36.47	40.83	No	No	Exon:1583737	CpG Shore
chr3	7014240	60.5	-37.50	-39.46	No	No	Intron:621222 | 1564504	
chr14	85883713	43.5	56.59	35.29	No	No	Intron:620789	
chr5	170614819	32.5	-37.14	-39.85	No	No		CpG Shore

chr7	124168979	29	-37.25	-39.42	No	No		
chr1	207485311	16.5	-36.67	-40.00	No	No	Intron:621792	
chr13	84029716	34.5	-37.14	-39.41	No	No	Intron:1308213	
chr1	246483614	34.5	37.55	38.85	No	No	Exon:2319516	
chr16	50575274	24	-36.67	-39.86	Yes	No		CpG:_17
chr10	97479097	41	-38.65	-37.69	No	No		
chr3	122800556	45.5	-34.44	-42.86	No	No	Intron:3344	CpG Shore
chr2	27523469	22	43.33	35.71	No	Yes		
chr3	58925888	30	34.81	41.82	No	Yes	Intron:621793 | 1564504	
chr12	35818875	23	37.50	38.35	Yes	No		CpG Shore
chr6	98899030	21.5	42.86	35.71	No	No	Exon:708531	
chr19	35189810	19.5	32.27	51.85	No	No	TSS:1310790	CpG:_38
chr4	159314169	48	-31.85	-58.33	Yes	No	Intron:1565101	
chr14	10648473	17.5	-35.90	-39.66	Yes	No		
chr13	68195629	31.5	-32.90	-46.44	No	No	Intron:621053	
chr17	25386141	18	39.85	36.47	No	No		
chr8	115873663	42	-32.69	-46.92	No	No	Intron:1309232	
chr15	61117166	15.5	50.00	34.62	Yes	No		
chr4	126577031	41.5	33.51	43.33	No	No	Exon:1306723	
chr7	122344473	44.5	-78.41	-34.10	No	No		
chr20	29084961	31.5	-35.51	-39.77	Yes	No	Intron:1307063 | TSS:1304964	
chr16	70943925	45	32.74	46.28	No	No		
chr7	12493624	19	37.30	37.73	Yes	No	TES:1311781	
chr3	143399604	25	-33.33	-42.92	No	No	Exon:1359224	CpG:_22
chr1	181591813	30	35.52	39.02	No	No	Intron:628803	
chr15	105971651	27	31.99	48.14	No	No		
chr8	61738805	28.5	-35.87	-38.42	No	No	Exon:1305192	
chr2	247359506	11.5	34.51	40.00	No	No		
chr15	57233196	15	31.67	49.88	No	No		

chr5	160107853	50.5	36.59	37.52	No	No		
chr1	42023603	50	-36.27	-37.68	No	No		
chr19	53451949	27	-34.52	-39.59	No	No	Intron:1307466	
chr12	22465480	37	52.38	33.62	No	No	Intron:631346	
chr4	168527269	28	34.62	39.29	No	No		
chr10	13018010	69	32.36	44.25	No	No	Intron:1309385	
chr8	44141283	41.5	-33.52	-40.52	No	No	Closest gene:1310357	
chr8	45253503	21	39.35	35.45	No	Yes		
chr9	88897887	32	32.09	44.70	No	No	Intron:1309244	
chr1	209768148	41.5	-36.36	-36.98	Yes	No	Exon:1565152	
chr7	63222469	43.5	38.00	35.87	No	No		
chr3	158425239	17.5	-31.58	-46.75	No	No		
chr1	228233091	39	-30.77	-55.56	No	No	Intron:1307969	
chr20	5778579	13.5	31.82	45.38	Yes	Yes	Intron:1307063 | TSS:1564450	
chr17	82980542	22.5	38.42	35.48	No	No		
chr15	9321460	32.5	-35.71	-37.35	Yes	No	Intron:3858	
chr2	201317737	14.5	-47.54	-33.33	Yes	Yes	Intron:1311319	
chr13	68195628	24	31.10	49.75	No	No	Intron:621053	
chr3	3610359	46.5	-57.78	-33.22	No	No	Exon:620238	CpG:_19
chr14	106751927	34	30.54	59.09	No	No		
chr19	52682236	16	33.99	38.89	Yes	No	Exon:1303257	
chr18	60229614	20	38.85	35.06	No	No		
chr8	46817642	32.5	-31.14	-48.13	No	No		
chr3	5970174	44	31.41	46.17	No	No	Intron:1564504 | TSS:1306074	
chr16	33101738	29	38.24	35.05	Yes	No		
chr1	233426139	29	32.35	41.25	Yes	Yes	TES:1306525	
chr5	120054732	17.5	-45.39	-33.33	Yes	No		
chr18	61340268	30.5	30.70	49.42	No	No	Intron:735047	CpG:_48
chr5	145504918	20	31.57	43.79	No	Yes	TES:1308876	CpG Shore

chr9	8604765	24.5	30.36	54.07	No	No		
chr7	30626000	52	-31.45	-44.00	Yes	No	Intron:1564046	
chr17	15350851	43	33.06	39.50	No	No	Exon:1308082 | TSS:1309112 | TES:628624	
chr10	7381642	13	-58.33	-32.58	No	No		
chr10	49934833	26.5	38.82	34.55	No	No		
chr3	54185853	20	-40.90	-33.76	Yes	Yes	Intron:1564504	
chr1	246483613	44	-33.50	-38.41	No	No	Exon:2319516	
chr6	40750256	54	40.00	34.02	No	No		
chr6	102916268	33.5	-31.48	-43.36	No	No		
chr12	38604825	14	37.25	35.15	No	No		
chr1	123106935	35	-36.19	-35.85	Yes	No	Intron:1585883	
chr17	74691180	54	-37.24	-35.12	No	No		
chr19	53451950	32.5	46.04	33.11	No	No	Intron:1307466	
chr5	170522692	48.5	30.29	52.01	No	No		
chr6	126647302	27	31.32	43.29	Yes	No	Intron:1305849	
chr9	74522111	49	31.87	41.19	No	No	Intron:1591035	
chr8	76195861	44	-33.75	-37.57	No	No	Exon:1304623	
chr4	118569639	13	30.77	45.71	Yes	No		
chr8	124075534	24.5	-42.86	-33.21	Yes	No	Intron:1561649	
chr5	114347411	36.5	31.82	41.03	No	No		
chr9	89659528	29	-29.85	-55.69	No	No		
chr1	230281573	20	32.58	38.76	No	No		
chr11	77502424	27.5	32.07	39.81	No	No	Intron:1306226	
chr10	90780355	45	-32.76	-38.46	No	No	Exon:1310723	
chr12	21233949	16.5	-47.00	-32.35	No	Yes	Intron:620618	
chr17	23810045	34.5	45.85	32.42	No	No		
chr1	110586144	12	-40.15	-33.33	No	Yes		
chr10	82080609	21.5	34.51	36.04	Yes	Yes		
chr1	186188064	50	-36.48	-34.63	No	No	Intron:70932	

chr6	2439473	21.5	32.17	38.89	Yes	Yes	TSS:1559836	
chr1	260526435	13	39.68	33.33	Yes	No		
chr19	11114354	20.5	41.38	32.86	Yes	Yes	Intron:3686	
chr16	19295831	19.5	-30.55	-43.70	No	No	Closest gene:621165	
chr6	107834519	13.5	54.55	31.67	Yes	Yes	Intron:1311176 | TES:620107	
chr3	160655485	31.5	32.87	37.50	No	No		
chr13	81567058	29.5	34.85	35.40	No	No	Intron:2305	
chr19	35799301	30.5	-40.38	-32.90	No	No	Intron:3687	
chrX	92511174	45.5	-33.57	-36.33	No	No	Exon:1564806	CpG:_21
chr15	18612104	12	34.62	35.45	Yes	No		
chr8	104014138	26	-29.44	-50.70	No	No		
chr3	157716864	50	-36.05	-34.22	No	No	Exon:1306534	
chr2	221655420	44.5	-31.67	-38.79	No	No		
chr6	132507641	47	30.38	42.19	No	No	Intron:1309550	
chr5	170566984	36	-48.48	-31.48	No	No		
chr15	46377644	15	-30.56	-41.21	Yes	Yes	Intron:2517	
chr17	14248339	39.5	40.91	32.28	No	No		
chr2	228699330	34.5	-33.33	-36.00	No	No		
chr5	170554217	19.5	-30.34	-41.84	No	No		
chr6	143820484	27	-33.33	-35.80	No	No	Intron:61904	
chr1	245651006	36	-33.33	-35.76	No	No		
chr6	27198084	17.5	46.06	31.49	No	No		
chr1	262195872	51	41.21	32.05	No	No	Intron:1583621	
chr1	208285673	50.5	-34.49	-34.77	No	No	TES:1305529	
chr7	59174313	27	68.75	30.93	No	No		
chr10	57497814	31	29.59	44.84	Yes	Yes	Exon:2353 | TSS:1597577 | TES:1359135	CpG:_23
chr6	27389121	73.5	38.85	32.45	No	No		
chr20	7643395	32	-55.94	-30.95	No	No	Intron:1307063 | 1304760	CpG:_28
chr3	88556830	25.5	30.07	41.64	No	No	Intron:1310282 | 1564504	

chr11	10273262	30	30.26	40.95	No	No		
chr18	59578903	45.5	29.05	49.44	No	No		
chr15	53256745	16.5	35.83	33.46	Yes	No	Intron:1304736	
chr13	105857029	30.5	-42.08	-31.65	No	No		
chr5	170566990	36	-43.00	-31.48	No	No		
chr10	47621963	17	-28.89	-50.49	Yes	No		
chr14	10123770	20.5	29.15	47.31	No	No		
chr3	38868844	42	-38.52	-32.35	No	No	Intron:1564504	CpG Shore
chr1	185363863	24.5	-39.35	-32.14	Yes	No	Exon:628713	CpG Shore
chr17	86960955	55	-29.24	-45.85	No	No		
chr14	100822353	22.5	-32.35	-36.18	Yes	No		
chr20	42134463	24	31.48	37.50	Yes	No	Intron:1307063	
chr8	106210462	16.5	28.57	69.00	No	No		
chr9	9800701	29	-31.25	-37.95	Yes	No	Exon:1562008 | TES:1559787	
chr6	129343784	25.5	43.48	31.26	Yes	No		
chr19	50128029	20.5	50.00	30.86	No	No	Intron:1305498	CpG Shore
chr8	125326601	40.5	-29.67	-42.47	Yes	No	Intron:727731	
chr11	36603254	16.5	32.31	35.96	Yes	No	Intron:1306727	
chr12	11669438	22	36.35	33.16	No	Yes		CpG:_262
chr20	43390705	30.5	28.44	69.48	No	No	Intron:1307063	
chr1	246568064	22	28.51	57.70	Yes	No		
chr5	160454456	15	28.75	48.40	Yes	Yes		
chr7	59509929	43	50.00	30.71	No	No		
chr12	7223665	36	29.83	40.95	No	No	Closest gene:3716	
chr6	129255347	34	-41.03	-31.36	No	No		
chr10	66609824	20.5	-36.19	-32.89	No	No		
chr1	23855590	13	-30.56	-38.46	Yes	Yes		
chr7	14575002	25.5	35.90	32.96	No	No		
chr16	19295830	24	36.91	32.39	No	No	Closest gene:621165	

chrX	61078044	15	28.79	46.67	No	No		CpG Shore
chr7	116797185	18	30.95	37.50	No	No		
chr3	88556829	25.5	-31.54	-36.38	Yes	No	Intron:1310282 | 1564504	
chr13	25994325	14	28.57	47.92	No	No		
chr7	99569543	21	29.63	40.95	Yes	No		
chrX	91985134	17.5	30.00	39.71	No	No	Intron:1559832	CpG:_38
chr6	53666542	28.5	35.98	32.79	No	No		
chr1	191052384	46.5	42.23	31.03	No	No		
chrX	67518085	14.5	-28.33	-54.55	No	Yes		
chr2	224824420	20	28.41	51.11	No	No	Exon:1305834	
chr2	132600652	17.5	-28.57	-46.71	No	Yes		
chr8	62670903	17	31.10	36.81	Yes	Yes	TES:71065	
chr4	186707084	24	-29.87	-39.80	No	No		
chr4	142769320	61.5	-39.67	-31.28	No	No		
chr7	11085942	16	29.68	40.00	Yes	No	Exon:1307185 | TES:1308653	CpG:_70
chr9	45140894	14.5	36.67	32.14	Yes	Yes	Intron:1561071	
chr5	156484432	17.5	33.85	33.33	No	No	Intron:1309908	
chr8	70919543	17	-34.76	-33.09	No	No		
chr12	32634279	18.5	-28.85	-43.64	Yes	Yes	Intron:1310293	
chr2	31687585	43.5	34.90	32.88	No	No		
chr17	14248340	47	-29.19	-41.39	No	No		
chr4	8936584	52.5	-30.92	-36.46	No	No		
chr20	14174685	37	42.46	30.68	No	No	Intron:1307063 | TSS:2717	CpG Shore
chr15	36582681	37	47.70	30.18	No	No	Intron:1595926	CpG Shore
chr10	108973359	16	33.75	33.33	No	No		
chr12	21146356	59.5	-29.16	-41.29	No	No		
chr6	144416545	16	28.24	48.33	No	Yes	Intron:61904	
chr16	80490339	54.5	-30.27	-37.32	Yes	No		
chr2	256004580	30.5	-41.65	-30.62	No	No		

chr1	262595359	18	36.84	31.62	Yes	No	TES:1302979	
chr6	53878434	23	30.77	36.23	No	No		
chr1	79826152	36	28.31	46.34	No	No	TES:1309409	
chr2	21482915	39.5	-27.67	-64.73	No	No		
chr1	203848965	16	30.00	37.50	No	No	TSS:1303323	
chr19	15416127	13	-37.56	-31.23	No	Yes		
chr8	61738804	39.5	40.79	30.40	No	No	Exon:1305192	
chr7	123700042	39.5	-27.78	-50.27	No	No		
chr5	170567035	36	-32.11	-33.95	No	No		
chr4	168527320	34	28.13	45.65	Yes	No		
chr1	77566434	17	30.89	35.42	Yes	Yes		
chr10	95154741	29	28.49	42.21	No	No	Intron:1309285	
chr10	72868477	69.5	34.52	32.02	No	No		
chr5	58488522	42.5	-31.42	-34.41	No	No		
chr8	106210461	31.5	-36.46	-31.18	No	No		
chr8	114871743	34.5	36.54	31.11	No	No	Intron:1565187	
chr14	27391843	18	-28.57	-40.44	Yes	Yes		
chr20	12282861	44.5	54.54	29.39	No	No	Intron:1307063	
chr13	50105680	47	32.41	33.23	No	No		
chr20	45906533	24.5	31.59	33.75	No	No		
chr3	3610304	75.5	31.41	33.94	No	No	Exon:620238	CpG:_19
chr17	79465554	13.5	-35.80	-31.17	Yes	Yes		
chr8	66697052	30.5	-38.80	-30.18	No	No		CpG Shore
chr1	22028495	34.5	51.77	29.27	No	No		
chr8	109714475	43	-31.59	-33.39	No	No	Intron:1308844	
chr9	104133842	23	-38.82	-30.09	No	No		
chr4	62401164	21.5	-27.65	-45.45	Yes	No	TSS:1565367	
chr6	11631007	47.5	-30.59	-34.56	Yes	No		
chr5	170614820	48	27.50	46.36	No	No		CpG Shore

chr20	4093455	42.5	37.59	30.31	No	No	Exon:1303059 | Intron:1307063 | TSS:1359601 | TES:1303267	CpG Shore
chr6	132245013	34	-28.65	-38.66	No	No		
chr9	90536659	66.5	30.90	34.04	No	No	Intron:1359514	
chr19	20454688	49.5	29.17	37.13	No	No		
chr1	263130827	30.5	-27.80	-42.50	Yes	No	TSS:1306140	CpG:_44
chr18	2280301	40	-29.47	-36.19	No	No		
chr10	13003039	12	-38.46	-30.00	Yes	Yes	TSS:1310795 | 1561157	
chr3	167147456	30.5	-27.59	-44.02	Yes	No		
chr1	128497612	22	50.00	29.00	No	No		
chr10	92275746	27	-36.11	-30.64	No	No	TSS:1310903 | 1308404	CpG Shore
chr7	126341354	24.5	31.11	33.39	No	Yes		
chr18	31573873	29.5	-29.16	-36.65	No	No		
chr1	16762989	24	-27.27	-45.98	No	No		
chr1	8220153	13.5	28.83	37.18	No	No		
chr2	246100179	36.5	28.13	39.52	No	No		
chr13	49392438	57	-47.67	-28.89	No	No	TSS:1310950	
chr9	90001470	14.5	-27.78	-40.91	No	Yes	Exon:1308704	
chr4	154492067	16	27.81	40.51	Yes	No	TSS:3556	CpG Shore
chr11	85046852	27	27.47	42.90	No	No		
chrX	138565979	25.5	31.90	32.31	No	No		CpG:_143
chr2	177002591	24.5	31.50	32.67	Yes	No		
chr7	127742942	19	42.00	29.17	Yes	No		
chr11	32860756	23.5	32.78	31.65	No	No		
chr1	220601250	14	31.37	32.79	No	Yes	Intron:1311350	
chr10	7214610	21	-36.36	-30.04	Yes	No	TSS:1560593	CpG Shore
chr2	196986364	25	-29.76	-34.67	Yes	Yes	Exon:1311184	
chr7	103571194	21	-35.64	-30.33	No	Yes	Intron:69222	
chr1	168641348	17.5	-27.89	-39.56	No	Yes	Intron:1309828	

chr10	98855666	46	26.94	47.46	No	No		
chr17	93459184	93	27.92	39.22	No	No		
chr15	56798354	91.5	27.54	41.02	No	No	TES:1585590	
chr11	32748151	37.5	-41.07	-29.10	No	No		
chr4	145733451	33.5	-28.17	-38.18	No	No		
chr19	26665118	32.5	34.66	30.46	No	No		
chr20	7149667	24	-37.62	-29.61	No	No	Intron:1307063 | 1306685	
chr3	15323895	52.5	30.77	32.94	No	No	Intron:1564504	CpG Shore
chr10	45804686	44	-32.38	-31.52	No	No	Intron:1304611	
chrX	118037875	13	-29.55	-34.52	No	No		
chr10	30892012	15	-32.38	-31.47	Yes	No		
chr10	91710972	43.5	-30.00	-33.65	No	No	Exon:71012	CpG:_167
chr1	2763579	40	-34.62	-30.36	No	No		
chr1	163494507	42	40.60	29.01	No	No	Exon:621296 | TES:1309878	
chr7	105923162	27	26.67	49.65	No	No		
chr10	35691718	17	30.25	33.33	Yes	No	Intron:1310147	
chr5	154343947	17	-49.47	-28.47	No	Yes	Intron:1308320	CpG Shore
chr7	10165828	77.5	-27.44	-40.06	No	No	Exon:1310863	CpG:_38
chr8	92001439	15	28.94	35.29	No	No	Closest gene:1311282	
chr1	80252140	21.5	29.85	33.62	Yes	Yes		
chr1	203835811	22	39.39	29.02	No	No	Exon:727892	CpG Shore
chr13	29195508	48	35.83	29.86	No	No	Intron:1559812	
chr1	203810553	44.5	-35.86	-29.85	No	No	Closest gene:621503	
chr15	87664269	23	26.32	60.73	No	No	Intron:1305102	
chr10	11246930	19.5	-54.40	-28.13	No	No		
chr9	52520506	13	-30.42	-32.73	No	Yes	Exon:621798	CpG:_21
chr6	130726404	14	-35.71	-29.76	Yes	Yes		
chr18	61392867	23	28.92	34.82	No	No	Intron:735047	
chr13	46867377	77.5	29.27	34.09	No	No	Exon:620226	

chr1	208285674	53.5	27.18	40.69	No	No	TES:1305529	
chr20	5934227	18	-27.78	-37.88	Yes	No	Exon:1594842	
chr17	26371577	14.5	29.52	33.49	Yes	No		
chr9	12829429	39.5	26.67	45.97	No	No	Intron:69198	
chr8	47088685	18.5	-27.17	-40.48	Yes	Yes	Exon:1307477 | TSS:1308802 | TES:1305953	
chr16	77124652	14.5	-27.27	-40.00	Yes	Yes		
chr2	35552643	15.5	-27.98	-36.67	Yes	Yes		
chr10	91743942	39	-28.79	-34.62	No	No		
chr2	177002590	19	-26.19	-53.07	Yes	No		
chr2	180522673	42	31.42	31.36	Yes	No		
chr5	149030798	49.5	35.29	29.58	No	No	Intron:1309431	CpG:_27
chr18	80690978	23	31.12	31.59	No	No		
chr10	13018009	54	-26.63	-44.47	No	No	Intron:1309385	
chr4	145733739	24	39.13	28.59	No	No		
chr6	130073297	56.5	33.29	30.28	No	No		
chr19	32252045	27.5	26.92	41.30	No	No	Closest gene:1588859	
chr5	154758797	58.5	30.83	31.69	No	No	Exon:1308048 | TSS:620210	
chrX	99285270	17	-28.41	-35.29	Yes	No		
chr8	12251572	19.5	33.11	30.22	Yes	Yes		
chr4	117849078	13.5	-27.24	-39.16	Yes	Yes	Intron:621062	
chrX	69100305	38.5	-33.25	-30.14	No	No		
chr6	136002027	30.5	-28.68	-34.47	Yes	No	Intron:1304633	CpG Shore
chr1	42023608	50	-46.57	-27.90	No	No		
chr3	17539126	29	28.53	34.95	Yes	Yes	Intron:1564504 | TSS:1309368	
chr10	15759568	43.5	29.54	33.15	No	No		
chr20	11364511	20	-30.18	-32.17	Yes	Yes	Intron:1307063 | TES:1304860	
chr9	85279483	18	-34.67	-29.61	No	No		
chr16	19226938	28	29.78	32.77	No	No	Closest gene:2321151	
chr13	70228431	28.5	52.31	27.68	No	No	Closest gene:1306554	

chr7	52082476	52	-27.90	-36.12	No	No		
chr5	171631061	31	43.52	28.00	No	Yes		
chr4	6102674	55.5	33.10	30.00	No	No		
chr20	4108826	25.5	-32.87	-30.11	Yes	Yes	Exon:620005 | TES:1359601	
chr5	138602990	38.5	35.78	29.17	No	No	Exon:1306378 | TSS:68414	
chr15	186648	34.5	26.24	46.49	No	No	Exon:1308753	
chr10	10232324	19.5	31.50	30.88	Yes	No		
chr13	87966935	62	-29.80	-32.41	No	No	Intron:1309442	
chr10	72079827	13	33.12	30.00	Yes	Yes		CpG Shore
chrX	89376271	16	-28.07	-35.29	Yes	No	Exon:621119 | TES:1585896	
chr3	144307023	13	-37.88	-28.57	Yes	No	Intron:1565613	
chr6	130268230	30	42.56	27.88	No	No		
chrX	35589184	17	27.06	38.79	Yes	No		CpG:_102
chr10	37376368	22.5	-26.67	-40.95	No	Yes	Intron:1309152	
chr17	92611428	44.5	-29.28	-33.08	No	Yes		
chr2	219820158	27.5	-29.86	-32.22	No	No	Exon:1564779	
chr2	190152066	18.5	25.86	53.64	No	No	Exon:1309478 | TSS:1359334	CpG Shore
chr17	85600860	23	-25.77	-57.82	Yes	No	Intron:1561482	
chr18	31573993	25.5	26.09	46.75	No	No		
chr12	22400722	31	-27.67	-36.07	No	No	Intron:620305	
chr12	25966632	18.5	26.63	41.27	No	Yes		CpG Shore
chr5	144821295	56.5	31.59	30.58	No	No		
chr7	140324532	16	35.71	28.94	No	No	Intron:1359576	
chr3	158789032	80.5	40.97	27.93	No	Yes	Intron:1309093	
chr5	138602991	27.5	-51.00	-27.38	No	No	Exon:1306378 | TSS:68414	
chr5	85780070	22	-28.01	-35.00	No	No		
chr3	160655486	53.5	-26.07	-46.37	No	No		
chr18	53120111	19.5	-33.25	-29.82	Yes	Yes	Intron:1565632	
chr19	18802873	20	29.57	32.27	No	Yes		

chr3	11246977	17.5	-27.08	-38.00	No	No	Intron:1564504	
chr6	10862457	47	-26.00	-46.90	No	No	Intron:1306211	
chr3	152727732	11.5	26.92	38.64	No	No	Intron:1309653	
chr8	76195862	48	44.79	27.58	No	No	Exon:1304623	
chr7	135966564	21	-27.78	-35.32	No	No	TES:727911	CpG Shore
chr13	66764668	36.5	35.86	28.68	No	No		
chr8	61738845	60	-26.76	-38.85	No	No	Exon:1305192	
chr5	150663602	20	-32.14	-30.00	Yes	Yes	Intron:620782	
chr9	60511424	55.5	-29.17	-32.50	No	No	Intron:1584992	
chr1	29703066	12	27.27	36.67	No	No		
chr10	106375518	28	25.45	64.34	No	No		
chr8	115008008	32.5	-35.06	-28.79	No	No	TSS:708524	
chr12	35668963	26.5	-28.47	-33.33	No	No	TSS:1564245	
chr2	201557841	24	29.44	32.00	Yes	No		
chr8	21046588	16.5	27.27	36.15	No	Yes	Intron:1597171	
chr3	159488888	18.5	-27.59	-35.21	Yes	Yes		
chr10	13898353	15.5	34.52	28.85	No	No	Intron:620380	
chr10	83302540	81.5	-27.62	-34.94	No	No	Closest gene:1309090	
chr1	120716397	32	27.43	35.42	Yes	No	Intron:1308000	
chr10	15687744	23	27.65	34.78	No	Yes	Exon:2907	
chr2	26157183	27	-38.42	-27.80	No	No	Closest gene:2321734	
chr2	174854036	18.5	37.14	28.07	No	Yes		CpG Shore
chr9	38074405	23.5	27.59	34.87	Yes	Yes		
chr20	14154675	39.5	32.41	29.50	No	No	Intron:1307063 | 2717	CpG Shore
chr11	70720473	28.5	25.93	43.30	No	No	Exon:1308155	
chr5	60105257	16.5	-28.77	-32.48	No	Yes	TSS:620851	CpG Shore
chr8	83912030	13.5	36.19	28.33	Yes	Yes		
chr16	60704850	39.5	30.71	30.41	No	No		
chr5	137623080	25.5	27.50	34.87	No	Yes		

chr9	8635238	66.5	-33.27	-29.16	No	No	Intron:1560271	
chr17	15545326	37.5	-25.76	-44.01	No	No	Exon:1308068	
chrX	94522231	13	-27.86	-33.77	Yes	No		
chr5	165951324	47	-31.77	-29.71	No	Yes		
chr8	125011206	54	-25.69	-44.15	Yes	No	Closest gene:1304776	
chr14	87914442	24	29.43	31.31	Yes	No	Intron:1309318	
chr6	132342238	11.5	-25.57	-45.45	Yes	Yes	Intron:1311128	
chr1	97601474	23.5	-29.76	-30.99	Yes	No	TSS:1305749	
chr5	58488523	32	25.09	57.38	No	No		
chr4	156993985	24.5	25.25	49.09	No	No		
chr10	49934834	30	-27.47	-34.42	No	No		
chr10	13898352	21.5	-56.67	-26.74	No	No	Intron:620380	
chr1	188301478	48	33.33	28.79	No	No		
chr17	95324874	26.5	26.16	39.42	No	Yes	Intron:1305841	CpG Shore
chr19	39158604	17.5	27.08	35.29	Yes	No		
chr6	144894548	18.5	-25.92	-40.70	Yes	Yes		
chr8	120579211	47	26.07	39.74	No	No	Exon:1565145	
chrX	89376281	16	-27.07	-35.29	Yes	Yes	Exon:621119 | TES:1585896	
chr5	160250190	28	25.91	40.55	Yes	No		
chr2	227885668	32.5	25.00	72.34	No	No		
chr17	18669207	20	25.00	70.22	No	No		
chr2	140414540	24	28.00	32.97	No	No	Exon:1586346	
chr4	122301995	49.5	-26.79	-35.94	No	No	TSS:1561981	
chr10	89642608	88	28.57	31.97	No	No	Exon:1307242 | TSS:1310093	
chr13	50105620	71.5	-25.98	-39.49	No	No		
chr6	102853501	23	-27.97	-32.87	Yes	Yes		
chr17	15062350	85	-44.93	-26.84	No	No	Intron:1309214	
chrX	89919394	14.5	-25.00	-54.73	No	No	Intron:1564117	CpG:_98
chr20	22296557	37.5	-25.00	-53.44	No	No	Intron:1307063	

chr18	77165299	33	-25.00	-53.16	No	No		
chrX	92511173	34	-31.62	-29.23	No	No	Exon:1564806	CpG:_21
chr8	92944197	53	28.36	31.99	No	No		
chr4	106226895	64	-26.25	-37.48	No	No	Closest gene:2323800	
chr15	41840592	16	25.77	39.49	No	Yes	Intron:1593344 | TSS:2323891	
chr8	48380031	63	25.72	39.77	No	No	Intron:2173	
chr17	19178356	80	-28.85	-31.13	No	No		
chr13	42764272	17	31.15	29.37	Yes	No	Intron:1306938	
chr6	98936246	71.5	-24.89	-60.73	No	No	Closest gene:620829	
chr2	182057334	26.5	33.94	28.04	Yes	No	Intron:1583215	CpG Shore
chr18	61583045	20	28.75	31.15	Yes	Yes	Intron:1564431	
chr1	33892328	54	-30.23	-29.85	No	No		
chr1	163494508	59	-25.56	-40.25	No	No	Exon:621296 | TES:1309878	
chr20	6099221	46	28.64	31.14	No	No	Exon:1307264	
chr17	9681550	17.5	-26.32	-36.36	Yes	No	Intron:1311629	
chr12	7814526	20.5	29.22	30.48	No	No		
chr20	6223165	22	24.84	54.17	No	No	Exon:1309183	
chr7	138408963	13.5	50.20	26.37	No	Yes		CpG:_27
chrX	38725139	29	26.92	34.17	No	No		
chr2	43930032	15.5	-37.50	-27.12	No	No		
chr7	75677136	18.5	26.37	35.79	No	Yes		
chr1	175045332	19	37.22	27.18	No	Yes		
chr19	44129744	46	30.00	29.82	Yes	Yes		
chr10	92962215	40.5	-30.36	-29.48	No	No	Intron:3972	CpG:_71
chr4	177339457	15.5	-28.79	-30.77	Yes	Yes		
chr17	16569473	15.5	-25.00	-45.45	Yes	Yes		
chr4	9936050	23	25.64	38.95	No	No		
chr10	101679951	14.5	26.97	33.84	No	Yes		
chr3	52692388	15.5	31.33	28.89	No	Yes	Intron:1564504 | TSS:1308499	CpG Shore

chr17	27984418	16	-45.83	-26.47	No	Yes		
chr8	72976718	21	-28.95	-30.62	No	Yes		
chr6	66562850	12	41.07	26.67	Yes	No		
chr2	247085144	27	27.49	32.56	Yes	No		
chr14	9564591	46	25.57	38.78	No	No		
chr8	127580523	22.5	-25.00	-46.67	No	No		
chr6	133008772	21.5	29.46	30.00	Yes	Yes	Intron:621150	
chr12	32089295	46.5	-31.44	-28.67	No	Yes	Intron:1566191	
chr6	25588010	19.5	-25.76	-37.54	Yes	No		
chr6	47899729	103.5	28.53	30.92	No	No	Exon:1560694	CpG Shore
chr16	72334225	13.5	32.97	27.88	No	Yes		
chr12	15396413	16	38.39	26.70	No	Yes	Exon:1307875	
chr20	7194790	31	25.00	42.34	No	No	Intron:1307063 | 1591066	
chr5	168928853	31	55.56	26.07	No	No	Intron:1562703	
chr6	24897452	30	31.35	28.57	Yes	Yes		
chr20	38466118	31.5	-24.64	-48.69	No	No	Intron:1307063	
chr4	172275577	17.5	32.86	27.84	Yes	No		
chr11	82749615	17.5	26.92	33.33	No	No		
chr1	78480077	12	24.55	51.05	No	No	TSS:1308393	CpG Shore
chr2	154236029	23	37.79	26.71	No	Yes		
chr17	14075495	70	-25.65	-37.16	No	No	TES:620049	
chr12	13636824	34.5	-26.07	-35.69	No	No		
chr1	201648228	14.5	30.00	29.25	Yes	Yes	Intron:1560911	CpG Shore
chr1	13958626	30.5	-26.19	-35.24	No	No		
chr19	37335202	62	-24.50	-50.92	No	No	Intron:1310091	
chr20	49101915	51	47.06	26.11	No	No	Intron:620841	
chr4	186707078	24	-25.11	-39.80	No	No		
chr7	21416443	47	-28.18	-30.86	No	No		
chr14	86119028	12.5	25.00	41.43	Yes	Yes		

chr2	83535438	28	37.19	26.77	No	No	Intron:1591367	
chr1	185455112	31	25.91	36.03	Yes	No	Exon:620802 | TES:1305613	
chr20	4931535	13	-36.36	-26.92	Yes	Yes	Exon:2373	
chr10	97479098	81.5	25.37	38.23	No	No		
chr18	71659647	46	-24.43	-52.49	No	No		
chr2	176821702	19.5	-31.37	-28.42	Yes	Yes		
chr2	240858505	33	-35.24	-27.11	No	No	TES:2319336	
chr13	105083869	64	24.60	46.00	No	No		
chr2	114102673	42.5	-25.53	-36.96	No	No	Intron:1307225	
chr7	10917069	75.5	39.32	26.45	No	No	Exon:1308256	CpG Shore
chr1	67551774	66	24.90	42.37	No	No	TES:1565545	CpG:_159
chr11	72495552	39.5	-33.33	-27.37	No	No		
chr10	5495064	25.5	26.94	32.74	Yes	No		
chr11	69969361	22	-38.57	-26.52	No	No	TSS:1305957	CpG Shore
chr8	111021473	24	29.63	29.23	Yes	No	Exon:1564060	
chr6	29370826	18	30.00	28.95	No	Yes		
chr6	61498339	17.5	31.28	28.26	Yes	Yes		
chr1	124867786	19	25.00	40.00	Yes	No		
chr8	46274067	15.5	-49.02	-25.88	Yes	No		
chr7	127156153	36	-59.17	-25.72	No	No		
chr7	30099343	15	-24.56	-45.24	Yes	Yes	Closest gene:1589470	
chr8	63188546	42.5	24.88	41.78	No	No	Intron:1309134	CpG Shore
chr20	10284954	43	-27.27	-31.76	No	No	Intron:1307063 | 69407	
chr8	69459134	37	-25.00	-39.71	No	No	TSS:1306243	CpG Shore
chr1	34833626	17	-25.00	-39.68	No	No		
chr1	3324916	11.5	-28.33	-30.30	No	No		
chr5	137629313	12.5	-26.57	-33.33	Yes	No		
chr2	250650103	28.5	35.71	26.76	No	No		
chr16	81522338	25	-35.87	-26.70	No	No	Intron:1308688	

chr3	3610360	70.5	32.41	27.51	No	No	Exon:620238	CpG:_19
chr11	79246746	20.5	-36.36	-26.67	Yes	Yes		
chr4	5886471	16.5	28.24	30.26	Yes	Yes		
chr3	26265704	39.5	-31.09	-28.15	No	No	Intron:1564504	
chr1	184540251	40	-26.30	-33.75	No	No	Intron:1307760	
chr20	4793976	45	-25.24	-37.20	No	No	Exon:3817 | Intron:1307063 | TSS:3426	
chr1	125211144	37.5	26.67	32.69	Yes	No		CpG:_31
chr18	80651498	23	38.10	26.36	No	No		
chr3	161072170	14.5	26.92	32.23	No	No		
chr9	99124665	38	24.12	53.00	No	No		
chr9	84780413	19	25.00	38.89	Yes	No	Closest gene:2318569	
chr4	6055984	23.5	-40.48	-26.07	No	No		
chr9	100839713	16.5	25.68	35.23	No	No		
chr7	12861468	19.5	32.20	27.45	No	No	Closest gene:1307282	
chrX	113430006	26.5	24.18	47.96	Yes	No		
chr9	71281121	23.5	26.60	32.83	No	No		
chr10	84544431	12	-25.00	-38.33	No	No	Exon:1311794 | TES:1306182	
chr4	52701178	13	-30.00	-28.57	No	No		
chr19	11458480	21.5	-25.34	-36.14	Yes	Yes	Exon:1311551	
chr10	89199689	16.5	24.52	42.31	Yes	No	Exon:1359182	
chr15	39860488	66	25.99	33.96	No	No	Exon:1560876	
chr3	11237023	15	-26.71	-32.14	No	Yes	Intron:1564504	
chr8	113720647	22	24.00	50.51	Yes	No	Intron:621443	
chr9	89419516	57.5	29.00	29.20	No	No		
chr2	252494482	18.5	26.80	31.91	Yes	Yes		
chr2	252494505	18.5	26.80	31.91	Yes	Yes		
chr1	99313124	13.5	26.15	33.33	No	No	Intron:619778	
chr8	115412433	23	-31.56	-27.47	No	No	Intron:1565653	CpG Shore
chr1	189974357	15.5	-25.49	-35.00	No	Yes	Intron:1303004	

chr3	115688094	21	-30.00	-28.28	No	Yes	TSS:628774 | TES:1306814	CpG Shore
chr10	104596378	28	24.03	46.28	Yes	No		
chr1	154176487	12.5	25.40	35.15	No	Yes	TES:1304658	
chr5	50441283	23.5	-26.48	-32.33	Yes	No	Intron:2369	
chr17	14634384	18.5	29.77	28.41	Yes	Yes		
chr8	91025230	19.5	-32.38	-27.12	No	Yes	TES:2323631	
chr4	157568576	17	27.06	31.11	Yes	Yes		
chr8	69459135	40	24.17	43.72	Yes	No	TSS:1306243	CpG Shore
chr1	180796207	29.5	30.00	28.13	No	No	Intron:1566058	
chr13	95197222	21	50.45	25.25	Yes	Yes		
chr17	21919012	44.5	29.26	28.59	Yes	No		
chr6	143725933	20.5	24.89	37.86	Yes	No	Intron:1308406	
chr9	90279648	48	29.86	28.21	Yes	No	Intron:1359548	CpG Shore
chr15	14184250	26	26.36	32.30	Yes	Yes		
chr5	148656781	38	-29.31	-28.57	No	No		CpG:_42
chr12	42568251	75	-28.44	-29.36	No	No		
chr12	19654844	17.5	-27.73	-30.00	No	Yes	Exon:1310152	CpG:_19
chr3	166997358	17.5	-23.68	-58.55	No	No		
chr20	14369550	15.5	23.81	48.10	No	No	Intron:1307063	
chr6	129255348	32	26.67	31.47	No	No		
chr1	134705103	15.5	25.00	36.25	No	No		
chr14	72715532	19	32.05	27.08	No	No		
chr3	142447692	17	44.74	25.32	No	Yes	TES:1564796	CpG Shore
chr13	70228486	32.5	-31.20	-27.32	No	No	Closest gene:1306554	
chr4	117106225	22.5	-45.29	-25.24	No	No	Exon:3658	
chr6	137102593	46	23.66	52.39	Yes	No		
chr2	29028147	26.5	-40.00	-25.56	No	No		
chr4	160955178	18	23.81	45.72	No	No	Intron:1359093 | TSS:1308438 | TES:708344	CpG Shore
chr7	25247982	19.5	-36.22	-25.98	Yes	Yes	Intron:735102	

chr5	170817875	53	26.94	30.67	No	No	Intron:1587099	
chr3	154424138	73.5	31.17	27.26	No	No	TES:1305296	
chr13	12048381	18	27.98	29.33	No	Yes		
chr4	36132150	14	27.45	29.90	No	Yes		
chr7	22524273	19	24.31	38.89	Yes	No	TSS:1563365	
chr8	125064951	63.5	29.26	28.11	No	No		CpG:_40
chr17	71782063	28	-29.52	-27.86	No	No	Intron:1560155	
chr1	249564271	21	25.54	33.33	No	No	TES:1307644 | 1598329	CpG Shore
chr12	2988933	17	-23.53	-53.51	Yes	No	Intron:2917	
chr2	181415424	32.5	28.48	28.73	Yes	Yes	Exon:1305993 | TSS:1308413	
chr5	159291978	15.5	-28.28	-28.92	Yes	No		
chrX	26730011	15	-28.21	-28.94	Yes	No	Intron:621249	
chr9	59355620	73	-27.35	-29.90	No	No	Intron:2299	
chr1	42023632	51	-34.53	-26.14	No	No		
chr19	9895811	30.5	23.53	49.10	No	No		
chr14	85911339	19	-24.67	-36.46	Yes	No	TSS:620789	CpG Shore
chr5	160103058	43	29.89	27.55	No	No		
chrX	23120597	16	-26.57	-30.91	Yes	No		
chr7	71823062	22	-32.65	-26.47	No	Yes		
chr16	22646201	45	23.91	40.57	No	No		
chr8	12562048	17	-25.82	-32.17	Yes	Yes		
chr19	25794562	12	26.37	31.06	Yes	Yes		CpG Shore
chr19	11263251	20	28.21	28.64	No	Yes	TSS:3117 | TES:1592345	CpG Shore
chr20	21435758	21.5	25.40	33.18	Yes	Yes	Intron:1307063 | TSS:1304834	CpG Shore
chr13	30469587	17	44.95	25.00	No	No		
chr3	163597961	21.5	-28.65	-28.26	No	Yes		
chr1	180796208	55	-24.46	-36.59	Yes	No	Intron:1566058	
chr12	27354731	40	37.50	25.38	No	No		
chr16	51574127	14	33.33	26.15	Yes	No		

chr15	55010065	17	30.63	27.06	No	Yes		
chrX	3065855	58.5	-23.38	-49.04	No	No		
chr11	74988609	18.5	24.44	36.47	Yes	Yes		
chr4	6646359	17.5	31.58	26.67	No	Yes		
chr2	161542193	23.5	36.23	25.56	No	No		
chr9	37147910	13	-32.79	-26.28	Yes	Yes	Exon:1308527	
chr11	32440972	49.5	23.35	48.12	Yes	No		
chr19	31095893	19.5	25.00	33.88	Yes	Yes		
chr10	86738786	33	-25.95	-31.48	No	No	Intron:1307496	
chr5	98970058	24	-27.50	-29.17	Yes	Yes		
chrX	26655442	22	-34.62	-25.75	No	Yes	TSS:2293497	CpG Shore
chr7	67560213	67.5	-36.23	-25.46	No	No	Intron:1307535	
chr2	170301028	15.5	25.69	31.82	No	No		
chr6	6826623	51.5	48.47	24.90	No	No	Closest gene:1306111	
chr4	17542210	16.5	-24.02	-38.07	No	No		
chr15	9321751	36.5	-26.15	-30.93	Yes	No	Intron:3858	
chr12	15324919	14	33.33	25.97	No	Yes	TSS:628633	CpG Shore
chr3	112681793	20	23.08	65.74	No	No	Intron:620908	
chr9	38888499	18	-27.08	-29.44	Yes	Yes	Intron:1560724	
chr3	41410832	68.5	-31.43	-26.53	No	No	Intron:1302949 | 1564504	
chr15	30834809	15.5	36.97	25.19	Yes	No	Closest gene:2322476	
chr13	105016894	25	-26.07	-30.93	Yes	No		
chrX	91985239	17.5	26.25	30.62	No	Yes	Exon:1559832	CpG:_38
chr2	244906908	29	-32.87	-26.05	Yes	No		
chr17	12824156	26	31.48	26.47	Yes	Yes		
chr19	13245397	28.5	-30.43	-26.73	No	No		
chr19	19275330	14.5	-25.85	-31.14	Yes	Yes		CpG Shore
chr9	7095327	15	-24.18	-36.36	Yes	Yes		
chr18	80502646	21.5	24.03	36.97	Yes	No		

chrX	78423749	18	24.46	35.10	Yes	No		
chr10	87558546	12.5	38.95	25.00	No	No		
chrX	92511163	45.5	-26.43	-30.08	Yes	No	Exon:1564806	CpG:_21
chr5	138707199	20.5	24.69	34.34	Yes	Yes	Intron:3453	
chr20	55068435	14	-28.57	-27.78	Yes	No		
chr1	96400632	22	-23.08	-55.42	No	No	Exon:1308141	CpG:_22
chr8	65389862	18	-33.24	-25.84	Yes	Yes		
chr2	188521522	32.5	43.75	24.81	No	No		
chr2	217453893	16.5	35.42	25.30	Yes	Yes		
chr10	48933617	18	27.78	28.46	Yes	Yes		CpG Shore
chr2	139753627	26	62.35	24.46	No	No		
chr4	5960369	123.5	-29.73	-26.93	No	No		
chr1	167345536	30	50.49	24.52	No	No	TSS:1565905	
chr7	55797468	17	26.19	30.25	Yes	Yes	Intron:620728	
chr1	47582169	18.5	26.67	29.61	No	Yes		
chr4	105502595	17	28.79	27.38	Yes	No	Intron:1561512	
chr5	155699180	33.5	-40.65	-24.90	No	No	Exon:708543	
chr8	32540617	19	22.97	57.35	No	No	Intron:2583	
chr13	97393702	21.5	-23.83	-37.04	No	Yes	TSS:1582859	CpG Shore
chr5	128230893	36.5	-25.48	-31.11	Yes	No	Exon:1359481	CpG:_28
chr1	233762502	27	-22.89	-55.39	No	No		
chr7	85517215	18.5	23.31	41.58	No	No		
chr5	59137426	15.5	27.44	28.34	No	No	Exon:1306576 | TES:1309547	CpG Shore
chr11	69206212	13.5	28.57	27.27	Yes	Yes	Intron:62063	CpG Shore
chr7	127170753	22.5	-24.36	-34.17	Yes	Yes		
chr2	198940159	27.5	-30.45	-26.32	Yes	Yes		
chr4	134240480	19.5	24.62	33.33	No	No	Intron:1594800	
chr18	30674062	24	-31.82	-25.82	No	No	Exon:1587258	CpG Shore
chr1	43029063	64.5	23.17	41.87	No	No		

chr8	46589972	66.5	25.09	31.53	No	No		
chrX	82270264	18	25.93	30.04	Yes	Yes		CpG:_51
chr5	138903059	41	22.92	47.88	No	No	Closest gene:1587151	
chr6	105857710	21.5	34.23	25.11	No	No	Exon:1306115	CpG Shore
chr2	188483858	13	-28.41	-27.27	No	No		
chr20	6309649	22.5	24.62	33.23	Yes	No	Intron:1307063	
chr3	10538391	56	27.55	27.95	No	No	Intron:1564504	
chr10	14946511	34.5	24.67	32.95	No	No		
chr10	86738785	49.5	22.92	46.46	No	No	Intron:1307496	
chr10	63945210	49	-47.29	-24.21	No	No		
chr8	120095636	16.5	-29.41	-26.67	Yes	Yes		
chr20	20132987	17	-30.91	-26.01	Yes	No	Intron:1307063 | 1306871	
chr10	86360421	14.5	24.76	32.52	Yes	No		
chr12	42299156	53	22.90	46.34	No	No	Exon:1593434	
chr14	40978307	16	30.48	26.14	No	No		
chr3	161247659	18.5	27.06	28.47	Yes	Yes		
chr9	91990893	42	-23.21	-39.87	No	No		
chr7	55981656	46	31.32	25.74	No	No		
chr2	39398537	38.5	28.06	27.27	No	No		
chrX	22700361	17	27.62	27.62	Yes	Yes	Intron:1562735	CpG:_145
chr10	14946560	37.5	-22.83	-45.64	No	No		
chr3	162012228	36	28.70	26.84	No	Yes		
chr3	34086169	18.5	22.73	48.91	Yes	No	Intron:1564504	
chr17	13509961	20	34.27	25.00	Yes	Yes		
chr10	102909334	21	50.00	24.00	Yes	No		
chr10	10528079	17.5	-34.12	-25.00	Yes	No	Exon:1564638	
chr6	137167868	14.5	23.53	36.36	No	No		
chr10	16596920	21	30.55	25.91	Yes	Yes	Closest gene:620520	CpG Shore
chr15	32539354	40	25.43	30.21	Yes	Yes	TSS:69081	

chr3	88729464	29	22.74	45.84	No	No	Intron:1564504	
chr4	172052084	34.5	26.67	28.57	Yes	No		
chr14	17526889	39.5	-23.33	-37.50	No	No		
chr10	55988352	21	25.88	29.55	No	No		
chr4	155760389	15	-28.99	-26.67	Yes	No	Exon:628850	
chr9	13041702	20.5	-57.79	-23.85	Yes	No	Exon:1305956	CpG Shore
chr12	6028934	19	26.88	28.24	Yes	Yes		
chr8	115873664	57	23.08	39.08	No	No	Intron:1309232	
chr4	168527319	24.5	-22.73	-44.89	Yes	No		
chr16	9852120	22.5	23.83	34.10	Yes	Yes	Exon:727964	
chr20	4094804	49	31.43	25.44	No	No	Exon:1303267 | Intron:1307063 | TSS:1359601 | TES:1303059	CpG Shore
chr5	170257134	24.5	-41.11	-24.11	No	Yes		
chr6	71816420	12.5	27.78	27.27	Yes	Yes	Exon:1311990	
chr5	60130439	22.5	22.67	45.49	No	No	TSS:1307079 | TES:620851 | 1308910	
chr2	87430942	20	-29.17	-26.32	Yes	No		
chr9	45534315	13	-25.00	-30.77	Yes	No		CpG:_58
chr20	47631981	32.5	-22.48	-51.26	Yes	No		
chr8	123862003	18.5	-39.29	-24.22	Yes	No		
chr3	145157219	15	28.65	26.67	Yes	No	Intron:2325086	
chr2	333355	13.5	-31.90	-25.17	Yes	No		CpG:_34
chr3	11009208	46	31.87	25.14	No	No	Intron:1309399 | 1564504	
chr1	91774413	30.5	37.00	24.43	No	Yes		
chrX	91985116	17.5	25.00	30.62	No	Yes	Intron:1559832	CpG:_38
chr12	34220934	31.5	22.32	59.03	No	No	Intron:1561461	
chr1	91774407	17.5	-23.04	-38.89	Yes	No		
chr3	10395907	44	-35.64	-24.61	No	No	Intron:1564504	
chr20	6352616	21	56.67	23.74	Yes	No	Intron:1307063	
chr2	115350528	83	-24.59	-31.42	No	No		

chrX	102692948	15.5	23.71	33.97	Yes	Yes		
chr8	74213153	23.5	27.31	27.27	No	Yes		
chr11	85353340	26	38.19	24.17	Yes	No		
chr5	165034123	60.5	29.71	25.88	No	No		
chr12	7925401	47.5	-22.98	-38.82	No	No	Intron:2621	
chr5	169542902	36.5	-27.51	-27.16	No	No	Intron:1586262	CpG Shore
chr16	81985135	15	-24.24	-32.14	Yes	Yes		
chr10	106718789	59	30.42	25.50	No	No		
chr14	55204088	18	43.18	23.81	No	No		
chr5	21679515	15	-25.96	-28.82	Yes	No		
chr6	102916269	25	46.47	23.76	No	No		
chr17	50313634	15	24.21	32.21	No	Yes		
chr10	88408506	24.5	23.28	36.00	No	No		
chr6	25415293	88	-26.01	-28.64	No	No		CpG Shore
chrX	81478012	12	28.76	26.26	Yes	No		CpG:_172
chr8	115007953	44.5	23.35	35.06	No	No	TSS:708524	
chr1	131828627	38.5	-38.42	-24.03	No	No		
chr7	118164396	34.5	29.59	25.76	No	No	Intron:735027	CpG Shore
chr14	33645382	58.5	27.71	26.89	No	No		
chr3	163326839	19.5	-29.45	-25.80	Yes	Yes		
chr5	152983417	14.5	23.33	35.00	Yes	Yes	TSS:1311879 | TES:1310179	
chr3	54634366	28	-29.95	-25.55	Yes	No	Intron:621886 | 1564504	
chr20	35506669	20.5	22.86	38.39	No	No	Intron:1307063	
chr10	27600352	21.5	22.66	40.66	Yes	Yes	TSS:2650	
chr17	14634391	18	25.66	28.85	No	Yes		
chr4	101064177	17.5	23.94	32.31	Yes	Yes		
chr1	220327560	19.5	-28.62	-26.15	No	Yes	TSS:1585019	CpG Shore
chr14	100826806	19	-31.58	-25.00	Yes	No		
chr13	12987244	15	-26.47	-27.78	Yes	No	TSS:1305969	

chr10	76530884	16	-31.37	-25.00	Yes	No		
chr4	70963826	25.5	27.14	27.11	Yes	Yes	Closest gene:1332872	
chr10	106811662	22	26.54	27.65	Yes	Yes		CpG Shore
chr19	53476389	14	25.79	28.48	Yes	No	Intron:1310002	
chr20	44071875	85	22.80	37.58	No	No	Intron:1307063	
chr10	8396874	22.5	-23.88	-31.94	Yes	Yes	Exon:1311210	
chr11	84934314	14.5	25.65	28.57	Yes	No		
chr1	180639225	26	32.18	24.79	Yes	Yes		
chr1	42023595	50	-40.69	-23.62	No	No		
chr19	53902276	19.5	22.94	36.23	Yes	Yes		
chr12	21557793	18	-47.08	-23.39	No	Yes	Exon:69284	CpG:_59
chrX	134368568	29.5	27.18	26.84	Yes	No		
chr11	82032921	102	23.03	35.35	Yes	No		CpG:_18
chrX	104087133	26	39.09	23.64	No	No		
chr5	169327272	19	31.82	24.77	Yes	Yes	Intron:1303132	
chr3	10970670	24	36.11	23.92	Yes	Yes	Intron:1564504	
chr1	75333816	18.5	32.33	24.60	No	Yes		
chr19	52511860	80.5	27.12	26.76	No	No		
chr15	18496016	18	31.58	24.83	No	Yes		
chr20	38447642	22.5	-21.97	-51.32	No	No	Intron:1307063	TSS:1560095	TES:2322997	
chr1	120781293	13.5	-33.33	-24.29	Yes	Yes	Intron:1308000	
chr6	129192265	34.5	-33.33	-24.29	No	No	Intron:1304563	
chr3	151362975	25.5	-25.65	-28.26	Yes	Yes		
chr1	51504891	18.5	27.78	26.26	Yes	Yes		
chr17	94875217	22	-25.64	-28.28	No	Yes		
chr15	39860489	105.5	-24.44	-30.21	No	No	Exon:1560876	
chr2	30880982	40.5	21.86	64.89	No	No	Closest gene:2272	
chr3	126403317	21.5	-30.32	-25.00	No	No	Exon:1311623	
chr1	12988651	15.5	26.67	27.08	Yes	Yes		

chr1	30599438	27.5	-31.90	-24.60	Yes	No	Intron:1311599	CpG:_33
chr14	80672503	27	-22.56	-37.85	Yes	No		
chr9	8510753	39.5	-34.44	-24.02	No	No	Intron:1311386	CpG:_188
chr20	13029148	73	-34.08	-24.07	No	No	Exon:1565460	CpG Shore
chr9	8348522	21.5	-27.30	-26.47	No	Yes		CpG Shore
chr2	48230029	30.5	28.57	25.60	No	No		
chr1	187501149	75	-23.07	-34.48	No	No	Intron:1306553 | TSS:620173	
chr3	154204577	16.5	-26.64	-27.06	Yes	Yes	Intron:735184	
chr18	71888949	15	-30.00	-25.00	No	No		CpG:_76
chrX	46330664	49	23.67	31.65	Yes	Yes		CpG:_101
chr5	17903259	20	29.17	25.22	Yes	No		
chrX	7336710	26	31.90	24.46	Yes	Yes	Closest gene:1566035	
chr14	22476609	16	-22.69	-36.36	No	Yes	Intron:620895	
chrX	22019951	43	48.41	23.18	No	Yes		
chrX	159848760	28	28.20	25.75	Yes	Yes		CpG:_61
chr6	98430377	22	-22.73	-35.71	Yes	No		
chr6	132355807	38	39.11	23.46	No	No	Intron:1311128	
chr5	171858538	14.5	41.18	23.33	No	Yes		
chr20	29975236	33	25.88	27.60	Yes	Yes	Intron:1307063 | 1310622	CpG Shore
chrX	131483384	39	31.14	24.68	No	No		
chr9	103064299	18	23.68	31.37	No	No		
chr14	62679403	15.5	26.01	27.38	No	Yes		CpG:_38
chrX	36884763	27.5	-26.07	-27.31	No	Yes		CpG:_33
chr8	62623860	25.5	32.67	24.18	No	Yes	Closest gene:71065	
chr1	173941192	46.5	32.78	24.15	No	No	Intron:1309000	
chr17	80688543	17.5	-36.63	-23.59	No	Yes		
chr4	180393967	20	24.15	30.09	Yes	Yes	Closest gene:1359322	
chr5	171274258	28.5	40.15	23.33	Yes	No		
chr4	163096516	53.5	24.70	29.32	No	No	Intron:62083	

chr8	103488825	12.5	28.95	25.17	No	No	Intron:1309140	
chrX	127309384	47.5	34.05	23.87	Yes	Yes		CpG:_74
chr19	37335203	44	22.90	34.50	No	No	Intron:1310091	
chr10	19357687	31	22.55	36.40	Yes	Yes	Intron:1564189	
chr5	165477718	17.5	-22.27	-38.46	Yes	Yes		
chr8	126623891	16	29.52	25.00	No	Yes		
chr8	22362847	25.5	-24.84	-29.04	Yes	No	Intron:1308472	
chr12	15198029	25.5	21.90	44.24	Yes	Yes	Intron:1308787	CpG Shore
chr2	43099619	22.5	27.90	25.71	Yes	No		
chr20	18009135	49.5	30.69	24.65	Yes	Yes	Intron:1307063	
chr13	47279736	18	-26.39	-26.92	Yes	No	Intron:620055	
chr8	125818151	56.5	-23.21	-32.64	No	No		
chr2	196924462	21	-25.27	-27.98	Yes	Yes	Intron:1308694	
chr2	79684197	22.5	27.30	26.10	Yes	Yes	Intron:1308360	
chr1	191785748	35	28.08	25.54	Yes	Yes		CpG Shore
chr10	107842863	25.5	-22.04	-40.85	Yes	Yes		
chr1	207705045	42.5	37.38	23.40	No	No	Exon:1310680	CpG Shore
chr3	154407224	37	37.37	23.40	No	Yes	TSS:1305296	
chr1	21587252	23.5	24.96	28.62	No	Yes		
chr12	28413702	36.5	-34.80	-23.65	No	No		
chr18	74267077	18	25.75	27.31	Yes	Yes		
chrX	61434485	19.5	-22.73	-34.62	Yes	No		
chr13	71920515	45	-29.84	-24.91	No	Yes	Closest gene:1590898	
chr5	153425072	25	32.54	24.00	No	Yes		
chrX	33085142	22	-23.00	-33.33	Yes	Yes		
chr19	21207515	16	24.55	29.09	Yes	Yes		
chr4	177211093	18	28.21	25.33	Yes	Yes		
chr17	63060088	16.5	22.62	34.98	Yes	Yes	Intron:2324280	
chr6	47899786	111.5	-24.44	-29.17	No	No	Exon:1560694	CpG Shore

chr5	144633218	52.5	22.44	35.77	No	No		
chrX	70002304	42.5	-31.82	-24.11	No	No		
chr18	50315285	32	-28.97	-25.00	No	No	Exon:1306824	
chr2	224890589	15	31.75	24.11	No	No	Closest gene:1309061	
chr4	165225148	37.5	-22.36	-36.20	Yes	No		
chr20	21860309	19	22.71	34.44	No	Yes	Intron:1307063	
chr18	31519334	23	-22.37	-36.18	Yes	No		
chr7	72867724	24.5	-25.15	-27.78	Yes	Yes	Intron:1561191	
chr1	42023604	51	-31.28	-24.19	No	No		
chr2	176207702	24	-23.33	-31.37	No	No		
chr1	228785801	18	30.00	24.60	Yes	Yes	Intron:1307714	
chr12	12221218	21	-21.58	-47.62	No	No	Intron:1307540	
chr7	88891439	20	23.67	30.36	Yes	Yes		
chr17	26729547	19.5	26.49	26.57	Yes	Yes	TSS:1309677	
chrX	21417768	31.5	28.34	25.07	Yes	No		
chr1	176103645	24	-32.00	-23.92	No	Yes		
chr16	38299529	14.5	21.68	43.75	Yes	No		
chr4	157846940	17.5	-29.85	-24.60	Yes	No	Intron:1593191	CpG Shore
chr13	91979772	32	53.85	22.75	No	No	Intron:1562609	
chr20	16635973	45	24.93	28.23	Yes	No	Intron:1307063	
chr9	89095414	13.5	-28.57	-25.00	Yes	No	Intron:1310850	
chr5	80486917	33	26.14	26.67	No	No		
chr14	33645381	28.5	-22.90	-32.81	No	No		
chr3	10242632	40.5	26.96	25.96	No	No	Intron:1305882 | 1564504	
chr4	180741360	13.5	-23.33	-31.06	Yes	Yes	Intron:1359322 | 3679	
chr3	161154773	90.5	22.49	34.60	No	No		
chr10	90878541	13.5	-21.54	-45.67	Yes	No		
chr9	9800664	17.5	35.48	23.33	Yes	Yes	Exon:1562008 | TES:1559787	
chr4	166134549	15.5	-35.29	-23.33	No	No		

chr4	186973700	65.5	23.36	30.76	No	No	Intron:1309444	
chr1	85496323	18.5	25.00	27.73	No	No	Exon:1309965	
chr5	1711990	21.5	23.33	30.82	No	No	Closest gene:1302993	
chr5	144546920	21	-21.80	-39.81	Yes	Yes		
chr8	48124932	18.5	-24.62	-28.41	Yes	No	Exon:1305033	
chr6	130725994	28	30.84	24.07	Yes	Yes		
chr20	11459196	55	23.46	30.44	No	No	Intron:1307063 | 1305581	
chrX	41270166	21	27.37	25.48	No	Yes		CpG:_84
chr1	264672290	15	-21.52	-45.00	Yes	No		
chr3	6628923	18.5	25.29	27.27	No	Yes	Intron:3610 | 1564504	
chr4	6126297	17.5	22.96	32.14	Yes	Yes		
chr6	123873719	15	22.92	32.28	Yes	No		
chr13	46339296	28	-24.76	-28.07	Yes	Yes	Intron:1309674	CpG:_23
chr1	181591812	44.5	-29.76	-24.44	No	No	Intron:628803	
chr2	253513948	86	-29.10	-24.76	No	No		
chr8	11341295	91	-32.47	-23.68	No	Yes	Intron:1305268	
chr3	156567815	86.5	-26.65	-26.11	No	No	Exon:1306552	
chr4	50352803	56.5	28.79	24.89	Yes	No	Closest gene:620735	
chrX	82059843	16.5	-26.47	-26.20	Yes	Yes		CpG:_16
chr1	195168696	76	-36.85	-23.08	No	Yes		
chr1	209316664	16	-47.62	-22.73	No	Yes	Exon:620211 | TES:621628	CpG:_53
chr19	38405752	13	-22.63	-33.33	Yes	Yes		
chrX	116442808	14.5	24.11	28.95	Yes	Yes		
chr15	40845119	19.5	-50.74	-22.67	Yes	No		CpG Shore
chr8	62666722	27.5	25.49	26.98	Yes	Yes	Exon:71065	
chr12	46032432	36	26.19	26.38	No	No	Intron:1311871	
chr1	230281454	20	23.81	29.47	Yes	Yes		
chr3	151327933	18.5	-21.79	-38.67	No	No		
chr10	94752248	14.5	-28.89	-24.73	Yes	Yes	TES:1559436	

chr12	27038726	20	30.00	24.20	No	Yes		
chr5	61619293	13.5	-25.45	-26.92	Yes	No	Intron:1305465	
chr12	16832053	19	30.77	23.94	Yes	No	TSS:628728	CpG:_56
chr1	180111354	15	21.52	42.56	Yes	Yes		
chr1	247344482	52	-31.88	-23.68	No	No	Exon:2721	
chr3	169500618	14	21.43	44.29	Yes	Yes		
chr13	99319683	88	21.69	39.93	No	No		
chr12	44276601	23.5	-25.33	-26.98	Yes	Yes		
chr12	43577785	17.5	-24.44	-28.26	Yes	Yes	Intron:620277	
chr19	50479401	18.5	-26.98	-25.57	Yes	Yes	Intron:1304884	
chr6	15177349	22.5	24.34	28.42	Yes	No	Intron:628659	
chr7	61415322	16.5	30.56	23.94	No	No		
chr10	95391691	51.5	-35.71	-23.08	No	No	Intron:1561299	
chr6	78018389	22.5	-30.68	-23.91	No	Yes	Closest gene:1560556	
chr13	102602898	16.5	45.22	22.65	No	Yes		
chr17	16136596	48.5	29.17	24.45	No	No		
chr4	126577032	49	-21.90	-37.05	No	No	Exon:1306723	
chr5	153821026	24.5	25.64	26.67	Yes	Yes		
chr5	154748979	22	23.03	31.25	Yes	No	TES:620210	
chr18	42535485	15.5	22.92	31.43	Yes	No		
chr3	142333881	25	-21.28	-49.24	No	No	Closest gene:1308904	
chrX	42975484	21	24.73	27.63	No	Yes		
chr1	225567838	35.5	24.22	28.36	No	Yes	Intron:1304888	
chr5	154330062	54	-21.53	-40.24	Yes	No	Intron:1308320	
chr9	105483541	48.5	27.06	25.31	No	No	Intron:1308319	
chr15	102997318	22	25.08	26.98	Yes	Yes		
chr7	74147562	17.5	-22.90	-31.25	Yes	No		CpG Shore
chr8	65428628	16	21.44	40.83	Yes	Yes		
chr7	62712095	26.5	24.73	27.47	Yes	No		

chr12	25965602	16	21.74	37.30	Yes	No		CpG Shore
chr5	4917095	41.5	21.09	63.85	Yes	No		
chrX	122789525	18.5	21.96	35.59	Yes	No		
chr8	111170411	17.5	24.68	27.45	No	Yes		
chr17	6289267	20	-23.26	-30.00	Yes	Yes	Intron:2448	CpG Shore
chr14	4063039	17	22.83	31.25	Yes	Yes		
chr1	192826740	48	21.21	47.08	No	No		
chr1	16762946	47	21.41	42.42	Yes	No		
chr7	74634664	16.5	-29.04	-24.24	Yes	Yes	Intron:628801	
chrX	93834223	30	28.95	24.25	Yes	No		
chr5	154751943	43.5	29.37	24.10	Yes	Yes	Intron:620210	
chr2	27699487	21.5	21.33	43.65	No	Yes		
chr1	80317676	23	-28.57	-24.44	No	Yes	Intron:2735	CpG Shore
chr1	259191587	64.5	-57.93	-22.28	No	No		
chr7	139588207	24.5	-24.67	-27.37	No	Yes		
chr1	222517403	24	-32.89	-23.26	Yes	Yes		
chrX	2236191	22	21.79	36.19	Yes	Yes	Intron:1561678	
chr1	88244525	23	-27.27	-25.00	Yes	No	TES:1308334	
chr8	57276158	28.5	23.51	29.31	No	Yes	TES:1311242	
chr7	56572748	48.5	-22.01	-34.63	Yes	No	TES:1306474	
chr3	78633450	23	26.09	25.88	Yes	Yes	Intron:1564504	
chrX	92511162	34	-47.24	-22.33	No	No	Exon:1564806	CpG:_21
chr10	14732592	19	-22.67	-31.58	Yes	Yes	Closest gene:1308075	
chr2	141024040	13	33.76	23.08	Yes	Yes		
chr16	9774052	16.5	22.43	32.38	Yes	No		
chr15	2680595	49	-21.30	-42.73	No	No	Intron:1310229	
chrX	15397953	43	-23.70	-28.69	Yes	No		
chr9	90198597	52.5	21.06	49.25	No	No	TSS:1562478	
chr5	108908023	14.5	24.76	27.18	Yes	Yes	TES:2323	

chr5	62436419	83.5	-24.86	-27.08	Yes	No		
chr5	5607777	13	31.82	23.33	No	No	Intron:708554	
chr1	240964519	23.5	-32.00	-23.31	No	Yes		
chr16	75838459	16	37.50	22.67	Yes	No	TES:1560642	
chr17	34502117	21	21.52	37.78	Yes	Yes		
chr16	65948712	42.5	-21.44	-38.44	No	No		
chr5	165887920	35	21.94	34.54	No	No		
chr13	97502712	32	-21.88	-34.76	No	No	TES:1582859	
chr7	139174926	44.5	-26.03	-25.71	Yes	No	Intron:1596738	CpG Shore
chr15	90030278	37	26.25	25.53	No	No		
chr8	124293145	21	24.88	26.95	Yes	Yes	Closest gene:1310466	
chr2	168844209	18.5	24.70	27.11	No	No		
chr6	13478694	18	28.79	24.09	Yes	Yes		
chr13	104279092	19	21.45	37.88	Yes	Yes	Intron:1303236	
chr19	14318832	31.5	-32.12	-23.17	No	No		
chr15	54251562	80.5	26.08	25.61	No	Yes	Intron:1309397	
chr9	55989534	15	22.31	32.09	Yes	Yes		
chr9	32978044	20	-24.87	-26.85	Yes	No		CpG Shore
chr3	4312040	25.5	-23.28	-29.28	Yes	Yes	Intron:1564504	
chr6	129876117	41	21.12	43.35	No	No	Intron:1309600	
chr9	33031414	28.5	23.81	28.03	No	No		
chr17	37632467	25	21.43	37.88	No	No		
chr7	136012105	55.5	20.98	48.32	No	No		
chr7	140098174	28	28.77	24.00	Yes	Yes	Intron:1359177	
chr10	110007026	32	-31.39	-23.28	No	Yes		CpG Shore
chrX	82270259	18	29.63	23.68	Yes	Yes		CpG:_51
chr2	80234763	12	21.18	41.67	No	Yes	Intron:1560828	
chrX	55880004	26	38.11	22.46	Yes	Yes		
chr11	23767751	47	25.51	25.95	Yes	No		

chr12	44659389	49	-34.24	-22.82	No	No		
chr5	135273515	18.5	20.85	54.20	Yes	Yes	TSS:1304642	
chr7	123244985	13.5	-53.33	-22.12	Yes	Yes		
chr20	8032971	25	-21.88	-33.78	Yes	No	Intron:1307063 | TSS:1588733	
chr17	95324170	17	-21.13	-41.67	Yes	No	Exon:1305841	CpG Shore
chr4	5468600	87.5	-25.81	-25.64	No	No		
chr9	56976314	14.5	-28.49	-24.04	Yes	Yes	Exon:1359668	
chr4	126165328	15.5	28.37	24.11	Yes	Yes		
chr19	10174035	52.5	-25.86	-25.57	Yes	Yes	Intron:735198	
chr10	21369454	34.5	-21.10	-41.80	No	No	Intron:727907	
chr1	120466103	43	-27.44	-24.59	No	No	Exon:3246	
chr4	11044489	22	25.45	25.89	No	Yes		
chr5	154729958	14	-22.73	-30.25	Yes	No	TSS:620554	CpG Shore
chr7	121950516	17	-30.00	-23.51	No	No		
chr10	85831956	37.5	26.38	25.00	No	No	Intron:1308264	CpG Shore
chr4	168087090	17.5	23.33	28.71	No	No		
chr18	74402840	94.5	-25.51	-25.79	No	No		
chr7	77319129	17.5	22.22	31.67	Yes	Yes		
chr10	62471184	17.5	23.81	27.78	Yes	Yes		CpG Shore
chr15	34206371	18.5	21.30	38.46	Yes	Yes	Closest gene:735036	
chr18	77019605	36	25.63	25.67	Yes	Yes		
chrX	105476778	18.5	23.80	27.78	Yes	Yes		
chr10	13871426	18.5	28.79	23.81	Yes	Yes	Exon:3908	
chrX	89376334	16.5	-20.80	-55.20	Yes	No	Intron:621119 | TES:1585896	
chr3	160871569	17.5	29.46	23.56	Yes	No		
chr4	117362715	15.5	21.67	34.62	No	No	Exon:727874 | 1561494	
chr3	105197202	17.5	-22.73	-30.04	Yes	Yes	Exon:1595953	CpG:_193
chr18	25271443	14.5	-22.22	-31.67	Yes	Yes	Intron:1586479	
chr1	185853390	19	-21.67	-34.51	No	No	Exon:620009 | TSS:1311568 | TES:1565363	

chr20	53048240	19	23.48	28.33	Yes	Yes		
chr14	46073010	12.5	-36.36	-22.44	No	Yes	TSS:1560201	CpG Shore
chr2	148566318	15	22.73	30.00	Yes	Yes	TES:1307852	
chr10	90102065	90	-29.93	-23.38	No	No	Intron:1549767	
chr9	9106699	18	-27.45	-24.36	No	No	Intron:1307167	
chr1	53394721	48	23.75	27.62	No	No		
chr9	74377680	33	-25.76	-25.37	Yes	Yes	Intron:1359338	
chr1	158324271	17.5	-22.78	-29.80	Yes	No	Exon:1309890	
chr5	99697609	18.5	-26.32	-25.00	No	No		
chr4	165414095	19	-24.50	-26.67	Yes	No	Closest gene:1589959	
chr1	233914332	16.5	-34.22	-22.60	Yes	No	Intron:1308660	
chr8	77233443	75.5	21.38	36.37	No	No		
chr1	96400631	22.5	20.83	45.55	No	No	Exon:1308141	CpG:_22
chr18	80690977	19	-26.99	-24.62	Yes	Yes		
chr4	157568720	23	-24.24	-26.91	No	Yes		
chr9	9911652	40.5	-22.50	-30.32	Yes	No	Intron:1559518	CpG:_27
chr2	203133963	16.5	-21.43	-35.83	No	Yes		CpG Shore
chr7	136506749	21	-20.73	-49.52	Yes	No	Intron:619982	
chr12	43521246	29.5	39.85	22.16	Yes	Yes	Exon:1307200	
chr9	70695978	16	21.50	34.62	Yes	No	Intron:70879	
chr3	153478994	23	-25.55	-25.42	Yes	Yes		
chr13	45455460	65.5	20.75	47.87	No	No	Exon:620911	
chr3	76355245	19	34.92	22.46	Yes	No	Intron:1564504	CpG Shore
chr16	79431970	31.5	-26.52	-25.00	Yes	Yes	Exon:1563576	CpG:_207
chrX	113432996	67	24.57	26.54	No	No		
chr6	91127105	21	-56.67	-21.79	No	No	Intron:1589026	
chr9	88777977	54	-31.21	-23.03	No	No	Intron:1309244	
chr20	10284889	80	20.88	42.12	No	No	Exon:69407	
chrX	92511158	34	-28.86	-23.54	No	No	Exon:1564806	CpG:_21

chr6	98899029	16.5	-45.71	-21.88	Yes	No	Exon:708531	
chr2	196761482	18.5	22.22	30.95	Yes	Yes		
chr3	168776084	37.5	25.59	25.27	Yes	Yes		
chr9	87785542	21	-32.29	-22.74	Yes	No		
chr17	14373959	21.5	-24.85	-26.19	Yes	Yes		CpG:_18
chr3	9030617	55	-25.64	-25.22	No	No	Intron:1359465 | 1564504	
chr7	138169458	61	28.32	23.78	No	No	Intron:1561571	
chr5	163718385	48	27.44	24.13	No	No	Exon:3879	
chr2	176448535	16.5	28.34	23.75	Yes	Yes	Exon:1563262	
chr14	61456388	21	-20.74	-45.78	Yes	No		
chr14	81341673	13.5	26.84	24.55	Yes	Yes		CpG Shore
chr5	17190668	20	25.48	25.29	Yes	No		
chr20	14059255	27	-21.38	-35.42	No	No	Intron:1307063 | 1307993	
chr9	36515611	58	21.84	32.22	No	No		
chr1	192071175	31	-25.13	-25.59	Yes	Yes	TSS:1306744	CpG Shore
chr5	155608470	14.5	-24.51	-26.37	Yes	Yes	TSS:1564232	
chr3	160047397	26.5	26.00	25.00	Yes	Yes	TSS:1307948	
chr10	49428119	28.5	-31.10	-22.96	No	Yes	Exon:2321712	
chr1	258555526	22	33.95	22.46	Yes	No		
chr9	73700171	22.5	39.59	22.02	Yes	Yes	TSS:1596071 | TES:2300155	
chr3	162725460	16.5	27.62	23.95	Yes	No		
chr13	45707313	13	-28.05	-23.78	No	Yes		
chr6	102879094	13.5	27.83	23.81	Yes	Yes		
chr11	13054019	24	-20.65	-46.71	Yes	No	Intron:620167	
chr14	3830706	13.5	-26.62	-24.62	No	Yes		
chr18	74377794	17.5	25.56	25.08	Yes	No		
chr20	45218800	42.5	24.98	25.88	Yes	Yes		
chr1	17342482	16	-20.67	-45.00	No	Yes		
chr5	100371808	31.5	-20.51	-54.35	No	No		

chrX	91985276	18	21.43	34.45	Yes	Yes	Exon:1559832	CpG Shore
chr20	4185769	14.5	22.32	30.00	No	Yes	Intron:1307063	
chrX	28725655	14.5	-21.90	-31.47	No	No	Exon:1594217	CpG Shore
chr7	110541199	14	21.43	34.23	No	No	Intron:1309461	CpG:_35
chr8	48223320	20.5	20.83	40.24	Yes	No	Intron:620138	CpG Shore
chr2	26923263	40.5	24.56	26.13	No	No	Intron:2325067	
chrX	137265330	16.5	32.35	22.60	Yes	Yes		
chr4	172286151	55.5	-20.67	-44.16	No	No		
chr8	95242406	16.5	-28.57	-23.50	Yes	No		
chr9	29957097	16.5	21.11	36.36	Yes	No		
chr1	260125962	17	-24.64	-25.96	Yes	No	Intron:1307427	
chr8	103606033	14	-22.27	-30.00	Yes	Yes	Exon:3543	CpG:_29
chr1	228824049	36.5	25.12	25.29	No	No	Intron:1307714	
chrX	137265300	16.5	32.03	22.60	Yes	Yes		
chr19	13049855	25	-38.10	-21.94	No	No		
chr6	25580623	31.5	-22.12	-30.56	Yes	Yes		
chr19	25058752	18.5	-23.77	-26.92	Yes	Yes		
chr14	57322193	37	23.64	27.05	Yes	Yes		CpG Shore
chr8	11494691	21.5	23.00	28.32	No	No	Exon:1311015	
chr1	90704398	23	-24.18	-26.36	Yes	Yes	Closest gene:1310544	CpG:_30
chr17	28976289	22	21.28	34.66	Yes	No		
chr1	91206380	16.5	34.56	22.22	No	Yes		
chr19	25835920	42	-24.59	-25.91	Yes	No		CpG:_54
chr3	10242631	36	-20.59	-44.03	No	No	Intron:1305882 | 1564504	
chr3	160104138	37	-20.77	-40.05	No	No		
chr3	52692425	16	23.33	27.37	No	Yes	Intron:1564504 | TSS:1308499	CpG Shore
chr10	106338628	32.5	61.08	21.53	No	No		CpG Shore
chr15	105971652	23.5	-22.83	-28.50	Yes	No		
chr8	58794560	19.5	26.36	24.44	No	No	Intron:1589860	

chr8	45253508	21.5	28.32	23.41	Yes	Yes		
chr3	164364964	70.5	24.93	25.52	No	No		
chrX	88087582	20.5	50.00	21.54	No	No		
chr5	166683984	53.5	-20.56	-43.72	No	No	Intron:1306458	
chrX	10253851	12	21.69	31.67	Yes	No		CpG:_48
chr5	154695580	72.5	-20.65	-41.35	No	No	Intron:619713	
chr20	11110592	15.5	28.48	23.33	No	Yes	Intron:1307063 | TES:628861	
chr7	123288822	16	21.05	35.71	Yes	No		
chr3	5970173	54.5	-28.21	-23.39	No	No	Intron:1564504 | TSS:1306074	
chr12	13466476	22	20.59	42.66	Yes	Yes		
chr6	40491059	31	21.01	36.09	No	No		
chr6	49059561	21.5	-21.67	-31.67	Yes	Yes		
chr8	123580344	16.5	-21.11	-35.00	No	Yes		
chrX	61600136	24	34.97	22.09	Yes	Yes		CpG:_32
chr6	32749543	72.5	-23.66	-26.68	No	No		
chr1	84945594	14	30.00	22.86	Yes	Yes	Closest gene:1563223	
chr4	156425475	63	-28.56	-23.27	No	Yes	TSS:621141 | TES:1585801	
chr3	6063137	65	20.61	41.06	No	No	Intron:2489 | 1564504	
chr2	246195607	11	23.33	27.27	Yes	Yes		
chr18	4016975	23	24.68	25.56	Yes	Yes		
chr14	44838360	23.5	-36.61	-21.88	No	No	Exon:1359713	CpG:_56
chr1	198694692	13	25.00	25.13	Yes	No	Intron:620800	
chr1	116627459	49	22.41	29.09	No	No	Exon:1583615	CpG Shore
chr17	15860226	32	-25.76	-24.73	Yes	Yes		
chr4	174459316	13	-25.19	-25.00	Yes	Yes	Intron:2321499 | 62068	
chr6	91970295	18	27.37	23.64	Yes	Yes	Intron:1562028	
chr12	12904072	24.5	25.99	24.55	No	No		
chr14	10735798	24.5	20.33	47.74	No	No		CpG Shore
chr7	59908513	50.5	21.89	30.45	Yes	No		

chr14	105173825	20.5	44.65	21.52	No	Yes		
chr6	57674113	36.5	-24.62	-25.54	No	No	TSS:1312007	
chr8	126369199	28.5	22.70	28.44	Yes	No	TSS:1307844	
chrX	22729432	21.5	-20.83	-36.67	Yes	Yes	Intron:1562735	CpG:_132
chr5	140740149	29.5	30.93	22.55	No	Yes	TSS:2533	
chr17	92708812	57	21.64	31.30	Yes	No		
chr3	156652818	21	24.31	25.76	No	No		
chr11	85165212	17	-26.92	-23.81	Yes	Yes		CpG:_89
chr1	94612228	46.5	-36.36	-21.82	Yes	No		
chr6	128686677	17	29.50	22.86	No	Yes		
chr9	56100912	52.5	20.33	45.63	No	No		
chr5	165498902	15.5	-25.00	-25.14	Yes	No		
chrX	75812263	16.5	-22.22	-29.41	Yes	No		
chr2	204800991	34.5	20.53	40.78	No	No		
chr16	59781340	42	-41.40	-21.53	Yes	No		CpG Shore
chr6	133290943	20	-22.17	-29.55	No	Yes	Closest gene:708347	
chr1	207356081	30.5	36.00	21.82	No	Yes	Intron:3751	
chr11	72655905	18.5	-26.19	-24.21	Yes	Yes		
chr13	30744215	32.5	-21.51	-31.55	No	Yes		
chr12	25315151	19	23.53	26.67	No	Yes		
chr13	9106052	26.5	-21.05	-34.49	Yes	No		
chr5	7438334	33	20.64	38.46	Yes	No	Intron:1307865	
chr1	247291892	18.5	23.75	26.32	Yes	No		
chr3	68097971	21.5	-25.22	-24.92	Yes	Yes	Intron:1564504 | TES:727797	CpG Shore
chr14	92697060	19	-26.89	-23.76	Yes	Yes	TSS:1307112 | TES:2494	
chr1	118808759	13.5	25.00	25.00	Yes	No	Closest gene:1307633	
chr1	136164146	19	-25.00	-25.00	Yes	Yes	TES:1311839 | 2318548 | 1310238	
chr1	162719680	10.5	20.45	40.91	Yes	Yes	Closest gene:1334060	
chr16	56334480	56	20.75	36.96	No	No		

chr4	83375558	15	22.16	29.41	Yes	No		
chr20	45741274	28	23.78	26.25	No	Yes		
chr18	30630676	38	-24.35	-25.53	Yes	No	Intron:1590761 | 1587259 | 1598664 | 1311949 | 1305108 | TSS:1310676	CpG Shore
chr7	10417214	15	23.08	27.27	Yes	Yes	TES:1596084 | 2108 | 1308627	CpG Shore
chr6	26802122	21	27.07	23.60	Yes	Yes		
chr8	120579111	22.5	26.47	23.92	Yes	Yes	Intron:1565145	
chr12	37982646	84	32.91	22.11	No	No	Intron:1305702	
chr8	107519965	55.5	-33.68	-21.97	No	No	Closest gene:1305407	
chr7	114300900	22	22.73	27.78	Yes	Yes	Intron:1565316	
chr2	121709497	16.5	-31.75	-22.22	Yes	Yes		
chr10	87435393	54	24.04	25.66	Yes	Yes	TSS:1592062	
chr3	154424139	63	-25.62	-24.39	No	No	TES:1305296	
chr8	106210496	23	28.48	23.01	Yes	No		
chr6	7187837	46	-47.92	-21.34	No	No	TES:1306803	
chr15	35645606	17.5	22.73	27.60	No	Yes		
chr1	249744787	13.5	31.43	22.22	No	Yes		
chr11	10273261	19	-20.72	-35.96	No	No		
chr9	76486457	39.5	-30.35	-22.38	Yes	Yes		
chr15	32702764	41	21.70	30.10	No	No	Exon:1308773	
chr3	169246723	34.5	28.79	22.82	No	Yes		
chr3	38868892	47.5	23.16	26.73	No	No	Intron:1564504	CpG Shore
chr20	3018634	61.5	-21.35	-31.58	Yes	No	Exon:1302963	
chr6	1957297	44	20.59	36.82	Yes	No		
chr10	14820555	22	25.57	24.28	Yes	No	Exon:1309072	
chr6	129876028	48.5	-21.54	-30.51	No	No	Intron:1309600	
chr4	118552148	12.5	-20.45	-38.31	Yes	Yes		
chr15	82880819	33	-32.14	-22.05	Yes	Yes	Exon:1304646 | TSS:1305077	CpG Shore
chr5	147652533	20.5	-30.98	-22.22	No	Yes		

chr13	23544398	19.5	29.41	22.59	No	No	Intron:1566343	
chr3	156560445	13	-23.25	-26.62	Yes	Yes	TSS:1306552	CpG:_25
chr8	110662886	19	43.32	21.33	No	Yes		
chr6	138327884	13	-21.15	-32.31	Yes	No		
chr18	73462788	67.5	22.63	27.54	No	No		
chr2	67939480	32	23.26	26.59	Yes	Yes		
chr2	160897213	15	23.14	26.67	No	Yes		
chr13	75968680	15	-27.78	-23.08	Yes	Yes	Intron:1304620	
chr7	127634706	41.5	20.48	37.49	Yes	No		
chr19	50282058	20.5	25.25	24.40	No	Yes		CpG Shore
chr5	87128493	18	28.53	22.81	Yes	Yes		
chr1	252653309	16	21.79	29.49	Yes	No		
chr3	3300027	53	-20.49	-37.25	No	No	Intron:1564504	
chr2	2280317	11.5	-28.33	-22.86	Yes	No		
chr5	77429262	34.5	20.85	33.53	No	Yes	Intron:1306966	
chr8	9983448	21	-23.38	-26.19	No	No		
chr19	674955	32.5	-20.69	-35.10	Yes	No	Intron:1309279	
chr7	140324533	22.5	-21.34	-31.15	No	No	Intron:1359576	
chr20	36089017	40.5	-33.12	-21.78	No	Yes	Intron:1307063	
chr1	67551503	121.5	25.86	23.89	Yes	No	TES:1565545	CpG:_159
chr5	167616410	44.5	20.03	43.36	No	No	Intron:629475	
chr5	148432926	15	-34.90	-21.58	No	Yes		
chr3	135348757	29.5	20.88	33.33	No	No		
chr1	240548672	20.5	44.74	21.16	No	Yes		
chr9	105607560	29	23.35	26.13	Yes	No	Intron:2323709	
chr6	137003779	98.5	-21.47	-30.11	No	No	Exon:1307957	
chr18	50732696	19.5	21.58	29.99	Yes	No		
chr8	47482262	13.5	42.74	21.21	No	Yes	Exon:1309096 | TES:62010	
chr11	32142681	11.5	-27.27	-23.08	No	No		

chr2	182422698	13	23.02	26.67	Yes	Yes	Exon:1310250	
chr3	116793130	13	25.19	24.24	No	Yes		
chr2	121905050	20.5	-22.73	-27.10	Yes	Yes		
chr12	21731550	14	25.00	24.51	No	Yes	Exon:1598237	
chr19	24803357	25.5	40.13	21.29	No	No		CpG Shore
chr10	7046369	72	23.70	25.59	No	No	Intron:620948	
chr3	114503981	16.5	-30.95	-22.06	No	Yes		
chr17	87528110	13.5	-20.65	-34.62	Yes	Yes	Intron:1591781	
chr14	88724103	16.5	-25.15	-24.21	No	Yes		
chr10	84231763	18	30.00	22.22	Yes	No		
chr17	27883630	16	30.00	22.22	No	Yes	Intron:1303187	
chrX	175982	35.5	-33.75	-21.65	No	Yes	Exon:1563873	CpG:_68
chr13	94582130	23.5	-26.92	-23.22	No	Yes	Intron:1560022	
chr1	180229703	19.5	40.99	21.18	No	Yes	Intron:727787	
chr14	65226983	19.5	25.00	24.40	No	Yes		
chr11	32305298	16	-27.68	-22.92	Yes	No	TES:2299853	
chr7	123654587	17.5	22.22	27.88	Yes	No		CpG:_41
chr15	32702765	58	-21.84	-28.82	Yes	No	Exon:1308773	
chr4	8961929	26.5	21.37	30.46	No	No		
chr19	32079768	24	23.77	25.38	Yes	No	Intron:2535	
chr18	31492602	12.5	20.51	35.45	Yes	Yes		
chr5	60847772	28	-20.55	-35.09	Yes	No	Exon:1308974	
chr2	136832096	30	-20.95	-32.23	Yes	No		
chr3	156190264	57	20.22	38.22	Yes	No	Intron:2321	
chr1	259755601	14.5	23.64	25.48	No	Yes		CpG Shore
chr11	84899935	12.5	22.92	26.67	Yes	Yes		
chr17	16292848	20.5	-32.50	-21.71	Yes	No		
chr4	183076502	34	20.43	35.87	No	No	Intron:1560077	
chr7	117477525	18	-27.95	-22.73	Yes	Yes	Intron:1307611	

chr1	67551775	64	-22.32	-27.42	No	No	TES:1565545	CpG:_159
chr2	200784082	16.5	-37.30	-21.33	Yes	Yes	Intron:68394	
chr3	161265076	20.5	-28.00	-22.73	No	Yes		
chr16	54771362	64	-33.12	-21.65	Yes	Yes		
chr7	9925534	24.5	-20.29	-36.95	Yes	No	Exon:1309247	TES:620846	CpG:_16
chr17	78907809	22	-25.20	-24.02	No	Yes		
chr12	4954567	32	30.15	22.10	No	No	Intron:1307034	
chr1	87118688	46	27.17	23.07	No	No	Exon:1583737	CpG Shore
chr12	15142050	38	-20.86	-32.31	Yes	Yes	Intron:1308787	
chr18	31050429	17	26.46	23.33	Yes	No		
chr7	55649992	42.5	-21.11	-31.08	No	No	Exon:1305922	
chr8	18377644	22.5	26.46	23.33	No	Yes		
chr16	71157641	25.5	-27.78	-22.73	Yes	No		
chr10	74141441	21.5	25.00	24.21	Yes	Yes		
chr8	2272481	25.5	23.45	25.63	Yes	Yes		
chr4	143921032	16.5	21.05	31.25	Yes	Yes		
chr9	63300741	65.5	-27.72	-22.74	No	No		
chr7	129417273	25.5	-29.72	-22.17	Yes	Yes		
chr1	248174691	38.5	27.36	22.88	No	Yes		
chr4	76572212	104	24.36	24.79	Yes	No	Exon:1549716	
chr1	32418944	16.5	21.57	29.20	No	Yes		
chr4	6764254	27.5	20.26	36.47	No	No		
chrX	126448682	23	27.14	23.01	Yes	No		CpG Shore
chr4	153021987	14	23.95	25.00	Yes	Yes	TSS:1307785	CpG Shore
chr7	110541180	13.5	32.97	21.54	No	No	Intron:1309461	CpG:_35
chr7	110541188	13.5	32.97	21.54	No	Yes	Intron:1309461	CpG:_35
chr15	40073138	43	-20.49	-34.58	Yes	Yes	Intron:1564996	
chr14	248513	76	-22.58	-26.84	No	No		
chr15	63764801	20.5	-22.83	-26.47	Yes	No		

chr5	136575722	43	20.23	36.45	No	No	Exon:1304573	
chr1	83500116	23	21.67	28.79	Yes	No	Intron:1311009 | TSS:1306219	CpG Shore
chr19	10279242	21	27.96	22.59	Yes	No		
chr18	79829171	40.5	-56.86	-20.83	No	No		
chr10	106575521	98	20.58	33.63	No	No		
chr15	105951839	19	21.18	30.41	No	No		
chr1	210601763	73.5	-20.95	-31.42	No	No		
chr6	1710991	81	23.61	25.19	No	Yes	Exon:1304994 | TSS:1311578	
chr17	83633035	33	-29.90	-22.05	Yes	Yes		
chr1	18343269	20	45.03	20.88	Yes	No		
chr7	1677342	23.5	-21.67	-28.63	Yes	Yes	Intron:1307124	
chr1	54253050	30	26.26	23.29	Yes	No	TSS:70949	CpG Shore
chr12	40467808	14	-22.62	-26.67	No	No	TSS:1565800	
chr7	113718685	26.5	20.71	32.65	No	No	Intron:1307276	
chr20	4281833	28	-26.11	-23.33	Yes	Yes	Intron:1307063 | 1303282	
chr1	260125971	17	-36.96	-21.15	No	No	Intron:1307427	
chr17	21532358	33	-20.47	-33.97	Yes	No		
chr3	7920606	15	21.90	27.92	Yes	Yes	Intron:620648 | 1564504	CpG Shore
chr2	200965777	18	20.98	31.06	Yes	No	Intron:1306526	
chr14	9232966	14.5	-33.33	-21.43	Yes	No		CpG Shore
chr11	35043951	28.5	26.89	22.99	Yes	Yes	Intron:2959	
chr13	104279242	26.5	-25.00	-23.93	Yes	Yes	Intron:1303236	
chr9	108336285	31.5	28.18	22.38	No	Yes		
chr13	28402549	29	-66.56	-20.75	No	No		
chr8	50161893	20	-27.27	-22.73	No	No		
chr3	158779419	94.5	29.84	21.91	No	No	Exon:1309093	
chr5	64716565	17	-35.15	-21.25	No	Yes	Intron:61882	CpG Shore
chr3	159334634	17.5	28.47	22.22	Yes	Yes		
chr15	14479489	17.5	26.67	23.03	No	Yes		

chr12	26853520	28.5	-34.76	-21.25	No	No		
chr20	4791127	18	22.01	27.45	Yes	Yes	Intron:1307063 | 3817 | TSS:3426	
chr5	144633020	17.5	-24.44	-24.31	Yes	Yes		
chr19	52511859	136.5	-27.57	-22.55	No	Yes		
chr15	37129565	13	32.58	21.43	No	Yes	Intron:2610	
chr15	79713814	19.5	37.41	21.05	No	Yes	Intron:1307452	
chr5	160186653	15	-20.83	-31.25	Yes	Yes	Intron:1307204	CpG Shore
chr5	165738790	23	29.38	21.95	Yes	Yes	Intron:1585063	
chr1	191052304	63	-30.96	-21.64	No	No		
chr10	63999444	27	21.28	29.46	Yes	Yes		
chr3	144213150	21.5	23.55	25.00	Yes	No	Intron:1560257	
chr11	79794747	12	-22.92	-25.84	Yes	Yes		
chrX	111135074	21.5	27.60	22.48	No	Yes		
chr13	28444681	29.5	22.92	25.83	No	No		
chr1	120688874	22.5	22.36	26.67	Yes	No	Closest gene:1308000	
chr6	108261820	18	20.67	31.82	Yes	Yes		
chr12	40464846	14	-20.28	-34.12	No	No	TSS:1565800	CpG Shore
chr3	159319079	16.5	23.31	25.19	No	Yes		
chr17	12541226	85.5	23.87	24.73	No	No	Intron:1565218	CpG:_26
chr7	9670419	18	43.95	20.75	No	Yes	Intron:619751	
chr5	144251451	39	40.28	20.83	No	No	Exon:1566306 | TES:1562679	
chr1	94557994	15	25.96	23.16	Yes	No	Closest gene:1598667	
chr7	67043734	16.5	25.32	23.50	No	Yes	TES:1359574	CpG Shore
chrX	22729440	21.5	-20.83	-30.95	Yes	Yes	Intron:1562735	CpG:_132
chr3	148807409	25.5	27.59	22.40	No	Yes		
chr5	169341231	25	24.52	24.09	Yes	No	Exon:1303132 | TSS:1598206	
chr5	98970033	24	-40.00	-20.83	Yes	Yes		
chr3	47074678	24	23.50	25.00	Yes	No	Intron:1564504	
chr7	113859591	21.5	20.16	34.72	Yes	Yes	Intron:2323632	CpG Shore

chr1	99745843	17.5	22.82	25.82	Yes	No	Intron:621824	CpG Shore
chr5	137306908	28.5	21.75	27.62	Yes	Yes	Closest gene:3946	
chr5	170390119	23	21.83	27.38	Yes	Yes		
chr12	34631621	93.5	26.45	22.91	No	No	Exon:1311873	CpG:_52
chr15	1272446	19	-26.39	-22.92	Yes	Yes	Intron:1596707	
chr16	81073291	16.5	-24.77	-23.81	Yes	Yes		
chr1	79935264	26.5	-33.06	-21.30	No	Yes		
chr9	84613494	32	-27.26	-22.50	Yes	Yes	Intron:1309503	CpG Shore
chr18	63128481	40.5	21.51	28.17	Yes	Yes		
chr10	53300190	106.5	22.04	27.06	No	No		
chr1	168076480	13.5	-76.15	-20.51	No	Yes	Intron:1588657	
chr1	85409462	15	22.22	26.67	No	Yes	Intron:2323306	CpG Shore
chr9	61854884	17	23.92	24.44	Yes	No		
chr1	205664945	49.5	23.56	24.90	No	No	Closest gene:628800	
chr7	114144791	13	-23.08	-25.21	Yes	Yes	Closest gene:1587008	
chr8	69192922	17.5	-42.11	-20.67	Yes	No	Intron:620080	
chr10	85861578	23	-23.91	-24.42	Yes	No	Intron:631328	
chr5	170881002	17	20.11	34.37	Yes	No	Intron:1307083	
chr18	63853047	30.5	20.65	31.25	No	No		
chr7	32451322	24.5	25.93	23.08	No	Yes		
chr9	13614707	55.5	27.09	22.49	No	Yes	Intron:1307308	
chr19	55303193	18.5	-22.82	-25.59	Yes	No	Intron:631359	
chr5	21798673	26	-32.95	-21.21	Yes	Yes		
chr18	282371	12	-21.28	-28.79	Yes	No		CpG Shore
chr18	6480754	20	51.90	20.48	No	No		
chr3	148133321	13	22.16	26.67	Yes	Yes		
chr20	4072612	11.5	21.74	27.27	No	Yes	Intron:1307063 | 2204 | TES:2231	
chr20	28179488	44	-20.41	-32.27	No	No	Intron:1307063 | 2324779	
chr12	21557689	19	21.86	27.13	Yes	Yes	Exon:69284	CpG:_59

chr18	259641	15.5	32.28	21.25	Yes	Yes		
chr7	111865170	23.5	-21.45	-27.92	Yes	Yes		
chr2	95254133	23	20.24	33.12	Yes	No		
chr5	98970027	24	-36.67	-20.83	No	Yes		
chr1	54253133	15	-35.29	-20.91	No	Yes	TSS:70949	CpG Shore
chr3	76354573	33	28.89	21.78	Yes	Yes	Intron:1564504	CpG:_75
chr3	162861615	19	24.21	24.00	No	No		
chr7	13186044	19.5	26.19	22.81	No	No		
chr20	14042697	21.5	-20.50	-31.47	Yes	Yes	Intron:1307063 | 1307993	
chr8	122707507	15	-29.37	-21.67	No	Yes		
chr5	17903265	20	35.42	20.87	Yes	Yes		
chr13	56766005	63	20.77	30.31	No	No		
chr20	4769157	33	-28.69	-21.79	Yes	No	Intron:1307063 | TES:3818	
chr2	133626928	14.5	32.31	21.18	Yes	Yes		
chr2	180077759	21	29.57	21.57	Yes	Yes	Exon:2194	CpG:_43
chr7	112640713	18	23.11	25.00	No	No	Intron:1305608	
chr1	253115105	23	-28.67	-21.77	No	No	Closest gene:1305481	
chr15	21724204	86.5	-26.65	-22.56	Yes	No		
chr18	28564866	16	-35.91	-20.83	No	Yes		CpG:_25
chr4	125425318	95.5	-22.88	-25.21	No	No	Exon:628685	
chr10	62471817	15.5	-27.58	-22.16	No	Yes		CpG:_142
chr4	157818363	38.5	23.53	24.59	No	No	Intron:1593191	
chr1	40276242	17	-24.21	-23.86	No	Yes		
chr12	46725986	43	20.83	29.95	No	No	Exon:1305954 | TES:1560935	CpG:_470
chr18	39771605	28	-33.74	-20.99	Yes	Yes		
chr5	159616686	16.5	-21.43	-27.78	No	Yes	TSS:3290	
chr1	156104024	22	28.61	21.74	No	Yes		
chr1	202057373	12	21.67	27.27	Yes	Yes		
chr1	188301477	36	-25.96	-22.82	No	No		

chr7	28581218	31.5	-31.09	-21.33	No	Yes		
chr1	128844071	42	30.76	21.39	No	No		CpG:_25
chr4	8355339	22.5	25.56	23.02	Yes	Yes		
chr4	125356145	19.5	-20.24	-32.22	Yes	Yes		
chr15	70495769	44.5	20.10	33.22	No	No		
chr5	151472220	16.5	35.00	20.83	Yes	No	Exon:1309334 | TSS:1304729	
chr4	175792171	41	-31.82	-21.11	No	Yes		
chr17	47214190	118	-20.25	-31.95	No	No	Intron:621422	
chr9	8604766	24	-53.33	-20.25	No	No		
chr1	94839548	16	37.36	20.63	No	Yes	Exon:621011 | TES:1309036	CpG Shore
chr10	8927383	16	-49.47	-20.28	No	No		
chr16	60328685	37	-21.75	-26.81	No	No	Exon:1308812	CpG Shore
chr1	79826151	26.5	-21.43	-27.71	No	No	TES:1309409	
chr4	165791903	27	-27.08	-22.22	No	Yes		
chr5	111309702	33.5	-22.22	-26.07	No	No	TSS:628611	
chr9	58021566	36.5	-55.24	-20.24	No	Yes		
chr2	117614360	18	25.89	22.73	Yes	Yes	Intron:2323146	
chr5	158870723	34	26.81	22.26	No	Yes		
chr5	39009054	17.5	-27.78	-21.90	Yes	Yes		
chr19	32252044	25	-53.39	-20.23	No	Yes	Closest gene:1588859	
chr11	32748150	27	22.06	26.29	No	No		
chr2	195473419	35.5	-27.38	-22.06	Yes	Yes	Intron:1359446	
chr12	22465481	54.5	-23.39	-24.45	No	No	Intron:631346	
chr7	95917645	22	-29.09	-21.53	Yes	Yes	Intron:1309883	
chr6	68138389	22	-27.27	-22.12	No	No		
chr16	79421101	26	48.64	20.24	No	Yes	Closest gene:1563576	
chrX	159848833	28	20.29	31.52	Yes	Yes		CpG:_61
chr8	39670970	17	21.05	28.57	Yes	No	TSS:1332836	
chr5	165234799	29	27.59	21.95	Yes	Yes	Intron:1562463 | TSS:621751	

chr20	8041377	28	20.83	29.41	Yes	Yes	Intron:1307063	
chr9	101536739	19	-30.16	-21.37	Yes	Yes	TSS:620391	
chr6	23836520	27.5	27.68	21.91	No	Yes	TSS:1311049	
chr5	168937041	29	24.07	23.78	No	Yes	Intron:1562703	
chr2	198940192	52	-20.90	-28.89	No	No		
chr3	58684692	17	21.82	26.58	No	Yes	Intron:621793 | 1564504	
chr7	128174975	20	21.12	28.21	No	Yes		CpG Shore
chr5	169669760	20.5	29.62	21.43	Yes	No	Intron:61828	
chr2	165198660	23.5	20.24	31.48	No	No		
chr13	26609176	19	20.83	29.17	Yes	Yes		
chrX	1642026	20.5	-22.22	-25.81	Yes	Yes		
chr2	139417872	12	-22.81	-25.00	Yes	Yes		
chr18	4029350	52.5	21.47	27.11	No	No	TSS:628776	
chrX	140329635	87.5	-21.29	-27.71	No	Yes		
chr6	1710992	71.5	-24.11	-23.63	No	No	Exon:1304994 | TSS:1311578	
chr19	47611690	14.5	-20.71	-29.41	Yes	Yes		CpG Shore
chr1	84295200	19.5	-21.15	-27.89	Yes	No	TES:1311713	CpG Shore
chr8	31446207	15.5	24.77	23.21	No	Yes		
chr10	55229424	110.5	21.07	28.03	No	No	Exon:1563261	
chr1	179505934	42.5	20.59	29.92	Yes	No	TSS:1560558 | 2322109	
chr6	137420689	23.5	34.00	20.71	Yes	No		CpG:_37
chr14	56371535	19	20.47	30.09	Yes	Yes		
chr17	77881727	24	34.07	20.71	Yes	No		
chr10	102650354	19	-22.71	-25.00	Yes	Yes		
chr1	96345491	17	21.82	26.27	No	No	Exon:2740 | TSS:1306764	
chr10	76238552	14	-21.48	-26.84	Yes	No		
chr5	98970030	24	-32.50	-20.83	Yes	Yes		
chr12	22400723	25	33.77	20.70	No	No	Intron:620305	
chr5	142550967	28	-23.53	-23.99	Yes	No	Intron:1305022	

chr6	5935911	30.5	22.96	24.75	No	No		
chrX	93192754	19	29.38	21.34	Yes	Yes	Exon:1560259	CpG:_100
chr6	55778808	23.5	-22.62	-25.00	No	No		
chr4	57922315	11.5	-21.02	-28.03	Yes	No	Intron:1566215	
chr7	141740197	21	-21.30	-27.27	Yes	Yes	TSS:1595784	
chr1	83369773	18	22.62	25.00	No	No	Exon:1562943 | TES:70953	CpG:_18
chr2	215379279	48.5	-34.49	-20.59	No	Yes	Intron:621218	
chr6	127590232	15.5	22.61	25.00	Yes	Yes		
chr9	33788326	14.5	-20.71	-29.05	Yes	No		
chr7	138229731	19	30.96	21.00	Yes	Yes		
chr12	39685454	45.5	20.89	28.41	Yes	No	Intron:1566317	
chr18	70528204	143.5	-21.86	-26.01	No	Yes		CpG Shore
chr16	44085822	22	22.83	24.76	Yes	Yes		
chr14	46831776	26	20.93	28.12	No	No	Intron:1306061	
chr19	19592578	15	31.87	20.83	No	Yes		
chrX	127309382	47.5	31.26	20.90	No	Yes		CpG:_74
chr2	35552399	22	24.55	23.09	Yes	Yes		
chr9	11061346	39.5	24.27	23.29	No	Yes	Exon:1562074 | TES:1305744	CpG:_118
chr2	203998294	60	-22.22	-25.21	No	No	Exon:628813	
chr5	171926495	28.5	20.91	28.13	No	Yes		
chr8	63061974	24	-25.10	-22.73	Yes	Yes		
chr5	162625859	30	-22.70	-24.94	Yes	Yes		
chr5	169341232	52.5	-20.64	-28.96	Yes	No	Exon:1303132 | TSS:1598206	
chr7	35246686	14.5	35.91	20.36	Yes	No		
chr7	10760279	23	23.21	24.07	Yes	Yes		
chr8	118836109	14.5	25.89	22.27	Yes	Yes	Intron:1597145	
chr13	81583838	12	28.21	21.43	Yes	No	Intron:2305	
chr1	90281605	24.5	21.10	27.30	Yes	No		
chr7	130183641	128	-25.52	-22.42	No	No		

chr14	105313987	20.5	-20.12	-30.45	No	Yes		
chr5	149779743	16.5	21.29	26.92	Yes	No		
chr1	203822261	16	21.73	25.96	No	Yes		CpG:_94
chr6	25154827	68.5	26.19	22.14	No	No		
chr3	7715731	24	21.71	25.93	Yes	Yes	Intron:1303144 | 1564504	
chr2	234838887	22.5	22.88	24.33	Yes	Yes		
chr2	125896468	16	-24.62	-22.92	Yes	Yes	Intron:1564291	
chr4	122278022	20	20.34	29.52	No	No	TSS:2664	CpG Shore
chr19	52806167	13.5	32.87	20.54	No	No	Intron:1308822	
chr19	55835105	40.5	26.02	22.12	Yes	No		
chr17	59066681	38	25.00	22.60	No	No	Intron:1561038	
chr8	45483458	29	21.58	25.99	Yes	No		
chr6	35083099	21.5	20.65	28.33	Yes	Yes		
chrX	25762643	33.5	26.95	21.67	Yes	Yes	Exon:1563642	CpG:_109
chr9	8905648	48.5	24.44	22.91	Yes	Yes		
chr17	40238765	26	-21.58	-25.89	No	Yes	Intron:1307146	
chr9	6751235	19	28.95	21.05	Yes	Yes	Intron:1306128	
chr4	13229661	25	-25.56	-22.22	Yes	Yes		
chr4	61408467	14	31.70	20.59	Yes	Yes	Intron:1308226	
chr1	96718336	82.5	-29.21	-21.02	No	No		
chr11	64101551	18	-27.88	-21.38	Yes	No	Intron:621261	
chr2	174854054	18.5	20.29	29.24	Yes	No		CpG Shore
chr5	169341276	62	20.97	27.21	Yes	No	Intron:1303132 | TSS:1598206	
chr15	32530804	25.5	-21.68	-25.62	Yes	Yes	Intron:1310471	CpG Shore
chr1	80254369	16.5	-25.60	-22.18	Yes	Yes		
chr1	174226259	16.5	25.90	22.03	Yes	Yes		
chr1	82524928	76	24.24	22.96	No	No	TSS:1304682 | 1306080	
chr12	15453880	33	20.53	28.47	Yes	Yes		
chr5	111309419	18	30.05	20.81	Yes	Yes	TSS:628611	

chr19	38413541	36.5	-25.62	-22.15	No	Yes		CpG Shore
chr4	118431905	17.5	-21.49	-25.89	Yes	No		
chr15	102991882	49.5	25.91	22.00	Yes	Yes		
chr3	10032269	16	27.54	21.43	Yes	Yes	Intron:628863 | 1564504	CpG Shore
chr5	169317627	13.5	27.53	21.43	Yes	Yes	Intron:1303132	CpG Shore
chr19	49499676	21.5	26.79	21.66	Yes	Yes	Intron:619745	
chr7	137390538	13.5	26.67	21.67	Yes	Yes	Intron:2248 | TES:1308685	
chr6	95012565	29	-24.90	-22.55	No	No	Intron:1308036	
chr5	166993274	23.5	-20.24	-29.22	Yes	Yes		
chr14	18883499	60.5	26.68	21.62	No	Yes		
chr7	30943288	22.5	32.12	20.43	Yes	Yes	Intron:1308216	
chrX	82270105	17	22.32	24.62	Yes	Yes		CpG:_51
chr6	8849603	55.5	-22.19	-24.98	Yes	Yes	Intron:1310453	
chr1	213891785	21.5	35.31	20.05	Yes	Yes		
chr10	93650520	20	-25.60	-22.06	Yes	Yes		
chr2	140575873	18	30.17	20.70	Yes	Yes	Exon:1308177	
chr2	17162836	17	23.33	23.44	Yes	No		
chr3	154407348	39.5	20.29	28.81	Yes	No	TSS:1305296	
chr1	39514903	58	20.51	28.11	No	No		
chr4	155626421	64.5	22.85	23.91	Yes	Yes	Exon:1587001	
chr14	33298477	20.5	26.06	21.79	No	Yes		
chr3	106947424	32.5	-23.94	-22.95	Yes	No	Exon:2269 | TES:2660	
chr2	28274088	37.5	28.62	20.97	Yes	No		
chr14	36511152	16	-24.75	-22.44	No	Yes		
chr4	5961400	27	-20.59	-27.64	Yes	No		
chr10	19189767	25	29.46	20.77	No	No	Exon:1307421	
chr13	106256125	28	29.42	20.77	Yes	No		
chr8	62255306	17	23.58	23.08	Yes	Yes		
chr5	139766842	30.5	31.03	20.45	Yes	No	Intron:1309996	

chr2	157464052	70	-20.16	-28.84	No	No	Exon:1586171	
chrX	121209686	33.5	25.43	21.97	Yes	Yes	TSS:1565392	CpG:_152
chr20	4945416	46.5	-23.73	-23.03	Yes	No	Intron:1307063 | 2373	
chr2	94707657	25	20.52	27.76	Yes	Yes		
chr5	138447994	19	-28.95	-20.83	Yes	Yes	Intron:68414	
chr6	130303494	27.5	-31.08	-20.42	Yes	Yes		
chr13	70400564	19	-20.87	-26.75	No	Yes		CpG Shore
chr5	163902052	27	22.20	24.60	Yes	Yes		
chr3	7268735	35.5	-20.74	-27.17	Yes	No	Intron:1564504	
chr2	22846879	26	-20.72	-27.24	No	Yes		
chr6	144467436	35	-25.70	-21.80	Yes	Yes	Intron:61904	
chr16	32596937	35	23.81	22.86	Yes	No		
chr3	61000071	18.5	28.10	21.01	No	Yes	Intron:1564504	
chrX	138566000	22.5	23.81	22.86	Yes	Yes		CpG:_143
chr17	26604851	26.5	-20.05	-28.90	Yes	No		
chr3	135346039	38	22.77	23.73	Yes	Yes		
chr6	125117607	18.5	21.74	25.00	Yes	Yes	Intron:1311756	
chr7	74463189	17.5	-25.05	-22.06	Yes	Yes	Intron:628703	
chr10	82207657	19	22.86	23.61	Yes	Yes		
chr9	50027019	16.5	21.11	26.12	Yes	Yes		
chr5	149809586	19	-21.05	-26.25	Yes	Yes	Intron:1359410	CpG:_31
chr20	13717673	70	-26.11	-21.57	No	Yes	Intron:1307063	
chr5	63508217	79	-24.36	-22.46	No	Yes	Intron:619864	
chr1	192826739	39	-26.03	-21.62	No	No		
chr7	139462014	51.5	20.15	28.53	No	No	Intron:2319726 | 735164 | TES:2324822	
chr20	53283885	35	22.56	23.82	Yes	No	Intron:2733	
chr4	180154281	36	-21.88	-24.82	No	No	TSS:2960	
chr7	118683943	13.5	25.82	21.67	Yes	No	TES:68944	
chrX	93192660	19	26.83	21.34	Yes	No	Exon:1560259	CpG:_100

chr5	15513470	17	32.21	20.09	Yes	No	Intron:1560182	
chr5	169341275	29	-21.93	-24.65	Yes	No	Intron:1303132 | TSS:1598206	
chr13	67241229	37.5	21.24	25.67	No	Yes		
chr12	21948939	31.5	20.30	27.78	Yes	No		CpG:_23
chr13	46274818	14	-20.83	-26.62	Yes	Yes	Intron:3554 | TSS:1305304	
chr7	73231575	34	21.20	25.71	Yes	No		
chrX	86399436	17	-20.62	-27.02	No	Yes		
chr16	8192830	20	22.58	23.75	Yes	Yes		
chr9	36515610	35	-21.16	-25.75	No	No		
chr8	110258975	94	-22.61	-23.70	No	No		
chr2	26052773	32	26.04	21.48	Yes	No	Intron:2321734	
chr6	135607100	42.5	22.84	23.45	Yes	No	Intron:1563395	
chr6	137304128	14	24.73	22.17	Yes	Yes		
chr12	34220933	20.5	-20.24	-27.78	Yes	No	Intron:1561461	
chr13	94865313	13	-26.01	-21.43	No	No	Intron:1562635	
chr2	46522084	20	20.83	26.39	Yes	Yes		
chr3	42572675	27	24.73	22.12	Yes	No	Intron:1307777 | 1564504	CpG Shore
chrX	110931593	26.5	22.53	23.68	Yes	No		
chr12	33000930	94.5	-24.88	-22.04	No	No		CpG:_31
chr4	80520201	53.5	-24.75	-22.09	Yes	No	TSS:1587253 | TES:1310001	CpG Shore
chr13	13170206	21.5	-24.44	-22.22	Yes	Yes	Closest gene:1304661	
chr1	96276041	59	26.46	21.36	Yes	Yes	Closest gene:620398	
chr3	162607507	62	-24.26	-22.25	No	No		
chr10	71207886	14	-26.67	-21.25	No	No		
chrX	40365085	48	-22.99	-23.17	Yes	Yes		CpG:_77
chr18	79750531	18	-26.27	-21.36	Yes	Yes		
chr18	28848693	33	-29.81	-20.30	Yes	Yes		
chr8	124943219	32.5	27.78	20.81	Yes	Yes		CpG Shore
chr14	10232725	13	24.71	21.97	Yes	Yes		

chr14	90356186	15.5	-20.71	-26.37	Yes	Yes		
chr1	263225061	15	21.43	25.00	Yes	Yes	Exon:1310668	
chr5	137778021	19.5	-20.83	-26.09	Yes	No		
chr20	8131866	43	24.56	22.01	No	No	Intron:1307063	
chr10	37160159	20	-20.56	-26.67	Yes	No	Exon:1308356	
chr3	153812144	17.5	29.33	20.34	Yes	Yes	Intron:1307316	
chr5	166203918	46	26.06	21.37	No	No	TSS:2383	
chr13	48260236	95	-26.01	-21.38	No	No	Exon:1310687	
chr10	62985670	18	25.65	21.43	Yes	Yes		CpG Shore
chr4	80727576	17.5	-21.05	-25.49	Yes	Yes		
chr3	85642242	32.5	-21.83	-24.24	Yes	Yes	Intron:1564504	
chr13	99319682	43	-22.74	-23.24	No	No		
chr14	3879090	39	-20.81	-26.07	No	No	Closest gene:620726	CpG:_21
chr3	58310366	27.5	25.24	21.52	No	No	Intron:1564504	
chr13	95905744	22.5	-27.86	-20.66	Yes	Yes		
chr6	32749544	44.5	22.45	23.43	No	No		
chr16	19203904	15	23.72	22.32	Yes	Yes		CpG Shore
chr5	152072584	34	-27.65	-20.68	No	Yes	Intron:1310031	CpG:_129
chr6	13984064	19	22.27	23.53	Yes	Yes		
chr16	61296716	19	20.59	26.32	Yes	Yes		
chr10	86202925	21.5	24.24	22.03	No	No	Intron:1309742	
chrX	61317871	13.5	20.14	27.27	Yes	Yes		
chr4	165894196	15	-22.73	-23.11	No	Yes		
chr14	77352308	25	21.58	24.40	Yes	Yes		
chr14	6859753	21.5	21.49	24.54	No	Yes		
chrX	21294166	32	-20.05	-27.31	Yes	Yes	Exon:1561300	CpG:_91
chr4	37965564	17	29.04	20.24	No	No	Intron:1566201	
chr13	68629597	29.5	24.58	21.78	Yes	No		
chr2	182392632	17	24.89	21.67	No	Yes	Intron:1310250	

chr10	87687032	20.5	20.97	25.27	Yes	No		
chr18	72269810	34.5	21.90	23.86	Yes	Yes		CpG Shore
chr5	47853064	20	-29.64	-20.07	Yes	Yes		
chr13	46147819	18	22.22	23.53	Yes	Yes	Intron:1564395	
chrX	94686096	18.5	26.00	21.15	Yes	Yes		
chr8	47804149	33	27.27	20.71	No	Yes	Exon:1586165	
chrX	43609261	19	22.56	23.18	Yes	No		
chr1	79043136	46.5	-24.99	-21.57	Yes	No	Exon:1309505	
chrX	31244266	17.5	-21.88	-23.81	Yes	No		CpG:_55
chr1	89795258	32.5	20.60	26.04	Yes	No	Exon:1563363	
chrX	87123244	73.5	22.94	22.83	No	No		
chr6	133168332	13.5	21.37	24.73	Yes	Yes	Exon:1308104	CpG Shore
chr19	44472292	20.5	20.70	25.74	Yes	Yes		
chrX	113734769	70	23.83	22.08	No	Yes		
chr16	3177697	18	22.51	23.08	Yes	Yes	Intron:708372	
chr7	56296054	16	27.94	20.39	No	Yes		
chr12	11670396	20	-20.12	-26.84	Yes	Yes		CpG:_262
chrX	58707903	50	20.28	26.50	Yes	No		CpG:_53
chr2	155962084	14.5	-23.64	-22.17	Yes	Yes	Intron:1308217	
chr7	139479859	26.5	23.23	22.43	Yes	No	Intron:2319726 | 735164	
chr14	49345827	31	23.33	22.32	Yes	No		
chr8	62852137	30.5	-21.69	-23.87	Yes	Yes		
chr7	83236744	29.5	-20.24	-26.52	Yes	Yes		
chr8	63508987	35	-28.33	-20.24	Yes	Yes	TES:3337	CpG:_42
chrX	39211791	39	26.23	20.91	Yes	Yes		
chr18	47171074	60	-21.57	-24.00	No	Yes		
chr2	179452835	45.5	-21.18	-24.73	Yes	No	Intron:1564496	
chr1	122606351	21	27.38	20.48	Yes	Yes		
chr19	25348782	15.5	-27.38	-20.45	Yes	No		

chr2	242432441	22	26.36	20.83	Yes	Yes		
chr10	53726477	26.5	-22.14	-23.33	No	No	Exon:1309576	CpG:_19
chr18	28958310	59.5	-24.27	-21.67	No	No	Exon:2322910	
chr3	108594756	14.5	-25.00	-21.43	No	Yes	Intron:1564504 | TSS:1561554	
chr1	253115106	33	21.43	24.14	No	No	Closest gene:1305481	
chr14	7578208	15	24.43	21.57	No	No		
chr4	108163257	108.5	23.00	22.50	No	No		
chr1	173816595	33.5	27.98	20.22	Yes	No	Intron:1309000	
chr3	7950119	23.5	20.24	26.24	No	No	Intron:1564114 | 1564504	
chr1	109563012	27	-26.71	-20.63	Yes	No		
chr14	82825580	14.5	20.24	26.19	Yes	Yes		
chr8	45062065	41.5	-20.10	-26.56	No	No	Intron:619914	
chr5	141453013	13.5	-28.21	-20.13	Yes	No		
chr9	7944260	20	21.05	24.71	No	No	TSS:1308727	
chr16	60311301	26.5	23.94	21.74	No	Yes	Intron:1308812	
chr5	73278368	29.5	20.28	26.10	Yes	Yes	Intron:1562958	
chr17	52459740	14	23.08	22.35	Yes	No	Intron:1308182	
chr5	122764089	17	20.39	25.78	Yes	Yes		
chrX	58708140	23	26.11	20.82	Yes	No		CpG:_53
chr9	105475382	14	21.43	23.92	Yes	No	Intron:1308319	
chr8	20204241	85.5	20.67	25.13	No	No	Intron:3279	
chr12	23454994	23	21.17	24.38	Yes	No		
chr11	57755821	45	20.39	25.69	No	Yes	Intron:1559942	
chr10	13756337	36	-23.14	-22.22	No	Yes	Exon:620191 | TES:1559653	CpG Shore
chr19	55939602	14	24.57	21.43	Yes	Yes	Intron:1306192	
chrX	37867456	15	24.18	21.52	Yes	Yes		
chr12	32192872	17.5	22.44	22.75	No	Yes		
chr1	138876487	23	-25.00	-21.13	Yes	Yes	Intron:1305155	
chr14	94207480	31	-27.01	-20.36	No	No		

chr16	52235999	37	-24.32	-21.43	No	Yes		
chr10	87041095	20.5	25.42	20.92	No	Yes	Intron:727791	
chr1	42952755	32.5	26.61	20.51	Yes	Yes		
chr12	8681506	16	-22.38	-22.73	Yes	Yes		
chr10	69946000	85.5	-20.41	-25.36	No	No		
chr13	102951384	15	21.82	23.21	Yes	No		
chr6	7209043	35	22.56	22.54	Yes	Yes	Intron:71071	
chr15	97124718	18.5	23.68	21.67	No	Yes		
chr6	10844147	28	-20.10	-25.89	Yes	No	Intron:1306211	
chr1	205623038	36.5	23.83	21.53	Yes	Yes		
chr1	210697300	30.5	-22.67	-22.40	Yes	No		
chr12	19676000	156.5	25.51	20.79	No	Yes	Intron:3270 | TES:1305095	
chr5	149072894	15.5	-21.43	-23.53	Yes	Yes		CpG:_38
chr7	141750041	15	-26.90	-20.24	Yes	No	Intron:1595784	
chr10	91526945	31.5	25.00	21.00	Yes	Yes	Closest gene:61983	
chr1	73678902	13.5	-23.33	-21.79	No	No	Closest gene:1359582	
chr5	59155769	27	22.22	22.69	No	Yes	TSS:1560943	CpG Shore
chr9	85419674	31.5	20.63	24.92	Yes	Yes		
chr11	50990898	25	-22.51	-22.38	No	No		
chr12	40280716	19.5	21.90	23.02	Yes	Yes	Intron:1562674	
chr9	37717089	16	21.85	23.04	No	Yes		
chr7	125887185	22	23.70	21.48	Yes	No		
chr1	35215041	25.5	21.65	23.23	Yes	Yes	Intron:1310945	
chr17	14055743	35.5	21.90	22.90	Yes	Yes		
chr13	28402548	33.5	21.84	22.95	No	No		
chr15	39626563	30.5	20.22	25.15	No	Yes		
chr15	59176917	56.5	24.73	20.96	No	Yes		
chr20	16635974	33.5	-24.17	-21.21	No	No	Intron:1307063	
chr7	56350535	41	26.55	20.21	No	No	Intron:1305439	

chr10	97730851	18	22.58	22.22	Yes	Yes		
chr16	63873804	15	23.29	21.67	Yes	Yes		
chr12	14477332	20.5	-21.88	-22.81	Yes	No	Exon:1309549	
chrX	118752232	30.5	-25.00	-20.81	Yes	Yes		
chr12	39380810	41	-21.34	-23.40	Yes	No		
chr4	6321919	47.5	-23.41	-21.52	Yes	Yes		
chr5	146157817	20	-22.22	-22.38	Yes	Yes	Exon:621734	
chr2	180061854	15.5	-25.00	-20.78	No	Yes	TES:3162	
chr12	31439117	30.5	-22.82	-21.93	Yes	Yes		
chr7	98961645	41	22.22	22.42	No	Yes	TES:3130	
chr10	103258267	19	21.05	23.64	Yes	No		
chr13	10209902	19	-20.51	-24.62	Yes	No		
chr13	71165897	26.5	22.04	22.56	Yes	Yes	Intron:1565760	
chr3	154219813	20	24.29	21.05	Yes	Yes	TES:1305196	
chr15	65538779	18	22.08	22.51	No	Yes		
chr4	135155701	19.5	21.05	23.61	Yes	Yes	TSS:1307077	CpG:_49
chr11	29513662	15.5	20.21	25.00	Yes	No	Intron:1565091	
chr16	69283098	14.5	25.00	20.67	Yes	Yes		
chr1	245214398	22	22.77	21.85	Yes	Yes		
chr19	51844573	28	20.75	24.05	No	No	TSS:621315	CpG Shore
chr18	24357684	29.5	-21.98	-22.51	No	Yes	Intron:1305273	
chr2	122607528	29	-26.21	-20.12	Yes	Yes		
chr18	62727654	23.5	21.76	22.73	No	Yes	Intron:1565629	
chr1	185715880	31.5	25.02	20.56	No	No	Exon:1359537	
chr3	68385276	19.5	-20.63	-24.21	Yes	Yes	Intron:1564504	
chr10	85424326	12.5	-23.81	-21.15	Yes	Yes	Intron:708436	
chr8	18929799	18.5	-21.73	-22.73	Yes	Yes		
chr19	19275307	14.5	-21.14	-23.38	Yes	Yes		CpG Shore
chr17	87525629	14	-21.43	-23.08	Yes	No	Intron:1591781	

chr1	210601762	64	21.88	22.53	No	Yes		
chr4	119653022	25.5	20.39	24.58	Yes	No		
chr3	118205942	28	-22.76	-21.75	Yes	No	Intron:2184	CpG Shore
chrX	156277299	47.5	21.50	22.89	Yes	Yes		
chr3	161484613	14.5	21.46	22.92	No	Yes		
chr16	55802029	19	-21.05	-23.45	Yes	Yes		
chr12	44051745	17.5	25.00	20.53	Yes	Yes	TSS:69359	
chr3	156036031	21.5	-20.16	-24.92	No	No		CpG:_70
chr11	62054774	14.5	-20.24	-24.73	No	Yes	Intron:1566080	
chr5	137681062	14.5	23.63	21.15	Yes	Yes		
chr6	87340788	18	-22.22	-22.06	No	Yes		
chrX	40334504	21	25.38	20.24	Yes	Yes		CpG:_25
chr6	133200546	65	20.96	23.38	No	No	TES:1303247 | 1308278	
chr8	18377884	16	22.32	21.90	No	Yes		
chr6	75314192	19.5	20.55	23.92	Yes	Yes	Intron:1306199	
chrX	79391072	31	21.05	23.25	Yes	No		
chr1	44214957	23	22.50	21.74	Yes	Yes		
chr15	59015485	34	23.33	21.22	Yes	Yes	Intron:1563207	
chr17	16261179	29.5	24.21	20.77	No	Yes		
chr20	29547422	22.5	22.62	21.67	Yes	No	Intron:1307063 | 1305994	CpG:_31
chr4	173893949	47.5	21.00	23.24	Yes	No	TSS:1306456	
chr13	95281681	39	-21.05	-23.16	Yes	Yes		
chr8	71869278	28.5	21.98	22.21	Yes	Yes	Intron:1565416	
chr20	45906532	42.5	-21.58	-22.48	No	No		
chr1	179301370	44	22.15	21.99	Yes	Yes		
chr6	123468678	15	-20.78	-23.50	No	Yes	Intron:1305711	
chr1	120276962	18.5	20.29	24.14	Yes	Yes		
chr11	34337876	20.5	-24.13	-20.71	Yes	Yes	Intron:1308016	CpG:_41
chr9	106649004	19	20.83	23.36	Yes	Yes		

chr7	79238247	26	22.41	21.72	Yes	Yes		
chrX	24208248	33.5	-23.33	-21.13	No	No	TSS:1303258	CpG:_104
chr17	58293468	29	20.98	23.15	Yes	Yes		
chr11	84401138	22.5	-22.22	-21.86	Yes	No		CpG:_30
chr8	101157281	66	21.98	22.10	No	No	TSS:1311223	
chr1	96783419	21	21.37	22.73	Yes	No	Intron:1306610	
chr15	18650939	56.5	20.82	23.37	No	No		
chr8	120090037	29	-20.26	-24.14	Yes	Yes		
chr20	6695087	19	23.33	21.05	Yes	No	Intron:1307063	
chr3	15213311	16.5	-25.00	-20.20	Yes	Yes	Intron:1306174 | 1564504	CpG Shore
chr18	64301456	19.5	20.51	23.72	Yes	No		
chr17	15731526	33	-24.84	-20.29	No	No	Closest gene:621755	
chr16	81472075	18.5	-22.97	-21.32	Yes	No	TSS:619782	
chr1	35926402	20	20.82	23.31	Yes	Yes	Exon:620546	
chr3	57953980	33	-20.11	-24.24	No	No	Intron:1564504	
chr4	18412806	20	23.46	20.92	Yes	No		
chr8	124497494	24.5	23.62	20.83	Yes	Yes	Intron:3637	
chr12	38197783	32	21.60	22.24	Yes	Yes		
chr16	81166078	81	21.78	22.14	No	Yes		
chr18	30530716	16	-22.73	-21.43	Yes	Yes		
chr6	87562316	29.5	22.22	21.71	No	Yes		
chr3	24247539	51	21.53	22.22	No	No	Intron:1564504	
chr1	199701602	14	-23.08	-21.08	Yes	Yes	Intron:1308241	
chr6	25261000	114	22.44	21.43	No	No		
chr10	98933400	21.5	-22.73	-21.36	No	Yes		
chr4	169468598	55.5	-22.12	-21.72	No	Yes		
chr7	22194074	23.5	-22.34	-21.48	No	No		
chr3	5011029	58.5	-21.67	-22.11	Yes	Yes	Intron:1564504	
chr2	165169052	14.5	20.83	23.08	Yes	Yes		

chr1	132100546	16.5	23.53	20.67	Yes	No		
chr8	69666750	23.5	21.67	21.92	Yes	Yes	Intron:1565748 | TES:1306268	
chr11	63017965	19	23.32	20.78	No	Yes		
chr6	7209044	32	22.48	21.33	Yes	Yes	Intron:71071	
chr2	94424601	56	22.47	21.31	Yes	No		
chr20	53048340	14.5	22.22	21.43	Yes	Yes		
chr7	93436251	18.5	-20.09	-23.74	Yes	Yes		
chr17	94777534	28	-22.29	-21.38	Yes	Yes	Closest gene:1563437	
chr3	114984070	31	21.45	21.99	Yes	Yes	Intron:70888	CpG Shore
chr10	104712340	34.5	-22.90	-20.93	No	No		
chrX	2970475	31.5	-20.11	-23.59	Yes	Yes	Intron:1562042	CpG:_145
chr4	18180298	20.5	22.61	21.09	Yes	Yes		
chr10	67131198	22	-21.43	-22.03	Yes	Yes		
chr5	78152553	16.5	21.18	22.22	No	Yes		
chr5	65558616	71.5	21.46	21.87	Yes	No		
chr10	50055160	13	-23.08	-20.77	No	Yes		
chr7	56060457	78	22.37	21.18	Yes	Yes	TSS:1564883	
chr5	64716363	35.5	-21.89	-21.47	Yes	Yes	Intron:61882	CpG Shore
chr13	81366739	16	20.24	23.33	Yes	Yes	TES:1304715	
chr7	119851577	28	-21.06	-22.25	Yes	No		
chr7	122400922	16	23.33	20.54	Yes	Yes		
chr20	27397073	48	24.07	20.08	Yes	Yes	Intron:1307063 | TSS:1308568	CpG Shore
chrX	55033015	23	20.81	22.67	Yes	No	TSS:2324133	CpG Shore
chr8	103463426	14.5	23.33	20.51	No	Yes	Closest gene:1309140	
chr1	97398118	17	22.22	21.25	Yes	No		
chr2	32297012	53.5	20.46	23.02	Yes	Yes		
chr2	236252313	22.5	20.71	22.70	Yes	No		
chr1	202630616	54.5	22.38	21.06	No	No	Intron:621511	CpG Shore
chr19	24732146	34.5	-23.06	-20.69	Yes	Yes		CpG Shore

chr7	126454885	34	21.43	21.87	Yes	Yes		
chr1	86656423	24.5	20.87	22.43	No	Yes	TSS:1589941 | TES:2727	
chr12	30334493	20.5	20.11	23.33	Yes	No	Intron:1583824	
chrX	124685010	16	20.83	22.49	Yes	Yes		
chr11	61815201	21	23.06	20.63	Yes	Yes		
chr5	6380776	32.5	23.08	20.59	Yes	No		
chr1	252721352	35	-20.83	-22.42	No	Yes		
chr5	64613964	29	-20.36	-23.03	Yes	No		
chr1	143614211	19.5	-22.96	-20.63	Yes	Yes		
chr10	55131771	34.5	-22.22	-21.11	No	Yes	Intron:619809	CpG Shore
chr3	5797960	114.5	22.52	20.87	No	Yes	Intron:1307382 | 1564504 | TES:1564893	
chrX	26655490	22	-23.08	-20.49	No	Yes	TSS:2293497	CpG:_19
chr16	19102148	31	-21.63	-21.51	Yes	Yes	Exon:2321347	
chr19	26171191	20	20.54	22.73	Yes	Yes		
chr14	62496572	22	-20.47	-22.77	Yes	Yes		
chr2	211852515	13.5	23.33	20.33	Yes	Yes	Exon:61958	
chr9	47028156	33	20.49	22.71	Yes	Yes		
chr4	78039175	15	-23.33	-20.24	No	Yes	Intron:3197	CpG Shore
chr6	96935698	26.5	21.43	21.63	Yes	Yes		
chr15	102515555	13	22.73	20.61	Yes	Yes	Intron:1593308	
chr4	6058124	16.5	23.33	20.18	Yes	Yes		CpG Shore
chr4	38619501	15	-20.22	-22.92	Yes	No	Closest gene:1561223	
chr16	48542733	19.5	20.81	22.22	No	Yes		
chr8	112915439	52	-20.21	-22.88	No	No	TSS:628620	
chr7	107306757	17.5	21.02	21.90	Yes	Yes		
chrX	160445578	31	20.37	22.66	No	Yes		CpG:_41
chr16	50175093	16	-21.43	-21.43	No	Yes		
chr13	91763784	58	21.08	21.77	Yes	Yes	Intron:1562609	
chr9	63093164	71.5	20.61	22.30	No	Yes	TES:2323711	

chr9	34946777	29.5	20.94	21.91	No	Yes		
chr12	35708198	29.5	-21.67	-21.25	No	Yes		CpG Shore
chr17	11590069	26.5	20.37	22.56	No	No	Intron:3213	
chrX	20321817	28.5	20.97	21.84	Yes	Yes		
chr1	166370415	29	-22.73	-20.45	Yes	Yes		
chr5	146148174	39.5	21.71	21.17	Yes	Yes	Intron:1563297	CpG:_18
chr5	150933671	34	-22.46	-20.62	Yes	No		CpG Shore
chr10	16816085	180.5	-22.63	-20.48	Yes	No	Exon:1585015	
chr10	17385019	29	21.51	21.33	Yes	No	Intron:3779	
chr7	140737694	12	-21.08	-21.67	Yes	No		
chr20	45166667	37	-21.67	-21.16	No	Yes		
chr6	3885756	16	-22.86	-20.28	Yes	Yes	TSS:1303057	CpG Shore
chr2	218495993	22	-22.68	-20.43	Yes	Yes		
chr11	70698654	19	20.66	22.11	Yes	Yes	Closest gene:1308155	
chr16	12700971	18.5	21.49	21.21	Yes	No		
chr8	113383160	17.5	-20.48	-22.22	No	No	Exon:2223	CpG:_24
chr4	153004450	20.5	20.43	22.22	Yes	Yes	Intron:1307785	TES:1565700	
chr6	91772380	36	-22.84	-20.19	Yes	Yes	Intron:1359208	TSS:1306250	CpG Shore
chr20	11747383	19	21.66	21.05	No	Yes	Intron:1307063	TES:2033	
chr7	33020895	23.5	-20.61	-22.08	Yes	Yes		
chr10	57065615	21	22.25	20.56	No	Yes	Exon:1563179	CpG Shore
chrX	128496988	18	-22.22	-20.59	Yes	No	Intron:1565674	CpG:_88
chr5	141831313	27	-20.24	-22.37	Yes	Yes		
chr7	114335232	21.5	20.83	21.74	Yes	Yes	Closest gene:2323312	
chrX	127607178	19	20.51	22.08	Yes	Yes	Intron:1559905	CpG:_97
chrX	51304374	22	21.35	21.25	Yes	Yes	Exon:1559763	
chr2	174753086	16.5	-21.05	-21.43	Yes	Yes	Exon:2603	
chr15	24639677	15	22.22	20.51	Yes	No	Closest gene:1305705	CpG Shore
chr6	138097138	22	-20.97	-21.47	Yes	Yes		

chrX	48637293	32.5	20.59	21.89	Yes	No	Closest gene:1584894	CpG:_60
chr13	96125618	23.5	21.60	20.91	Yes	Yes	Intron:2932	
chr14	93881411	71.5	21.22	21.30	No	No	Exon:2320935	
chr17	74760374	47.5	-21.45	-21.01	Yes	No	TSS:61893	CpG Shore
chr11	71166522	36.5	-21.19	-21.32	Yes	Yes	TSS:620131	CpG Shore
chr12	44549932	67.5	20.14	22.28	Yes	Yes	Intron:1306556	
chr19	49714379	33	20.51	21.88	Yes	Yes	Intron:621844	
chr3	167394013	15.5	20.13	22.22	No	No		
chr18	26823847	44.5	-21.38	-21.04	Yes	Yes	Intron:1306047	
chr4	109975650	51	20.75	21.56	Yes	Yes		
chr13	75679282	32	22.25	20.23	Yes	Yes	Closest gene:1304620	
chr2	191501232	28.5	20.77	21.50	Yes	No	Exon:708413	
chr2	213689843	17	-21.77	-20.61	Yes	Yes	Exon:727806	
chr19	35149759	15.5	21.67	20.71	Yes	No	Intron:1589776	
chr20	28124556	36.5	20.44	21.78	No	Yes	Intron:1307063 | 621756 | 2324779	
chr3	159334530	17	21.69	20.61	Yes	Yes		
chr14	101770083	13.5	20.77	21.43	Yes	Yes		
chr7	138337791	42	21.43	20.77	Yes	Yes	Exon:2144	CpG Shore
chr9	10033057	26	-20.09	-22.03	Yes	Yes	Intron:1587048 | 1583221 | TES:2321693	
chr8	118052758	25	21.88	20.36	No	No		
chr2	102422719	15	21.67	20.56	No	Yes		
chr10	90490439	28.5	20.19	21.86	Yes	Yes	Intron:1308943 | TSS:1306172	CpG Shore
chr12	35613097	54.5	-20.03	-22.01	Yes	Yes	Closest gene:1562334	
chr9	58110904	19.5	20.63	21.43	Yes	Yes		
chr7	58564689	15.5	20.45	21.53	Yes	No		
chr10	72079987	24.5	21.57	20.49	No	Yes		CpG Shore
chr1	95812653	62.5	-21.91	-20.15	No	Yes	Intron:620054 | TES:727864	
chr10	5247411	29	-20.51	-21.43	Yes	Yes	Intron:1307081	
chr17	3915766	13.5	-21.67	-20.24	Yes	Yes		

chr5	171851288	28.5	20.17	21.56	Yes	Yes		
chr9	23003892	15	-20.24	-21.43	Yes	Yes	Exon:1308920	
chr12	37060670	22.5	21.76	20.03	No	Yes	Intron:1311820	
chr1	91429810	34	21.05	20.74	No	No		
chr10	84700013	29	21.56	20.23	Yes	Yes	Intron:708580	CpG Shore
chr1	80254402	18.5	-21.43	-20.35	Yes	No		
chr4	155633577	17.5	20.77	20.92	Yes	Yes	Exon:1587001	
chr8	34246828	47.5	20.01	21.53	Yes	No		
chr15	27805342	32.5	20.39	21.25	Yes	No		
chr13	48999734	53	-21.59	-20.07	No	No		
chr11	84849237	20	20.83	20.83	Yes	Yes		
chr5	12234360	35	-21.16	-20.51	Yes	Yes		
chr1	41197084	33.5	21.21	20.45	No	Yes		
chr4	38614025	36	20.62	21.00	Yes	Yes		
chr1	83488464	24	20.37	21.21	Yes	Yes	Intron:620722	CpG Shore
chr13	106706782	48.5	-20.38	-21.13	Yes	No		
chr1	101777124	16	21.30	20.18	Yes	Yes	Intron:1563167	
chr19	30707693	26	-20.38	-21.04	Yes	No	Closest gene:1560620	
chr14	100687832	45.5	21.36	20.07	Yes	Yes		
chr10	103589463	14	20.61	20.63	Yes	No		
chr9	106329915	21	-20.91	-20.29	Yes	Yes		
chr10	92163938	21	-20.45	-20.74	Yes	Yes	TES:1359211	CpG Shore
chr9	75178351	26	-20.91	-20.04	No	Yes		
chr13	95281702	26.5	-20.26	-20.63	No	Yes		
chr1	8729714	16	20.12	20.74	Yes	Yes		
chr11	68575050	39	20.64	20.24	Yes	Yes	Intron:621865	
chr12	38605007	24.5	20.51	20.25	No	Yes		
chr7	59520498	22.5	20.36	20.38	Yes	Yes		
chr8	108157624	15	20.39	20.33	No	No	Intron:1309958	

chr3	31045890	42.5	-20.09	-20.49	No	No	Intron:1564504	
chr8	35240191	14	20.40	20.18	Yes	Yes	Intron:708433	
chr3	1447148	17.5	20.34	20.10	Yes	Yes	Intron:1564504	
chr7	134229259	26	-20.05	-20.03	No	Yes	Intron:1359605	

Supplementary Table 3: Gene ontology terms associated with CpG sites showing consistent changes after depolarization. All gene ontology terms with p value < 0.05 that were overrepresented in the list of CpG sites that showed a consistent depolarization change. Abbreviations: BP – biological process; CC – cellular component; MF - molecular function.

Pathway	Ontology	GO:ID	Genes in pathway	Probes in pathway	Overlap pathway and test list	P value	FDR q value
regulation of ion transmembrane transporter activity	BP	GO:0032412	37	4334	15	2.90E-05	0.0563
regulation of glutamate receptor signaling pathway	BP	GO:1900449	17	2446	11	3.42E-05	0.0563
regulation of alpha-amino-3-hydroxy-5-methyl-4-isoxazole propionate selective glutamate receptor activity	BP	GO:2000311	14	2050	10	3.96E-05	0.0563
positive T cell selection	BP	GO:0043368	11	1772	9	7.05E-05	0.0752
excitatory synapse	CC	GO:0060076	20	2996	11	1.98E-04	0.142
regulation of transmission of nerve impulse	BP	GO:0051969	26	3065	11	2.40E-04	0.142
tissue homeostasis	BP	GO:0001894	56	4702	14	2.46E-04	0.142
positive regulation of membrane potential	BP	GO:0045838	18	2167	9	3.08E-04	0.142
Wnt signaling pathway, planar cell polarity pathway	BP	GO:0060071	14	1724	8	3.20E-04	0.142
thymic T cell selection	BP	GO:0045061	18	2190	9	3.33E-04	0.142
Rho guanyl-nucleotide exchange factor activity	MF	GO:0005089	50	7267	17	8.67E-04	0.312
beta-catenin binding	MF	GO:0008013	42	5365	14	8.78E-04	0.312
cytoskeleton organization	BP	GO:0007010	181	20669	35	0.00112	0.368
regulation of membrane potential	BP	GO:0042391	113	13187	25	0.00127	0.388
regulation of Rho protein signal transduction	BP	GO:0035023	27	3306	10	0.00162	0.447
homophilic cell adhesion	BP	GO:0007156	78	9178	19	0.00180	0.447
Rho GTPase activator activity	MF	GO:0005100	17	1767	7	0.00183	0.447
cell-cell adhesion	BP	GO:0016337	196	21340	35	0.00189	0.447
catalytic step 2 spliceosome	CC	GO:0071013	63	49106	67	0.00280	0.528
Rac GTPase activator activity	MF	GO:0030675	10	986	5	0.00290	0.528
calcium-mediated signaling	BP	GO:0019722	37	4238	11	0.00313	0.528

adult behavior	BP	GO:0030534	85	9002	18	0.00335	0.528
regulation of defense response	BP	GO:0031347	38	3673	10	0.00340	0.528
positive regulation of Rho GTPase activity	BP	GO:0032321	38	3677	10	0.00343	0.528
regulation of systemic arterial blood pressure	BP	GO:0003073	19	1988	7	0.00349	0.528
regulation of smooth muscle contraction	BP	GO:0006940	25	1989	7	0.00350	0.528
pallium development	BP	GO:0021543	68	9048	18	0.00353	0.528
regulation of receptor activity	BP	GO:0010469	46	5611	13	0.00362	0.528
regulation of Rho GTPase activity	BP	GO:0032319	40	5648	13	0.00382	0.528
spliceosomal complex	CC	GO:0005681	99	51570	69	0.00383	0.528
Ras guanyl-nucleotide exchange factor activity	MF	GO:0005088	74	9150	18	0.00395	0.528
positive regulation of muscle contraction	BP	GO:0045933	14	1065	5	0.00400	0.528
terminal bouton	CC	GO:0043195	37	5066	12	0.00427	0.528
signal transduction	BP	GO:0007165	604	64505	83	0.00437	0.528
negative T cell selection	BP	GO:0043383	10	677	4	0.00445	0.528
negative thymic T cell selection	BP	GO:0045060	10	677	4	0.00445	0.528
positive regulation of Rac GTPase activity	BP	GO:0032855	24	2097	7	0.00463	0.535
cAMP binding	MF	GO:0030552	17	2720	8	0.00538	0.604
positive regulation of smooth muscle contraction	BP	GO:0045987	17	1204	5	0.00662	0.674
negative regulation of blood pressure	BP	GO:0045776	25	2248	7	0.00667	0.674
regulation of axon extension	BP	GO:0030516	25	3437	9	0.00677	0.674
channel regulator activity	MF	GO:0016247	51	5384	12	0.00678	0.674
cyclic nucleotide binding	MF	GO:0030551	25	3447	9	0.00689	0.674
extracellular matrix structural constituent	MF	GO:0005201	23	2301	7	0.00752	0.674
taxis	BP	GO:0042330	39	2304	7	0.00757	0.674
regulation of Ras GTPase activity	BP	GO:0032318	51	7574	15	0.00760	0.674
cardiocyte differentiation	BP	GO:0035051	31	4142	10	0.00764	0.674
amide transport	BP	GO:0042886	10	802	4	0.00797	0.674
positive regulation of ion transmembrane transporter activity	BP	GO:0032414	10	1262	5	0.00801	0.674

receptor activity	MF	GO:0004872	114	9089	17	0.00802	0.674
intraspecies interaction between organisms	BP	GO:0051703	21	2341	7	0.00822	0.674
social behavior	BP	GO:0035176	21	2341	7	0.00822	0.674
regulation of dendrite morphogenesis	BP	GO:0048814	33	3629	9	0.00942	0.758
nicotinamide nucleotide biosynthetic process	BP	GO:0019359	12	850	4	0.00970	0.764
positive regulation of synaptic transmission, glutamatergic	BP	GO:0051968	14	1329	5	0.00985	0.764
SH3/SH2 adaptor activity	MF	GO:0005070	13	861	4	0.0101	0.767
Sertoli cell differentiation	BP	GO:0060008	14	1344	5	0.0103	0.767
Sertoli cell development	BP	GO:0060009	11	874	4	0.0106	0.767
postsynaptic membrane organization	BP	GO:0001941	12	1362	5	0.0109	0.767
signaling adaptor activity	MF	GO:0035591	24	3102	8	0.0113	0.767
NAD biosynthetic process	BP	GO:0009435	13	894	4	0.0115	0.767
cell division	BP	GO:0051301	85	52471	67	0.0116	0.767
epithelial cell migration	BP	GO:0010631	20	3122	8	0.0117	0.767
endothelial cell migration	BP	GO:0043542	21	3125	8	0.0117	0.767
regulation of synaptic transmission, glutamatergic	BP	GO:0051966	28	2512	7	0.0117	0.767
regulation of cholesterol transport	BP	GO:0032374	11	903	4	0.0119	0.767
regulation of inhibitory postsynaptic membrane potential	BP	GO:0060080	10	922	4	0.0127	0.810
14-3-3 protein binding	MF	GO:0071889	13	2591	7	0.0137	0.850
regulation of potassium ion transmembrane transport	BP	GO:1901379	11	1447	5	0.0138	0.850
single-organism behavior	BP	GO:0044708	98	10456	18	0.0141	0.852
cochlea development	BP	GO:0090102	27	3237	8	0.0142	0.852
regulation of establishment of planar polarity	BP	GO:0090175	12	1481	5	0.0150	0.892
fat cell differentiation	BP	GO:0045444	65	6031	12	0.0153	0.896
sarcomere organization	BP	GO:0045214	18	2058	6	0.0156	0.897
negative regulation of cell migration	BP	GO:0030336	71	9054	16	0.0160	0.910
regulation of system process	BP	GO:0044057	40	3335	8	0.0166	0.929

cell adhesion molecule binding	MF	GO:0050839	48	6116	12	0.0169	0.929
sialyltransferase activity	MF	GO:0008373	10	1007	4	0.0170	0.929
keratin filament	CC	GO:0045095	31	1020	4	0.0177	0.954
non-canonical Wnt signaling pathway	BP	GO:0035567	16	2130	6	0.0181	0.954
myofibril assembly	BP	GO:0030239	25	2746	7	0.0181	0.954
proteasome core complex	CC	GO:0005839	20	1031	4	0.0183	0.954
potassium ion transmembrane transport	BP	GO:0071805	42	4114	9	0.0196	0.989
neurotransmitter metabolic process	BP	GO:0042133	14	1056	4	0.0198	0.989
threonine-type peptidase activity	MF	GO:0070003	20	1065	4	0.0203	0.989
threonine-type endopeptidase activity	MF	GO:0004298	20	1065	4	0.0203	0.989
potassium channel regulator activity	MF	GO:0015459	19	1603	5	0.0203	0.989
membrane hyperpolarization	BP	GO:0060081	19	1605	5	0.0204	0.989
cell activation	BP	GO:0001775	43	3475	8	0.0206	0.989
negative regulation of cellular carbohydrate metabolic process	BP	GO:0010677	10	1082	4	0.0214	1
single-organism cellular process	BP	GO:0044763	886	118263	134	0.0230	1
inner ear receptor cell development	BP	GO:0060119	17	1669	5	0.0237	1
cellular transition metal ion homeostasis	BP	GO:0046916	43	2910	7	0.0239	1
second-messenger-mediated signaling	BP	GO:0019932	44	4273	9	0.0241	1
endocytic vesicle	CC	GO:0030139	57	5709	11	0.0242	1
limbic system development	BP	GO:0021761	49	6455	12	0.0243	1
positive regulation of endocytosis	BP	GO:0045807	42	4284	9	0.0245	1
axis elongation	BP	GO:0003401	10	1137	4	0.0251	1
regulation of myeloid cell differentiation	BP	GO:0045637	26	3621	8	0.0255	1
transmembrane transport	BP	GO:0055085	148	11301	18	0.0276	1
neuron maturation	BP	GO:0042551	17	2365	6	0.0282	1
learning	BP	GO:0007612	51	7380	13	0.0285	1
plasma membrane part	CC	GO:0044459	914	83415	97	0.0286	1
cellular iron ion homeostasis	BP	GO:0006879	29	1757	5	0.0286	1

proteinaceous extracellular matrix	CC	GO:0005578	191	18886	27	0.0286	1
axon terminus	CC	GO:0043679	72	8964	15	0.0291	1
actin filament-based process	BP	GO:0030029	94	10576	17	0.0294	1
aspartate family amino acid biosynthetic process	BP	GO:0009067	11	694	3	0.0302	1
nucleotide metabolic process	BP	GO:0009117	18	1210	4	0.0304	1
complement activation	BP	GO:0006956	24	697	3	0.0305	1
negative regulation of transport	BP	GO:0051051	27	3071	7	0.0306	1
enteric nervous system development	BP	GO:0048484	14	2417	6	0.0308	1
positive regulation of dendrite morphogenesis	BP	GO:0050775	15	1797	5	0.0311	1
Rho protein signal transduction	BP	GO:0007266	22	2422	6	0.0311	1
myoblast fusion	BP	GO:0007520	13	1220	4	0.0312	1
cell chemotaxis	BP	GO:0060326	31	3773	8	0.0313	1
cochlea morphogenesis	BP	GO:0090103	14	1802	5	0.0314	1
cellular nitrogen compound metabolic process	BP	GO:0034641	13	707	3	0.0316	1
regulation of potassium ion transmembrane transporter activity	BP	GO:1901016	12	1236	4	0.0325	1
post-anal tail morphogenesis	BP	GO:0036342	20	2451	6	0.0326	1
syncytium formation by plasma membrane fusion	BP	GO:0000768	15	1246	4	0.0333	1
positive regulation of cellular component biogenesis	BP	GO:0044089	15	1834	5	0.0334	1
positive regulation of nervous system development	BP	GO:0051962	15	1834	5	0.0334	1
regulation of synapse organization	BP	GO:0050807	16	1838	5	0.0337	1
regulation of ion transport	BP	GO:0043269	55	5297	10	0.0344	1
spermatid differentiation	BP	GO:0048515	61	5335	10	0.0358	1
leukocyte chemotaxis	BP	GO:0030595	23	1871	5	0.0359	1
positive regulation of developmental growth	BP	GO:0048639	32	4603	9	0.0361	1
negative regulation of sequence-specific DNA binding transcription factor activity	BP	GO:0043433	86	10041	16	0.0362	1
regulation of G2/M transition of mitotic cell cycle	BP	GO:0010389	14	1282	4	0.0364	1
positive regulation of synapse assembly	BP	GO:0051965	16	1878	5	0.0364	1

toll-like receptor signaling pathway	BP	GO:0002224	19	1288	4	0.0369	1
nucleotide biosynthetic process	BP	GO:0009165	18	1890	5	0.0372	1
cardiac myofibril assembly	BP	GO:0055003	11	1892	5	0.0374	1
single organism signaling	BP	GO:0044700	296	32746	42	0.0375	1
regulation of lipid metabolic process	BP	GO:0019216	36	3958	8	0.0396	1
regulation of dendritic spine development	BP	GO:0060998	15	2577	6	0.0400	1
lung epithelial cell differentiation	BP	GO:0060487	22	3262	7	0.0402	1
regulation of small GTPase mediated signal transduction	BP	GO:0051056	32	4700	9	0.0402	1
hippocampus development	BP	GO:0021766	42	5448	10	0.0403	1
negative regulation of cell-substrate adhesion	BP	GO:0010812	21	1938	5	0.0407	1
protein complex scaffold	MF	GO:0032947	30	3995	8	0.0414	1
protein activation cascade	BP	GO:0072376	13	795	3	0.0423	1
mesoderm formation	BP	GO:0001707	31	4749	9	0.0424	1
lamellipodium	CC	GO:0030027	84	10261	16	0.0424	1
myeloid leukocyte migration	BP	GO:0097529	13	1349	4	0.0425	1
muscle structure development	BP	GO:0061061	42	4049	8	0.0441	1
organic acid binding	MF	GO:0043177	10	810	3	0.0442	1
extracellular matrix	CC	GO:0031012	197	18836	26	0.0443	1
negative regulation of cell motility	BP	GO:2000146	52	6312	11	0.0443	1
oligodendrocyte differentiation	BP	GO:0048709	34	4056	8	0.0445	1
ferrous iron binding	MF	GO:0008198	11	816	3	0.0450	1
cardiovascular system development	BP	GO:0072358	170	20632	28	0.0452	1
positive regulation of axonogenesis	BP	GO:0050772	33	5565	10	0.0452	1
dendrite	CC	GO:0030425	309	37846	47	0.0457	1
sensory perception of chemical stimulus	BP	GO:0007606	39	3367	7	0.0461	1
cellular response to stimulus	BP	GO:0051716	290	30549	39	0.0463	1
positive regulation of blood pressure	BP	GO:0045777	21	1389	4	0.0464	1
cellular catabolic process	BP	GO:0044248	110	7152	12	0.0465	1

antigen receptor-mediated signaling pathway	BP	GO:0050851	46	4096	8	0.0466	1
dystrophin-associated glycoprotein complex	CC	GO:0016010	10	837	3	0.0479	1
G-protein coupled receptor signaling pathway, coupled to cyclic nucleotide second messenger	BP	GO:0007187	27	2041	5	0.0488	1
cellular aromatic compound metabolic process	BP	GO:0006725	37	2042	5	0.0489	1
associative learning	BP	GO:0008306	44	5659	10	0.0495	1

Supplementary Table 4: Depolarization changes induced by K+ at non-CpG sites. A consistent change > 20% in DNA methylation across replicates was identified at 34 non-CpG sites. A proportion of these changes were subsequently blocked in the presence of either Nifedipine or FK506. Site location is from rn4. Gene annotation gives the rat genome database id; if a site was intergenic the nearest gene within 10kb was identified.

Chromosome	BP	Median read depth	Depolarization change pair 1	Depolarization change pair 2	Blocked by Nifedipine	Blocked by FK506	Gene annotation	CpG island annotation
chr12	23670081	44	-46.35	-42.36	No	No	Intron:1307435	
chr8	79534420	41.5	-41.03	-33.02	No	No		
chr8	51142389	76	29.59	47.19	No	No		
chr19	38666315	24	40.08	31.16	No	Yes	Intron:1560268	
chr12	7877334	21	28.57	41.43	No	Yes	Intron:2621	
chr1	82451928	20.5	-29.22	-32.14	No	Yes	Exon:1304682	CpG Shore
chr7	1.35E+08	50.5	-26.47	-38.02	No	No		
chr18	49951353	15.5	37.50	27.27	Yes	No		
chr4	57922301	11.5	-35.80	-27.27	Yes	Yes	Intron:1566215	
chr18	70274125	23	29.03	29.85	Yes	Yes		
chr10	72868409	71	25.56	29.74	No	No		
chr17	14373281	29	-43.75	-24.00	No	No		CpG:_70
chr13	97486799	13	30.00	25.00	Yes	Yes	Intron:1582859	
chr20	4281974	15.5	30.25	23.81	Yes	Yes	Intron:1307063 | 1303282	
chr9	39636441	41	-24.83	-26.77	No	No	Intron:1308589	
chr13	51598955	30.5	-27.94	-23.71	No	No		
chr5	75105147	20	69.23	21.97	No	No	TES:1311363	
chr16	80474954	12.5	-21.67	-31.54	Yes	No		
chr11	78797618	18	23.53	26.32	Yes	Yes		
chr20	46237513	20	39.30	21.72	Yes	Yes		
chr11	31531052	34.5	-20.61	-41.56	No	No	TES:1307532 | 1307561	
chr4	1.75E+08	21.5	20.57	41.90	No	Yes	Closest gene:1310590	

chr10	94550949	24.5	25.71	23.39	Yes	Yes		
chr12	28698719	23	20.83	31.82	Yes	Yes		
chr18	18928498	25	22.06	26.00	Yes	Yes		
chrX	18553987	16	21.40	25.00	Yes	Yes		
chr5	1.56E+08	24	-20.34	-26.85	Yes	Yes	Intron:1564232	
chr5	62025491	14	-23.41	-22.31	Yes	Yes	Intron:1565350	
chr14	89227865	35.5	23.49	21.85	Yes	Yes	TSS:1564724	CpG Shore
chr3	1.63E+08	20	-20.43	-24.60	Yes	No		
chr12	37330773	19	20.83	23.89	Yes	Yes	Intron:71079	CpG Shore
chr1	1.96E+08	46.5	-25.29	-20.43	No	No		
chr5	1.71E+08	25	21.00	22.67	Yes	Yes		CpG:_149
chrX	1.23E+08	16.5	-21.21	-22.22	Yes	No		
